# Discovery of Small-Molecule Antagonists of Orexin 1/2 Receptors from Traditional Chinese Medicinal Plants with a Hypnotic Effect

**DOI:** 10.3390/ph16040542

**Published:** 2023-04-04

**Authors:** Jia He, Jing Fang, Yuxin Wang, Chengyu Ge, Shao Liu, Yueping Jiang

**Affiliations:** 1 Department of Pharmacy, Xiangya Hospital, Central South University, Changsha 410008, China; 2 Institute for Rational and Safe Medication Practices, National Clinical Research Center for Geriatric Disorders, Xiangya Hospital, Central South University, Changsha 410008, China; 3 College of Pharmacy, Dali University, Dali 671000, China

**Keywords:** traditional Chinese medicine (TCM), orexin receptors, in-home ligand library, neferine, screening and verification

## Abstract

Insomnia is an important public health problem. The currently available treatments for insomnia can cause some adverse effects. Orexin receptors 1 (OX_1_R) and 2 (OX_2_R) are burgeoning targets for insomnia treatment. It is an effective approach to screening OX_1_R and OX_2_R antagonists from traditional Chinese medicine, which contains abundant and diverse chemical components. This study established an in-home ligand library of small-molecule compounds from medicinal plants with a definite hypnotic effect, as described in the Chinese Pharmacopoeia. Molecular docking was applied to virtually screen potential orexin receptor antagonists using molecular operating environment software, and surface plasmon resonance (SPR) technology was used to detect the binding affinity between potential active compounds and orexin receptors. Finally, the results of virtual screening and SPR analysis were verified through in vitro assays. We successfully screened one potential lead compound (neferine) as an orexin receptor antagonist from the in-home ligand library, which contained more than 1000 compounds. The screened compound was validated as a potential agent for insomnia treatment through comprehensive biological assays. This research enabled the discovery of a potential small-molecule antagonist of orexin receptors for the treatment of insomnia, providing a novel screening approach for the detection of potential candidate compounds for corresponding targets.

## 1. Introduction

Insomnia is one of the most common health problems in the general population and in clinical practice [1]. The overall prevalence of insomnia symptoms ranges from 30% to 48% among the elderly [2,3]. Approximately 50% of older adults have difficulty in initiating or maintaining sleep [3,4]. The functional consequences of sleep deprivation and chronic insomnia include depression symptoms, hypertension, myocardial infarction, stroke, and metabolic syndrome [5,6,7]. Insomnia treatment can be divided into nonpharmacological and pharmacological approaches [8]. Patients with chronic insomnia symptoms may need pharmacotherapy. Currently available pharmacotherapies include benzodiazepine sedatives, non-benzodiazepine hypnotics, antidepressants, antihistamines, and melatonin receptor agonists [9,10,11]. However, the long-term use of benzodiazepine and non-benzodiazepine sedatives could lead to physical tolerance issues, addiction, rebound insomnia, residual daytime sedation, and a lack of motor co-ordination [12,13,14]. Considering these side effects, it is important to identify new drugs with fewer adverse effects and novel mechanisms for the clinical treatment of insomnia.

Recently, the orexin system has been reported as a potential target for a new class of sleep medication [15]. Orexins, also known as hypocretins, are excitatory neuropeptides secreted by neurons located particularly in the lateral hypothalamus and perifornical areas [16,17]. Orexin peptides (orexin A and orexin B) selectively bind to orexin receptors 1 and 2 (OX_1_R and OX_2_R), respectively [18,19]. Orexin peptides participate in diverse functions, including cardiovascular responses, heart rate, hypertension, hyperarousal, hyperphagia, and obesity [20,21,22]. Furthermore, orexin peptides play a key role in sleep–wake regulation and have helped to unravel the etiology underlying narcolepsy [23]. Orexin receptor antagonists can effectively induce sleep and maintain a natural sleep–wake cycle without impairing either motor function or the ability to arouse in response to salient stimuli [15].

Suvorexant was the first dual orexin receptor antagonist approved by the Food and Drug Administration, with a prescribed dose of up to 20 mg [24,25]. This drug targets wakefulness-promoting neuropeptides that regulate the sleep–wake cycle and is effective in decreasing sleep latency and increasing total sleep time [25]. These lines of evidences suggest that orexin receptor antagonists are a promising novel drug therapy.

Traditional Chinese medicine (TCM) is a new source of abundant and diverse small-molecule drug candidates, given the highly diverse bioactivities of natural products [26]. Currently available clinical data suggest that several medicinal plants and herbs are most frequently used in TCM due to their hypnotic effects, such as *Plumula nelumbinis*, *Ziziphus jujuba* Mill. var., *Lilium lancifolium* Thumb, *Panax ginseng* C.A. Mey., etc. [27,28]. *Plumula nelumbinis* promotes non-rapid eye movement sleep by regulating GABAergic receptors, as demonstrated in a rat model [28]. The saponins present in *Lilium lancifolium* Thumb extracts also regulate the hypnotic effects of sedatives [29]. Additionally, *Ziziphus jujuba* Mill. var. has been used in TCM to treat insomnia for a long time, because it decreases the activity of the monoaminergic system [30,31,32]. Ginseng glycoproteins exert their sedative–hypnotic effects by causing changes in the brain metabolism [33,34]. However, there are great differences in the structures of chemical components in TCM plants or herbs, which may act on different targets to exert their hypnotic effect. Thus, there is an urgent need to discover novel orexin receptor antagonists from TCM plants or herbs for insomnia treatment, and a new molecular framework can provide clinical studies on insomnia with more feasibility. In this regard, a considerable current challenge is to find a method for quickly screening the potential orexin receptors antagonists from these TCMs.

In this study, we aimed to quickly screen for small-molecule antagonists to orexin receptors from TCM plants or herbs with hypnotic effects. For this purpose, we prepared a framework of virtual screening through molecular docking, followed by surface plasmon resonance (SPR) and gene chip analyses to detect the binding affinity between potential active compounds and orexin receptors (OX_1_R and OX_2_R). The obtained results were verified through in vitro assays. This framework provides a more efficient method for screening potential active compounds, targets, and pathways. Additionally, this approach enables the discovery of lead compounds for the development of novel anti-insomnia drugs.

## 2. Results

### 2.1. In-Home Ligand Library

Fifteen medicinal plants, including *Plumula Nelumbinis*, *Ziziphus jujuba* mill. Var, *Lilium lancifolium* thumb, and *Panax ginseng* C.A mey, were screened for the treatment of insomnia by conducting a search in the Chinese Pharmacopoeia (Table S1 in the Supporting Materials). A total of 1785 small molecules (the structures of these compounds are given in the Supporting Materials) were collected from the 15 medicinal plants using SciFinder, PubMed, and China National Knowledge Infrastructure (CNKI, https://www.cnki.net/, accessed on 1 December 2022), including alkaloids, flavonoids, saponins, lignans, steroids, and organic acids, which were then used to establish the in-home ligand library.

### 2.2. Molecular-Docking-Based Virtual Screening of Orexin Receptor Antagonists from the In-Home Ligand Library

Molecular docking was applied to investigate the interactions of the candidate small molecules with OX_1_R and OX_2_R. The approved orexin receptor antagonist suvorexant could bind to OX_1_R and OX_2_R; thus, it was set as a positive control. We then predicted the binding affinity (S value) and binding patterns for the interactions of the orexin receptors with the small-molecule compounds; the results are summarized in Table 1. According to the binding affinity (S value) between the ligand and receptor, the top 10 compounds belonged to the bisbenzylisoquinoline alkaloids (the structure of these compounds can be seen in Figure S1 in the Supporting Materials). Since these bisbenzylisoquinoline alkaloids possess nearly the same binding affinity, the most common and dominant active constituent in *P. Nelumbinis*, neferine, was selected to further verify its bioactivity.

**Figure 1:**
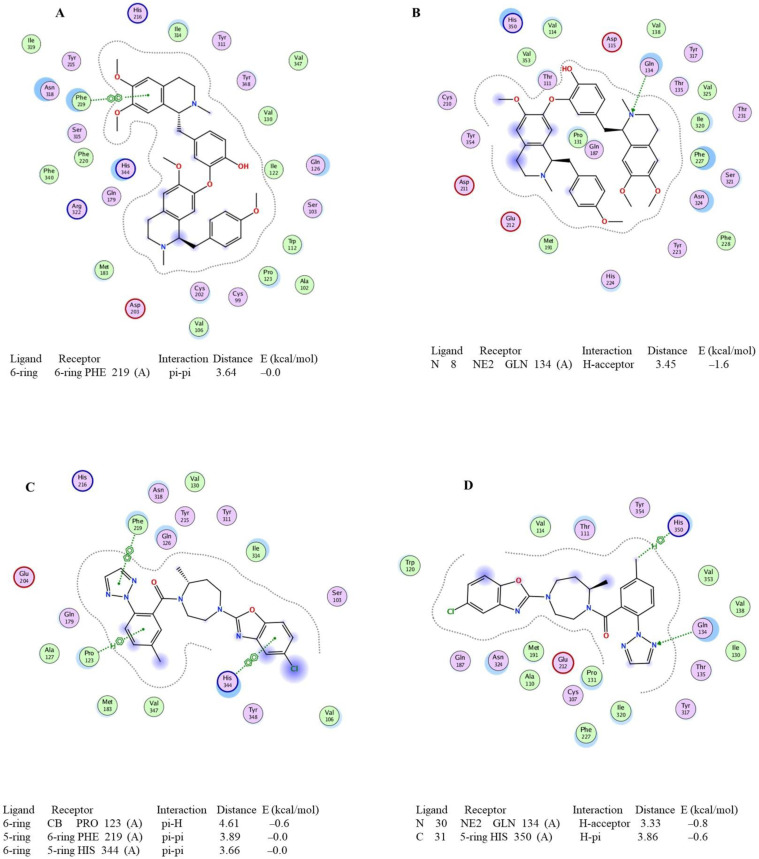
The molecular interactions between the orexin receptors and ligands ((**A**): neferine and orexin 1 receptor; (**B**): neferine and orexin 2 receptor; (**C**): suvorexant and orexin 1 receptor; (**D**): suvorexant and orexin 2 receptor).

The binding affinity of neferine to OX_1_R and OX_2_R was −10.9392 kcal/mol and −11.6055 kcal/mol, respectively, and the corresponding values for suvorexant were −8.2193 kcal/mol and −8.7622 kcal/mol (Table 1). This result suggests that neferine could be a better spatial match with the orexin receptors than suvorexant. In terms of specific interactions between the orexin receptors and ligands, the benzyl ring in neferine (Figure 1A) interacts with the amino acid residue Phe219 through the pi–pi interaction in the binding pocket of OX_1_R (Protein Data Bank (PDB) ID: 4ZJC). The N atom in neferine interacts with Gln134 in OX_2_R (PDB ID: 4S0V) by forming intermolecular hydrogen bonds (Figure 1B). These interactions between the orexin residues and neferine play an important role in the stabilization of this protein–ligand complex. Notably, suvorexant not only establishes a pi–pi interaction between the benzene ring and amino acid residues such as His344 and Phe219 but also has a pi–H interaction between the 1,2,3-trizole ring and Pro123 in OX_1_R (Figure 1C). Additionally, a pi–H interaction also occurs between the methyl moiety of suvorexant and His350 of OX_1_R (Figure 1C). Moreover, similar to neferine, suvorexant exhibits an intermolecular hydrogen bonding force between the N atom of the 1,2,3-trizole ring and Gln134 of OX_2_R (Figure 1D). Based on the molecular docking results, neferine had the best affinity among all the screened compounds; thus, it was chosen as the representative compound for further study.

### 2.3. SPR Analysis of the Binding Affinity between Bisbenzylisoquinoline Alkaloids and OX_1_R and OX_2_R

**Figure 2:**
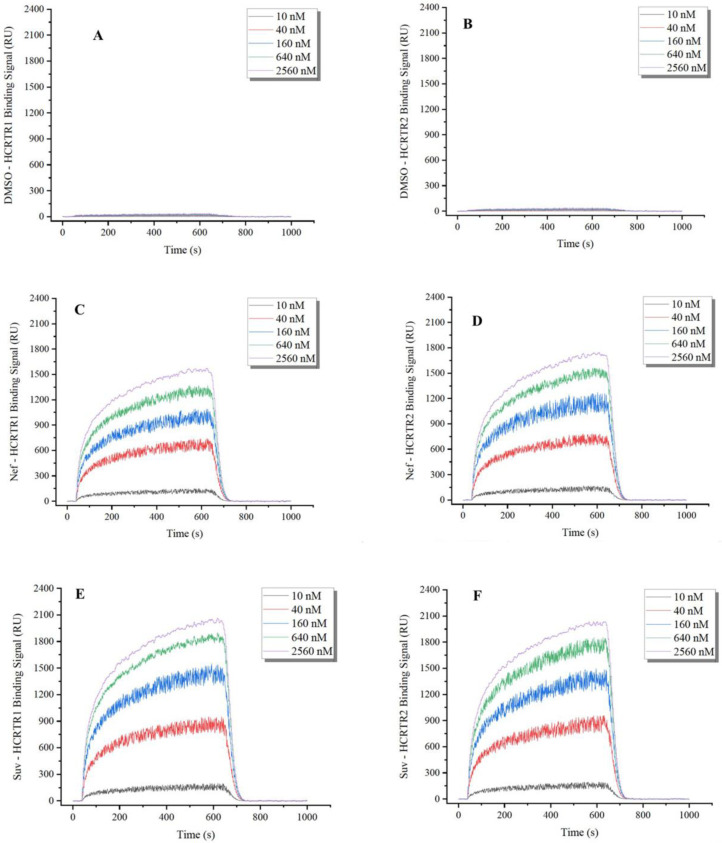
The binding kinetics between orexin receptors and ligands using SPR analysis. (Dynamic binding curves of DMSO (**A**), neferine (**C**), and suvorexant (**E**) interaction with different concentrations of orexin 1 receptor at the resonance wavelength. Dynamic binding curves of DMSO (**B**), neferine (**D**), and suvorexant (**F**) interaction with different concentrations of orexin 2 receptor at the resonance wavelength.)

Measuring binding kinetics using SPR analysis is very important for understanding not only the action duration of a candidate drug but also the difference in therapeutic efficacies between two or more similar drug compounds [35]. In SPR analysis, neferine was used as an extracellular molecular validation model to evaluate small-molecule interactions with OX_1_R and OX_2_R. The kinetics of the binding reaction were determined by injecting different concentrations of OX_1_R and OX_2_R (10, 40, 160, 640, and 2560 nM) over recombinant bisbenzylisoquinoline alkaloids immobilized on a chip surface (Figure 2). The association rate constant (ka), dissociation rate constant (kd), and dissociation constant (K_D_ = kd/ka) were calculated for the candidate compounds under study. Consistent with the molecular docking results, the binding affinity of neferine with OX_1_R and OX_2_R showed high K_D_ values of 2.24 × 10^−9^ M and 1.06 × 10^−8^ M, respectively. Even though the K_D_ values of neferine were higher than those of suvorexant, the binding affinity at the nM level also displayed a strong interaction between neferine and the orexin receptors. The molecular docking results were consistent with those of SPR analysis, suggesting that neferine can strongly bind to orexin receptors. Thus, neferine is a promising lead compound as an orexin receptor antagonist.

### 2.4. Inhibitory Activity of Neferine on OX_1_R and OX_2_R

#### 2.4.1. Systemically Exploring the Candidate Gene Mechanism of Neferine through RNA Sequencing

**Figure 3:**
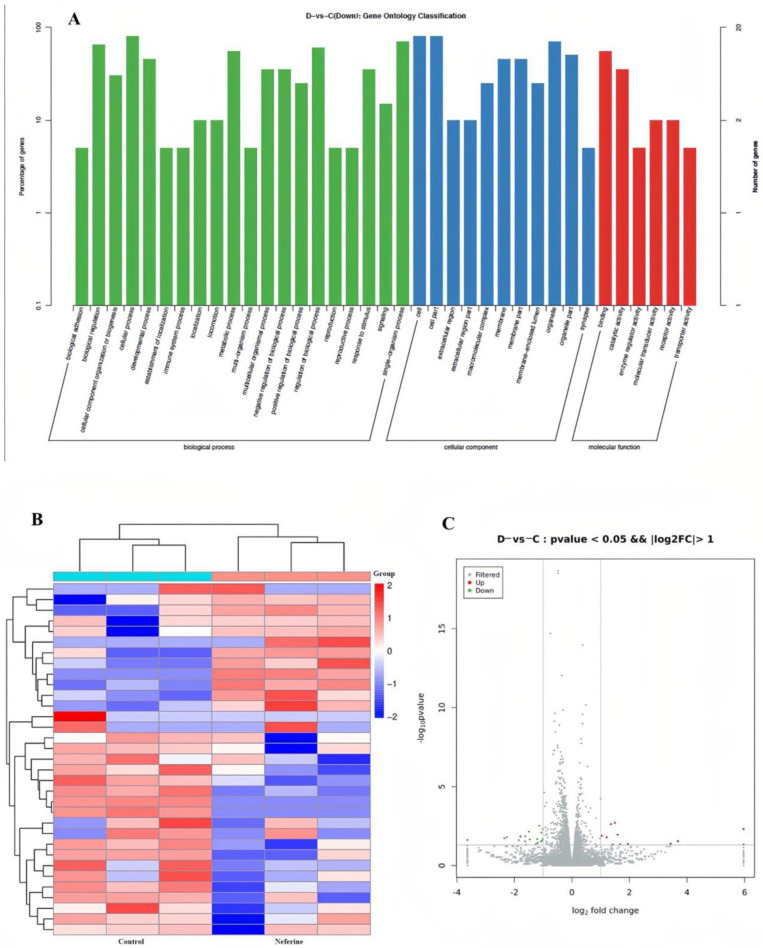
Quantification of transcriptome profiles in CHO cell treated with neferine. ((**A**) GO enrichment analysis of the main biological and cellular process; (**B**) hierarchical clustering and heatmap of the correlation gene expression profiles; (**C**) volcano plot indicated different gene expression profiles.)

To further determine the bioactivity of neferine and the underlying mechanism of orexin receptor regulation through in vitro experiments, neferine (1.0 µM) was administered to a Chinese hamster ovary (CHO) cell. According to the enrichment factor value, some terms could be related to the gene transcription and expression, signal transduction, and development processes (Figure 3A). Total RNA from the cells was isolated and purified, and RNA sequencing was performed. Figure 3B shows a cluster graph constructed based on the cutoff criteria of *p* < 0.05 and fold change ≥ 2. After neferine treatment, the mRNA expression of OX_1_R was downregulated when compared with that in the sham group; however, the mRNA expression of OX_2_R showed slight upregulation (Figure 3C).

#### 2.4.2. Quantitative Reverse Transcription–Polymerase Chain Reaction and Western Blotting of the Orexin Receptor Antagonist Neferine

**Figure 4:**
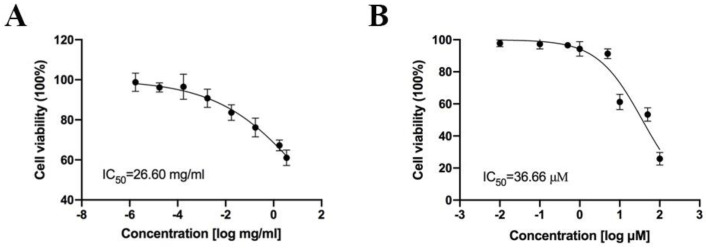
Effects of cell toxicity were assessed via CCK8 assay ((**A**): the viability of CHO cells was determined after treatment with the indicated concentrations of total alkaloids for 24 h; (**B**): the viability of CHO cells was determined after treatment with the indicated concentrations of neferine for 24 h, and the IC_50_ values were determined).

The viability of CHO cells after treatment with the candidate compounds was analyzed using the cell counting kit−8 (CCK−8) assay. Cells were incubated with different concentrations of total alkaloids (3.5 mg/mL to 3.5 × 10^−6^ mg/mL) and neferine (0.01–100 μM) for 24 h. Cell viability decreased after treatment with total alkaloids (1.75 × 10^−2^ mg/mL) when compared with that in the control group at 24 h. Neferine exerted no significant cytotoxic effects at concentrations of up to 5 μM (Figure 4).

**Figure 5:**
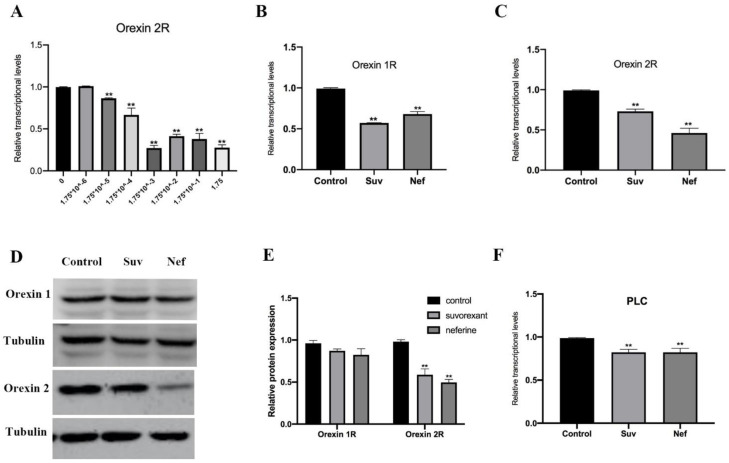
The effect of total alkaloids and neferine on the mRNA expression and protein expression levels of orexin 1, orexin 2, and PLC ((**A**): in vitro assay of total alkaloids inhibiting orexin 2 mRNA expression in a dose-dependent manner [** *p* < 0.01 compared with the control group (the CHO cell line is not treated with total alkaloids)]; (**B**): mRNA expression levels of orexin 1 were determined through real-time PCR assay (** *p* < 0.01 compared with the control group); (**C**): mRNA expression levels of orexin 2 were determined through real-time PCR assay (** *p* < 0.01 compared with the control group); (**D**): CHO cells were treated with neferine for 24 h, and protein expression was detected from these cells; (**E**): the levels of orexin 1 and orexin 2 were quantified via Western blotting, with tubulin antibody as a control (** *p* < 0.01 compared with the control group); (**F**): mRNA expression levels of PLC were determined through real-time PCR assay (** *p* < 0.01 compared with the control group).

To further identify the inhibitory effect of the active candidate compounds on orexin receptors, we subsequently evaluated the mRNA and protein expression levels of OX_1_R and OX_2_R. In the CHO cell line, the mRNA expression of OX_2_R tended to show significant downregulation after treatment with various concentrations of total alkaloids when compared with that in the control group (Figure 5A). Neferine significantly downregulated the mRNA expression of both OX_1_R and OX_2_R in the CHO cells, and the mRNA expression of OX_2_R in the neferine group was more significantly downregulated than that in the suvorexant group within 24 h (Figure 5C), whereas the mRNA expression of OX_1_R was not significantly downregulated (Figure 5B). After the suvorexant and neferine treatment of CHO cells for 24 h, the protein expression of OX_1_R underwent a relative decrease (Figure 5D), while the densitometry data of the OX_2_R protein level normalized with GAPDH reveal that the protein level showed a more marked decrease (Figure 5E). These results indicate that neferine has a slightly higher ability to regulate OX_2_R than OX_1_R and, consequently, influence the sleep–wake function. To analyze the regulatory effect of phospholipase C (PLC) on orexin receptors’ expression during neferine treatment, the relative mRNA expression of PLC was measured using reverse transcription–polymerase chain reaction (RT-PCR) in neferine-treated CHO cells. Treatment with various concentrations of neferine decreased the mRNA expression level of PLC. The inhibitory effect of neferine treatment on PLC mRNA levels occurred in a dose-dependent manner for 24 h (Figure 5F). The results of the RT-PCR analysis suggest that neferine treatment lowers the transcription of orexin receptors, while it quickly decreases the transcription of PLC within 24 h, indicating that neferine antagonizes the orexin receptors and might exert a sedative and hypnotic effect as an orexin receptor antagonist.

## 3. Discussion

Insomnia is a serious public health problem worldwide. Orexin receptor antagonists exert good pharmacological and therapeutic effects in the modern clinical treatment of insomnia. TCM has been considered as a source of anti-insomnia candidate drugs, as most of the plants used in TCM have rich structural diversity, with some having documented uses for the treatment of insomnia. In the present study, virtual screening and in vitro validation were performed to discover potential orexin receptor antagonists from TCM plants or herbs. We first established an in-home ligand library containing 1785 compounds identified from 15 TCM plants and herbs and then conducted molecular docking to virtually screen for orexin receptor antagonists from the library. Finally, SPR, RT-PCR, and Western blot assays were furnished to verify the virtual screening results. We discovered that bisbenzylisoquinoline alkaloids from the traditional sedative TCM plant *P. nelumbinis* were potential antagonists of orexin receptors. Our research strategy will offer an approach to discovering potential lead compounds for the treatment of insomnia and lay the foundation for further in vitro and in vivo experiments.

In this study, molecular docking analysis showed that the bisbenzylisoquinoline alkaloids in *P. nelumbinis* possessed great binding affinity with orexin receptors. These compounds are therefore promising lead compounds with enhanced affinity and selectivity toward drug targets. Importantly, these bisbenzylisoquinoline alkaloids could be a better spatial match as orexin receptors antagonists than suvorexant. The pi–pi interaction between the benzene ring in bisbenzylisoquinoline alkaloids and the OX_1_R amino acid residues and the formation of intermolecular hydrogen bonds in the OX_2_R pocket play important roles in the stabilization of the protein–ligand complex. Compared with monobenzylisoquinoline alkaloids, bisbenzylisoquinoline alkaloids displayed stronger binding affinity with the orexin receptors, demonstrating that the relatively higher molecule weight (approximately 600 Da) and the larger spatial structure are more suitable for the active OX_1_R and OX_2_R pockets. It was speculated that the N atom and benzene ring in ligands might be pharmacophores by analyzing the interaction between bisbenzylisoquinoline alkaloids and amino acid residues in orexin receptors. Therefore, other non-alkaloids may be not good potential orexin receptor antagonists. On account of the similar binding affinity with orexin receptors of these bisbenzylisoquinoline alkaloids, it is impossible to judge the effect of small structural changes in bisbenzylisoquinoline alkaloids on the activity. These results will also provide guidance for the subsequent structural modification and optimization of bisbenzylisoquinoline alkaloids by increasing the interaction force.

Stemming from the results of virtual screening, we then verified these predicted potential orexin receptor antagonists through SPR analysis, RT-PCR, and Western blot assays. The great affinity values at the nM level confirmed the strong interaction between neferine and orexin receptors. The inhibitory effects of neferine treatment on the PLC mRNA levels and protein expression levels of OX_1_R and OX_2_R in CHO cells were also detected via RT-PCR and Western blot assays, which served as proof of the inhibitory activity of neferine on OX_1_R and OX_2_R. It could be found from the result of RT-PCR and Western blot assays that the antagonist effect of neferine on OX_2_R was greater than that of OX_1_R. The above strategies are expected to result in the discovery of an efficient and universal verification method for orexin receptor antagonists. However, the anti-insomnia effect should be further evaluated by testing the effect of potential small-molecule antagonist treatment on the sleep of rats experiencing sleep deprivation.

Compared with traditional methods, such as the high-throughput screening method or the method of collecting ligands from a commercial library of small-molecule compounds, our method can quickly lock down potential antagonist molecules and provide pertinent confirmations, because in our method, a large number of ligands were collected from TCMs with definite hypnotic effects. This strategy not only greatly reduces the research cost, but also improves the efficiency of antagonist discovery. We believe this will pave the way for the discovery of antagonists or agonists for other targets. Of course, the available co-crystal structure of target proteins and the accuracy of molecular docking are inevitable as prerequisites.

Through transcriptome analysis, the possible pathway by which neferine exerts its hypnotic effect was speculated. Consistently, the results show that OX_1_R and OX_2_R decreased to some extent. Moreover, a series of targets (such as Pth1r and Ptch2 in Figure 3) were involved in cell development and growth, suggesting the important role of neferine in modulating cell development. Future research could be conducted in this regard. The mRNA and protein expression levels of OX_1_R and OX_2_R were decreased after neferine treatment, with a greater marked decrease in OX_2_R expression. Based on the aforementioned findings, neferine has the potential to improve insomnia symptoms by regulating the orexin receptors. However, further evaluation is still needed to confirm the influx of neferine through nonselective cation channels and voltage-gated calcium channels, all of which could supply an activation signal for PLC [36]. Thus, orexin-regulated sources of Ca^2+^ should be further considered. Moreover, future studies should assess whether neferine plays a critical role in sleep disorders by affecting the hypocretin system, particularly in an orexin-knockout mice model. It is worth noting that neferine displayed diverse biological action, including anti-cancer, anti-diabetic, anti-aging, anti-microbial, anti-thrombotic, anti-arrhythmic, anti-inflammatory, and even anti-HIV effects [37]. Therefore, to avoid clinical side effects in future drug development, the structural modification of neferine is inevitable.

## 4. Materials and Methods

### 4.1. Plant Materials, Chemicals, and Reagents

*Plumula nelumbinis*, the seed embryo of *Nelumbo nucifera* Gaertn. (named “Lian Zi Xin” in Chinese), has been widely used in traditional Chinese medicine (TCM). *Plumula nelumbinis* was purchased from the Xiangtan District (Xiangtan, China) in 2016. The plant materials were authenticated by the authors, and a voucher specimen (ID: 2016001) was deposited at the authors’ laboratory. Total alkaloids and neferine were prepared at the authors’ laboratory [38]. Suvorexant was purchased from Aladdin Biochemical Technology Co., Ltd. (Shanghai, China). OX_1_R and OX_2_R (yeast) were purchased from MyBioSource Inc. (San Diego, CA, USA).

### 4.2. Establishment of an In-Home Ligand Library

“Shi mian” or “Bu mian” in Chinese means insomnia. TCM-related medicinal plants with definite hypnotic effects were identified through a literature search in the Chinese Pharmacopoeia, with “Shi mian” and “Bu mian” as the search keywords. Next, the literature search was performed in PubMed and CNKI using “the name of the screened traditional medicinal plant” and “chemical component” as keywords. This search revealed information about the small-molecule compounds present in the screened medicinal plants, which were used to establish the ligand library.

### 4.3. Virtual Screening Using Molecular Docking Technology

Chemical compounds and target proteins were selected for subsequent molecular docking. The receptor protein coded by the selected gene was searched for in PDB. The three-dimensional (3D) structures of the receptor proteins that inhibited OX_1_R (PDB ID: 4ZJC) and OX_2_R (PDB ID: 4S0V) were downloaded. The 3D structures of chemical compounds were calculated and exported by minimizing energy through ChemBio 3D software. The 3D structure of the receptor protein and molecular operating environment (MOE) software were used to perform hydrogenation and charge calculations for the proteins. The parameters of the receptor protein docking site were set to include the active pocket sites to which small-molecule ligands bind. The detailed parameters were set as follows: placement: triangle matcher; refinement: induced; London dG: 30; GBVI/WSA dG: 5. Other parameters were kept as the default. Finally, according to the docking sites and the molecule ligand binding intensity, the top 10 compounds that showed high activity were screened and documented.

### 4.4. Surface Plasmon Resonance Assay

SPR analysis was performed using the open SPR instrument (bScreen LB 991; Berthold Technologies). Neferine and suvorexant samples were dissolved in dimethyl sulfoxide at different concentrations, and phosphate-buffered saline with Tween 20 (PBST) was used as a running buffer. The procedure was performed as follows: The buffer was run at the maximum flow rate and the bubble was exhausted after reaching the baseline signal. HCl (10 mM) was injected to clean the chip surface and run for 1 min. The flow rate of the buffer solution (PBST) was slowed to 20 μL/min, after which, 200 μL of ethyl-3(3-dymethylamino) propyl carbodiimide (400 mM)/N-hydroxysuccinimide (100 mM) solution (1:1) was loaded to activate the COOH sensor chips and run for 4 min. Neferine, orexin 1 protein, and orexin 2 protein were diluted with activation buffer. The injection port was rinsed with the buffer solution, and air was removed. Subsequently, 200 μL of blocking solution (20 μL/min, 4 min) was added, the sample ring was washed with the buffer solution, and air was removed. The baseline was observed for 5 min to ensure stability. Next, the selected candidate compounds were serially diluted into solutions of different concentrations, which were then injected into the chip, from low to high concentrations. The kinetic parameters of the binding reactions were calculated and analyzed using TraceDrawer software (Ridgeview Instruments AB, Uppsala, Sweden).

### 4.5. Cell Lines and Cell Culture

CHO cells were purchased from the American Type Culture Collection (Frederick, MD, USA). Cells were cultured in Dulbecco’s Modified Eagle Medium (HyClone, GE HealthCare Life Sciences, Beijing, China), a growth medium, supplemented with 10% fetal bovine serum (Gibco, Life Technologies, New York, NY, USA), penicillin (100 μg/mL), and streptomycin (100 μg/mL) in a 5% CO_2_ humidified atmosphere at 37 °C. The cells were subcultured every 2 days or when they reached 90% confluency.

### 4.6. Cell Counting Kit-8 Assay

After the cell cultures in 96-well plates were treated, cell viability was estimated using the CCK-8 assay kit (KeyGEN Biotech, Nanjing, China), according to the manufacturer’s instructions. Briefly, the total alkaloids and neferine were added to each well at various concentrations, after which, 10 μL of the CCK-8 reagent was added, and the plates were incubated for 2 h. The optical density was measured at 490 nm wavelength using a microplate reader (BioTek Instruments, Winooski, VT, USA) to determine cell viability.

### 4.7. Quantitative Reverse Transcription and Real-Time Polymerase Chain Reaction

The mRNA expression of hypocretin was estimated using real-time RT-PCR according to the method described previously [39]. Trizol solution (TaKaRa, Aichi Ken, Japan) was used to isolate total RNA from samples. Then, using a reverse transcription kit, 1.0 μg of total RNA was transcribed into cDNA according to the supplier’s instructions. RT-PCR analysis was carried out using SYBR^®^ Green PCR Master Mix (Invitrogen, Warrington, UK). The β-actin gene was used as an endogenous control for the normalization of the sample. The primers used for OX_1_R were as follows: forward 5′-CCTGGCTGAAGTGAAGCAGA-3′ and reverse 5′-CTGATGGGCAGGTAGCAGAG-3′; OX_2_R, forward 5′-forward 5′-TCGCAACTGGTCATCTGCTT-3′ and reverse 5′-CTCGTCGTCATAGTCGGTGG-3′; β-actin, forward 5′-ATCTGGCACCACACCTTCTACAATGAGCTGCG-3′ and reverse 5′-CGTCATACTCCTGCTTGCTGATCCACATCTGC-3′; PLC, forward 5′-TCGTCCCACAACGAGCA-3′ and reverse 5′-TCGTCCCACAACGAGCA-3′.

### 4.8. Western Blotting

Briefly, cell samples were lysed using 2× sodium dodecyl sulfate (SDS) buffer containing a protein inhibitor. The protein concentration in the cell lysate was measured using a bicinchoninic acid assay kit (Sangon Biotech, Shanghai, China). The extracted proteins were separated using SDS–polyacrylamide gel electrophoresis. The separated proteins were then transferred onto a polyvinylidene difluoride membrane via wet transfer. After the transfer, the membranes were washed with Tris-buffered saline with Tween 20 (TBST) for 10 min and blocked with 5% milk at room temperature for 1 h. After being washed with the TBST solution, the blocked membrane was incubated at 4 °C overnight with appropriately diluted primary antibodies: rabbit anti-orexin 1R, mouse anti-orexin 2R (1:1000 dilution; Cell Signaling Technology, Shanghai, China), and mouse anti-Tubulin (1:1000 dilution; Zhongshan Golden Bridge Biotechnology, Zhongshan, China). The next day, the membrane was washed and incubated with the corresponding secondary antibody (1:5000 dilution; Zhongshan Golden Bridge Biotechnology, Zhongshan, China) for 1 h at room temperature. Finally, the bands were measured using an enhanced chemiluminescence kit (China). Image processing and analysis were carried out using ImageJ software.

### 4.9. Statistical Analysis

The experimental results are expressed as mean ± standard deviation. A One-way analysis of variance was performed using GraphPad Prism (GraphPad Software Inc., San Diego, CA, USA). *p* < 0.05 (*), *p* < 0.01 (**), and *p* > 0.05 (#) indicated statistical significance. All data shown are representative of values from at least three independent experiments.

## 5. Conclusions

In summary, this research provides a new screening approach for the identification of candidate compounds and corresponding targets from an in-home ligand library of small-molecule compounds by combining virtual screening, SPR analysis, and validation tests at the cellular level. This approach helped to reveal novel orexin receptor antagonists for insomnia treatment. Finally, neferine was identified as a potential orexin receptor antagonist, and it is expected to be valuable as a lead compound in the search for and design of new therapeutics for insomnia, as well as other orexin-induced diseases.

## Figures and Tables

**Table 1:** The top 10 binding affinities (S value) between compounds and orexin 1/2 receptors.

No.	Compound Name	Binding Affinity (S Value) ^b^	
		Orexin 1	Orexin 2
Positive control	Suvorexant	−8.2193	−8.7622
**1**	Liensinine	−11.0564	−11.3679
**2**	6-hydroxynorisoliensinine	−10.3332	−11.0539
**3**	*N*-norisoliensinine	−10.4403	−11.3397
**4**	Norisoliensinine	−10.5099	−11.3855
**5**	Isoliensinine	−11.2011	−11.7965
**6**	Neferine	−10.9392	−11.6055
**7**	3‴-hydroxylisoliensine	−10.9323	−11.2588
**8**	3‴-neferine	−11.6650	−11.0768
**9**	Nelumborine A	−10.0248	−10.6367
**10**	Nelumborine B	−10.0467	−10.3183

^b^ The smaller the S value, the stronger the binding affinity of the ligands to the receptors (orexin 1 or orexin 2).

## Data Availability

Data is contained within the article and Supplementary Material.

## Supplementary Materials

The following supporting information can be downloaded at: https://www.mdpi.com/article/10.3390/ph16040542/s1, Figure S1: Fifteen medicinal plants were screened for the treatment of insomnia by searching in Chinese Pharmacopoeia; Figure S1: The structure of ten bisbenzylisoquinoline alkaloids; Figure S2: The original image of western bolts; Table S2: Chemical compositions in *Lilium lancifolium* Thumb; Figure S3: The structure of compounds in *Lilium lancifolium* Thumb; Table S3: Chemical compositions in *Panax ginseng* C.A mey (Part.1); Figure S4: The structure of compounds in *Panax ginseng* C.A mey (Part.1); Table S4: Chemical compositions in *Panax ginseng* C.A mey (Part.2); Figure S5: The structure of compounds in *Panax ginseng* C.A mey (Part.2); Table S5: Chemical compositions in *Panax ginseng* C.A mey (Part.3); Figure S6: The structure of compounds in *Panax ginseng* C.A mey (Part.3); Table S6: Chemical compositions in *Panax ginseng* C.A mey (Part.4); Figure S7: The structure of compounds in *Panax ginseng* C.A mey (Part.4); Table S7: Chemical compositions in *Panax ginseng* C.A mey (Part.5); Figure S8: The structure of compounds in *Panax ginseng* C.A mey (Part.5); Table S8: Chemical compositions in *Panax ginseng* C.A mey (Part.6); Figure S9: The structure of compounds in *Panax ginseng* C.A mey (Part.6); Table S9: Chemical compositions in *Panax ginseng* C.A mey (Part.7); Figure S10: The structure of compounds in *Panax ginseng* C.A mey (Part.7); Table S10: Chemical compositions in *Ziziphus jujuba* mill. Var; Figure S11: The structure of compounds in *Ziziphus jujuba* mill. Var; Table S11: Chemical compositions in *Bambusa tuldoids* munro; Figure S12: The structure of compounds in *Bambusa tuldoids* munro; Table S12: Chemical compositions in *Physochlaina infundibularis* kuang; Figure S13: The structure of compounds in *Physochlaina infundibularis* kuang; Table S13: The main compound in *Ganoderma lucidum*; Figure S14: Chemical structure of compound **1**–**231**; Table S14: Chemical Structures of compounds **1**–**231**; Figure S15: Structures of compounds **232**–**234** Figure S16: Structures of compounds **235**–**236**; Figure S16: Structures of compounds **237**–**240**; Figure S17: Structures of compounds **241**–**244**; Figure S18: Structures of compounds **245**–**249**; Figure S19: Structures of compounds **250**–**252**; Figure S20: Structures of compounds **253**–**254**; Figure S21: Structures of compounds **255**–**256**; Figure S22: Structures of compounds **257**–**260**; Figure S23: Chemical structure of compound **261**–**301**; Table S15: Chemical Structures of compounds **261**–**301**; Figure S24: Structures of compounds **302**–**307**; Figure S25: Structures of compounds **309**–**310**; Figure S26: Structures of compounds **311**–**317**; Figure S27: Structures of compound **318**; Figure S28: Structures of compounds **319**–**322**; Figure S29: Structures of compound **261**–**301**; Table S16: Structures of compounds **261**–**301**; Figure S30: Structures of compounds **396**–**398**; Figure S31: Structures of compounds **399**–**402**; Figure S32: Structures of compounds **403**–**407**; Figure S33: Structures of compounds **408**–**410**; Figure S34: Structures of compounds **411**–**422**; Figure S35: Structures of compounds **423**–**427**; Figure S36: Structures of compounds **428**–**431**; Figure S37: Structures of compound **432**; Figure S38: Structures of compounds **434**–**437**; Figure S39: Structures of compounds **438**–**446**; Figure S40: Structures of compounds **447**–**448**; Figure S41: Structures of compounds **449**–**453**; Figure S42: Structures of compounds **454**–**460**; Figure S43: Structures of compounds **461**–**464**; Figure S44: Structures of compounds **465**–**471**; Figure S45: Structures of compounds **472**–**473**; Figure S46: Structures of compounds **474**–**485**; Figure S47: Structures of compounds **486**–**494**; Figure S48: Structures of compounds **495**–**498**; Table S17: Chemical Structures of compounds **1**–**21**; Figure S49: Chemical structure of compounds **1**–**41**; Table S18: Chemical Structures of compounds **1**–**41**; Figure S50: Chemical structure of compounds **1**–**38**; Table S19: Chemical Structures of compounds **1**–**38**; Figure S51: Chemical structure of compounds **39**–**69**; Table S20: Chemical Structures of compounds **39**–**69**; Figure S52: Chemical structure of compounds **70**–**107**; Table S21: Chemical Structures of compounds **70**–**107**; Table S22: Chemical Structures of compounds **1**–**10**; Figure S53: Chemical structure of compounds **1**–**10**; Table S23: Chemical composition of *Apocynum Venetum*; Figure S54: The chemical structures of *Apocynum Venetum*; Table S24: Chemical composition of *Platycladus orientalis* franco; Figure S55: The chemical structures of *Platycladus orientalis* franco; Table S25: Chemical composition of *Polygonum multiflorum* thumb; Figure S56: The chemical structures of *Polygonum multiflorum* thumb; Table S26: Chemical composition of *Nelumbo nucifera* gaertn; Figure S57: The chemical structures of *Nelumbo nucifera* gaertn. References [40,41,42,43,44,45,46,47,48,49,50,51,52,53,54,55,56,57,58,59,60,61,62,63,64,65,66,67,68,69,70,71,72,73,74,75,76,77] are cited in the supplementary materials.
