# Supplementary material for: Discovery of Small-Molecule Antagonists of Orexin 1/2 Receptors from Traditional Chinese Medicinal Plants with a Hypnotic Effect
Source: Pharmaceuticals (Basel). 2023 Apr 4;16(4):542. doi: 10.3390/ph16040542 (PMC10146837; doi:10.3390/ph16040542)
Supplement: Supplementary file 1 [file pharmaceuticals-16-00542-s001.zip › pharmaceuticals-2251066-supplementary.pdf]

Discovery of small-molecule antagonists of orexin 1/2 receptors from traditional Chinese medicinal plants with a hypnotic effect

Jia He [a,b,1], Jing Fang [a,b,1], Yuxin Wang [c], Chengyu Ge [a,b], Shao Liu [a,b*], Yueping Jiang [a,b*]

[a] *Department of Pharmacy, Xiangya Hospital, Central South University, Changsha, 410008, China*

[b] *Institute for Rational and Safe Medication Practices, National Clinical Research Center for Geriatric Disorders, Xiangya Hospital, Central South University, Changsha,410008, China*

[c] *College of Pharmacy, Dali University, Dali, 671000, China*

[1]These authors contributed equally to this work

Correspondence to:

Shao Liu, PhD. Department of pharmacy, Xiangya Hospital, Central South University. No. 87 Xiang Ya Road Changsha 410008, People's Republic of China. E-mail: liushao999@csu.edu.cn

Or Yueping Jiang, PhD. Department of pharmacy, Xiangya Hospital, Central South University. No. 87 Xiang Ya Road Changsha 410008, People's Republic of China. E-mail: jiangyueping@csu.edu.cn

Supporting Materials

Table S1. Fifteen medicinal plants were screened for the treatment of insomnia by searching in Chinese Pharmacopoeia

TCM Name	Key small molecule types	References
Lilium lancifolium thumb	Steroidal saponins, Phenolic acids, Phenylpropanoids, Alkaloids, Flavonoids	40-42
Panax ginseng C.A mey	Saponins, Phytosterols, Flavonoids, Polyacetylenes, Fatty acids, Coumarins, Polyphenols, Phenolic acids, Cyclic dipeptides	43-44
Ziziphus jujuba mill. Var	Saponins, Alkaloids，Flavonoids	45-47
Bambusa tuldoids munro	Saponins, Lignans	48-49
Physochlaina infundibularis kuang	Alkaloids, Coumarins, Flavonoids, Phenolic acids	50-51
Albizia julibrissin durazz bark	Flavonoids, Triterpenoid saponins, Lignans, Phytosterols	52-53
Albizia julibrissin durazz flower	Flavonoids, Triterpenoid saponins	54
Polygala tenuifolia willd	Triterpenoid saponins, Xanthones	55-57
Ganoderma lucidum	Triterpenoid saponins, Alkaloids	59-62
Acanthopanax senticosus harms	Flavonoids, Triterpenoid saponins, Lignans, Coumarins	63-64
Apocynum venetum	Flavonoids, Organic acids, Phoroglucinols, Phytosterols, Other glycosides	65-67

Platycladus orientalis franco	Fatty acids, Triterpenoid saponins, Terpenes, Polyphenols, Flavonoids	68-70
Polygonum multiflorum thumb	Anthraquinones, Flavonoids, Phytosterols, Stilbenes、Anthocyanins	71-73
Nelumbo nucifera gaertn seeds	Alkaloids, Phenolic acids, Flavonoids	74-75
Plumula Nelumbinis	Alkaloids, Flavonoids, Phytosterols	76-77

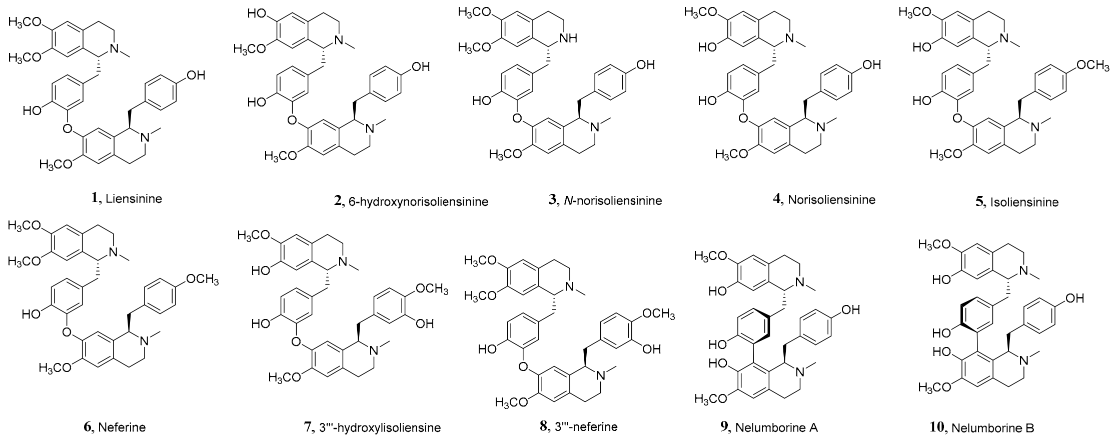

Figure S1. The structure of ten bisbenzylisoquinoline alkaloids

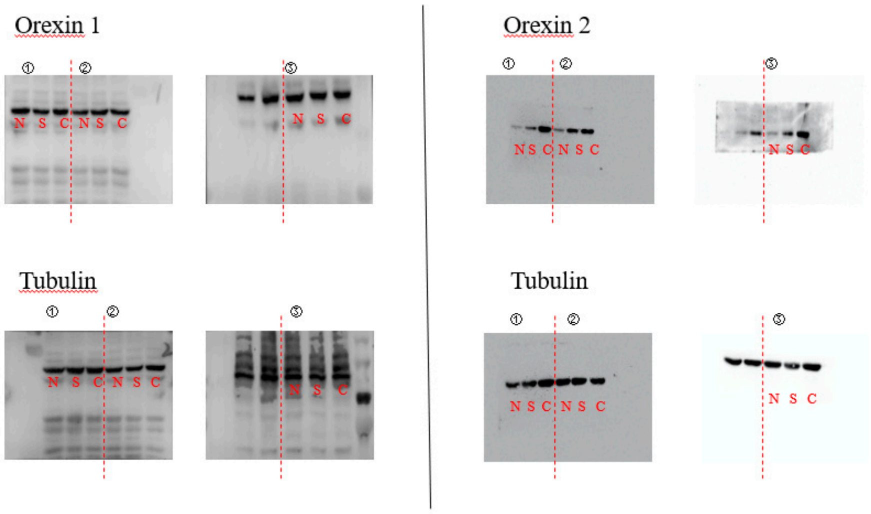

Figure S2. The original image of western bolts (C:Control; S:Suvorexant; N:Neferine)

Table S2 Chemical compositions in *Lilium lancifolium* Thumb

No.	Compound	Type	R
**1**	(25*S*)-spirost-5-ene-3*β*,27-diol 3-*O*-*β*-D-glucoside (lilioglycoside B)	S_1	$R_1 = G_1$, $R_2 = R_3 = H$
**2**	(25*S*)-spirost-5-en-3*β*,27-diol 3-*O*-[α-L-rha-(1→2)]-*β*-D-glucoside (deacylbrownioside/lilioglycoside E)	S_1	$R_1 = G_2$, $R_2 = R_3 = H$
**3**	27-*O*-(3-hydroxy-3-methylglutaroyl) isonarthogenin 3-*O*-α-L-rha-(1→2)-*O*-[*β*-D-glu-(1→4)]-*β*-D-glucoside	S_1	$R_1 = G_3$, $R_2 = H$, $R_3 =$ HMG
**4**	(25*S*)-27-hydroxyspirost-5-en-3*β*-yl *O*-α-L-rha-(1→2)-*O*-[*β*-D-glu-(1→6)]-*β*-D-glucoside	S_1	$R_1 = G_4$, $R_2 = R_3 = H$
**5**	(25*S*)-spirost-5-ene-3*β*,27-diol 3-*O*-α-L-rha-(1→2)-*O*-[α-L-ara-(1→3)]-*β*-D-glucoside	S_1	$R_1 = G_5$, $R_2 = R_3 = H$
**6**	(25*S*)-spirost-5-ene-3*β*,27-diol 3-*O*-α-L-rha-(1→2)-*O*-[*β*-D-glu-(1→4)]-*β*-D-glucoside	S_1	$R_1 = G_3$, $R_2 = R_3 = H$
**7**	(25*S*)-spirost-5-ene-3*β*,27-diol 3-*O*-α-L-rha-(1→2)-*O*-[*β*-D-glu-(1→3)]-*β*-D-glucoside (lilioglycoside H)	S_1	$R_1 = G_6$, $R_2 = R_3 = H$
**8**	(25*S*)-spirost-5-ene-3*β*,27-diol 3-*O*-*β*-D-glu-(1→3)-*O*-α-L-rha-(1→2)-*O*-[*β*-D-glu-(1→4)]-*β*-D-glucoside	S_1	$R_1 = G_7$, $R_2 = R_3 = H$
**9**	27-*O*-[(3*S*)-3-*O*-*β*-D-glu-3-methylglutaroyl] isonarthogenin 3-*O*-[α-L-rha-(1→2)]-*β*-D-glucoside	S_1	$R_1 = G_2$, $R_2 = H$, $R_3 =$ 3-*O*-glu-HMG
**10**	(25*S*)-spirost-5-ene-3*β*,17α,27-triol 3-*O*-*β*-D-glu-(1→2)-*O*-*β*-D-glu-(1→4)-*β*-D-glucoside	S_1	$R_1 = G_8$, $R_2 = OH$, $R_3 = H$
**11**	(25*S*)-spirost-5-ene-3*β*,17α,27-triol 3-*O*-α-L-rha-(1→2)-*O*-[*β*-D-glu-(1→2)-*O*-*β*-D-glu-(1→4)]-*β*-D-glucoside	S_1	$R_1 = G_9$, $R_2 = OH$, $R_3 = H$
**12**	(25*S*)-5α-spirostane-3*β*,17α,27-triol 3-*O*-*β*-D-glu-(1→2)-*O*-*β*-D-glu-(1→4)-*β*-D-glucoside	S_2	$R = G_8$
**13**	(24*S*,25*S*)-3*β*,17α,24-trihydroxy-5α-spirostan-6-one 3-*O*-α-L-rha-(1→2)]-*β*-D-glucoside	S_3	$R = G_2$
**14**	(25*R*)-spirost-5-en-3*β*-ol 3-*O*-*β*-D-glucoside (lilioglycoside A/trillin)	S_4	$R = G_1$

Table S2(continued)

No.	Compound	Type	R
**14**	(25*R*)-spirost-5-en-3*β*-ol 3-*O*-*β*-D-glucoside (glioglycoside A/trillin)	S_4	R = G_1
**15**	(25*R*)-spirost-5-en-3*β*-ol 3-*O*-L-rha-(1→2)-D-glucoside (lilioglycoside D/prosapogenin A of dioscin)	S_4	R = G_2
**16**	(25*R*)-spirost-5-en-3*β*-ol 3-*O*-α-L-rha-(1→2)-*O*-[*β*-D-glu-(1→3)]-*β*-D-glucoside (lilioglycoside G/gracillin)	S_4	R = G_6
**17**	(25*R*)-spirost-5-en-3*β*-ol 3-*O*-α-L-rha-(1→2)-*O*-[*β*-D-glu-(1→6)]-*β*-D-glucoside	S_4	R = G_4
**18**	(25*R*)-spirost-5-en-3*β*-ol 3-*O*-α-L-rha-(1→2)-*O*-[α-L-ara-(1→3)]-*β*-D-glucoside (lililancifoloside A)	S_4	R = G_5
**19**	dioscin	S_4	R = G_{10}
**20**	(25*R*)-spirost-5-ene-3*β*-ol 3-*O*-α-L-rha-(1→2)-*O*-[*β*-D-xyl-(1→3)]-*β*-D-glucoside (ophiopogonin D′)	S_4	R = G_{11}
**21**	diosgenin 3-*O*-*β*-D-glu-(1→2)-*β*-D-glu-(1→4)-*β*-D-galactoside (funkioside D)	S_4	R = G_{12}
**22**	diosgenin 3-*O*-[*β*-D-glu-(1→2)]-[*β*-D-xyl-(1→3)]-*β*-D-glu-(1→4)-*β*-D-galactoside (aspidistrin)	S_4	R = G_{13}
**23**	(25*R*)-2α,3*β*-dihydroxy-spirost-5-en-1*β*-*O*-α-L-rha-(1→2)-*β*-D-xyloside (atropuroside A)	S_5	R = G_{14}
**24**	(25*R*)-2α,3*β*,17α-trihydroxy-spirost-5-en-1*β*-*O*-*β*-D-xyloside (atropuroside C)	S_5	R = G_{15}
**25**	(25*R*)-spirost-5-en-3*β*,27-diol 3-*O*-α-L-rha-(1→2)-*β*-D-glucoside	S_6	$R_1 = G_2$, R_2 = H
**26**	(25*R*)-27-(3-hydroxy-3-methylglutarate)-spirost-5-ene-3*β*, 27-diol 3-*O*-*β*-D-glucoside (lilioglycoside C)	S_6	$R_1 = G_1$, R_2 = HMG
**27**	(25*R*)-27-*O*-(3-hydroxy-3-methylglutaroyl)-spirost-5-en-3*β*,27-diol 3-*O*-α-L-rha-(1→2)-*β*-D-glucoside (brownioside/lilioglycoside F)	S_6	$R_1 = G_2$, R_2 = HMG
**28**	(25*R*)-27-[(*S*)-3-hydroxy-3-methylglutarate)]-spirost-5-ene-3*β*, 27-diol 3-*O*-α-L-rha-(1→2)-*O*-[*β*-D-glu-(1→3)]-*β*-D-glucoside (lilioglycoside I)	S_6	$R_1 = G_6$, R_2 = HMG
**29**	(25*R*)-27-*O*-[(*S*)-3-hydroxy-3-methylglutaryl]-spirost-5-ene-3*β*,27-diol 3-*O*-α-L-rha-(1→2)-*O*-[*β*-D-glu-(1→4)]-*β*-D-glucoside	S_6	$R_1 = G_3$, R_2 = HMG
**30**	(25*R*)-27-*O*-[(*S*)-3-hydroxy-3-methylglutaryl]-spirost-5-ene-3*β*,27-diol 3-*O*-α-L-rha-(1→2)-*O*-[α-L-ara-(1→3)]-*β*-D-glucoside	S_6	$R_1 = G_5$, R_2 = HMG
**31**	(25*R*)-27-*O*-[(*S*)-3-hydroxy-3-methylglutaryl]-spirost-5α-3*β*,27-diol 3-*O*-α-L-rha-(1→2)-*β*-D-glucoside	S_7	$R_1 = G_2$, R_2 = HMG
**32**	(23*S*,25*R*)-23-hydroxyspirost-5-en-3*β*-*O*-α-L-rha-(1→2)-*O*-[*β*-D-glu-(1→6)]-*β*-D-glucoside	S_8	$R_1 = G_4$, $R_2 = R_4$ = H, R_3 = OH
**33**	(25*R*,26*R*)-17α-hydroxy-26-methoxyspirost-5-en-3*β*-*O*-α-L-rha-(1→2)-*O*-[*β*-D-glu-(1→6)]-*β*-D-glucoside	S_8	$R_1 = G_4$, R_2 = OH, R_3 = H, R_4 = OMe
**34**	(25*R*,26*R*)-26-methoxyspirost-5-en-3*β*-*O*-α-L-rha-(1→2)-*O*-[*β*-D-glu-(1→6)]-*β*-D-glucoside	S_8	$R_1 = G_4$, $R_2 = R_3$ = H, R_4 = OMe
**35**	(25*R*,26*R*)-26-methoxyspirost-5-en-3*β*-ol 3-*O*-α-L-rha-(1→2)-*O*-[α-L-ara-(1→3)]-*β*-D-glucoside	S_8	$R_1 = G_5$, $R_2 = R_3$ = H, R_4 = OMe
**36**	(25*R*,26*R*)-26-methoxyspirost-5-en-3*β*-ol 3-*O*-α-L-rha-(1→2)-*O*-[*β*-D-glu-(1→4)]-*β*-D-glucoside	S_8	$R_1 = G_3$, $R_2 = R_3$ = H, R_4 = OMe
**37**	(25*R*,26*R*)-26-methoxyspirost-5-en-3*β*-ol 3-*O*-α-L-rha-(1→2)-*β*-D-glucoside	S_8	$R_1 = G_2$, $R_2 = R_3$ = H, R_4 = OMe
**38**	(25*R*,26*R*)-26-methoxyspirost-5-ene-3*β*,17α-diol 3-*O*-α-L-rha-(1→2)-*β*-D-glucoside	S_8	$R_1 = G_2$, R_2 = OH, R_3 = H, R_4 = OMe
**39**	(25*R*,26*R*)-26-methoxyspirost-5-ene-3*β*,17α-diol 3-*O*-α-L-rha-(1→2)-*O*-[*β*-D-glu-(1→4)]-*β*-D-glucoside	S_8	$R_1 = G_3$, R_2 = OH, R_3 = H, R_4 = OMe

Table S2(continued)

No.	Compound	Type	R
**40**	(25*R*,26*R*)-26-methoxyspirost-5-ene-3β,17α-diol 3-*O*-α-L-rha-(1→2)-*O*-[6-*O*-acetyl-β-D-glu-(1→4)]-β-D-glucoside	S_8	R_1 = G_{16}, R_2 = OH, R_3 = H, R_4 = OMe
**41**	(25*R*,26*R*)-26-ethoxyspirost-5-ene-3β-ol 3-*O*-α-L-rha-(1→2)-[β-D-glu-(1→4)]-β-D-glucoside	S_8	R_1 = G_3, R_2 = R_3 = H, R_4 = OEt
**42**	(25*R*,26*R*)-26-methoxyspirost-5-ene-3β-ol 3-*O*-α-L-rha-(1→2)-*O*-[6-*O*-acetyl-β-D-glu-(1→4)]-β-D-glucoside	S_8	R_1 = G_{16}, R_2 = R_3 = H, R_4 = OMe
**43**	(25*R*)-spirost-5-en-3β,12α-diol 3-*O*-α-L-rha-(1→2)-*O*-[β-D-glu-(1→4)]-β-D-glucoside	S_9	R = G_3
**44**	(25*R*)-3β,17α-diol-5α-spirostan 3-*O*-α-L-rha-(1→2)-*O*-[β-D-glu-(1→4)]-β-D-glucoside	S_{10}	R_1 = G_3, R_2 = H
**45**	(25*R*)-5α-spirostan-3β,17α-diol 3-*O*-β-D-xyl-(1→4)-[α-L-ara-(1→6)]-β-D-glucoside	S_{10}	R_1 = G_{17}, R_2 = H
**46**	(25*R*)-spirost-3β,17α,21-triol 3-*O*-α-L-rha-(1→2)-[α-L-ara-(1→3)]-β-D-glucoside (pardarinoside E)	S_{10}	R_1 = G_5, R_2 = OH
**47**	(25*R*)-3β,17α-dihydroxy-5α-spirostan-6-one 3-*O*-α-L-rha-(1→2)-β-D-glucoside	S_{11}	R_1 = G_2, R_2 = OH, R_3 = H
**48**	(25*R*)-3β,17α-dihydroxy-5α-spirostan-6-one 3-*O*-α-L-rha-(1→2)-*O*-[α-L-ara-(1→3)]-β-D-glucoside	S_{11}	R_1 = G_5, R_2 = OH, R_3 = H
**49**	(25*R*)-3β-hydroxy-5α-spirost-6-one 3-*O*-α-L-rha-(1→2)-β-D-glucoside	S_{11}	R_1 = G_2, R_2 = R_3 = H
**50**	(25*R*)-3β-hydroxy-5α-spirostan-6-one 3-*O*-α-L-ara-(1→6)-β-D-glucoside	S_{11}	R_1 = G_{18}, R_2 = R_3 = H
**51**	(25*R*)-3β,17α,27-triol-spirostan-6-one 3-*O*-α-L-rha-(1→2)-β-D-glucoside (pumilum A)	S_{11}	R_1 = G_2, R_2 = R_3 = OH
**52**	(25*R*)-3β,17α,27-triol-spirostan-6-one 3-*O*-α-L-rha-(1→2)-[β-D-glu-(1→6)]-β-D-glucoside (callosum A)	S_{11}	R_1 = G_4, R_2 = R_3 = OH
**53**	26-*O*-β-D-glu-nuatigenin 3-*O*-α-L-rha-(1→2)-β-D-glucoside	S_{12}	R_1 = G_2, R_2 = G_1
**54**	26-*O*-β-D-glu-nuatigenin 3-*O*-α-L-rha-(1→2)-*O*-[β-D-glu-(1→4)]-β-D-glucoside	S_{12}	R_1 = G_3, R_2 = G_1
**55**	26-*O*-β-D-glu-nuatigenin	S_{12}	R_1 = H, R_2 = G_1
**56**	26-*O*-β-D-glu-nuatigenin 3-*O*-β-D-glucoside	S_{12}	R_1 = R_2 = G_1
**57**	26-*O*-β-D-glu-nuatigenin 3-*O*-α-L-rha-(1→2)-[β-D-glu-(1→6)]-β-D-glucoside	S_{12}	R_1 = G_4, R_2 = G_1
**58**	26-*O*-[β-D-glu-(1→2)]-β-D-glu-nuatigenin 3-*O*-α-L-rha-(1→2)-β-D-glucoside	S_{12}	R_1 = G_2, R_2 = G_{19}
**59**	(25*S*)-26-*O*-β-D-glu-22,25-epoxyfurost-5α-3β,26-diol 3-*O*-[α-L-rha-(1→2)]-β-D-glucoside	S_{13}	R_1 = G_2, R_2 = G_1
**60**	(25*R*)-furost-5-en-3β,22α,26-triol 3,26-di-*O*-β-D-glucoside (lilioglycoside K)	S_{14}	R_1 = R_3 = G_1, R_2 = H
**61**	(25*R*)-26-*O*-β-D-glu-furost-5-en-3β,22α,26-triol 3-*O*-L-rha-(1→2)-D-glucoside (lilioglycoside N)	S_{14}	R_1 = G_2, R_2 = H, R_3 = G_1
**62**	(25*R*)-26-*O*-β-D-glu-furost-5-en-3β,22α,26-triol 3-*O*-{[*O*-L-rha-(1→2)]-[*O*-D-glu-(1→3)]-β-D-glucoside} (lilioglycoside R)	S_{14}	R_1 = G_6, R_2 = H, R_3 = G_1
**63**	(25*R*)-22-*O*-methyl-26-*O*-β-D-glu-furost-5-ene-3β,22ξ,26-triol 3-*O*-α-L-rha-(1→2)-β-D-glucoside	S_{14}	R_1 = G_2, R_2 = H, R_3 = G_1
**64**	(25*R*)-22-*O*-methyl-26-*O*-β-D-glu-furost-5-ene-3β,22ξ,26-triol 3-*O*-α-L-rha-(1→2)-*O*-[β-D-glu-(1→3)-β-D-glucoside (trigofoenosideD-1)	S_{14}	R_1 = G_6, R_2 = Me, R_3 = G_1
**65**	(25*R*)-22-*O*-methyl-26-*O*-β-D-glu-furost-5-ene-3β,22ξ,26-triol 3-*O*-α-L-rha-(1→2)-*O*-[β-D-glu-(1→4)]-β-D-glucoside	S_{14}	R_1 = G_3, R_2 = Me, R_3 = G_1
**66**	(25*R*)-22-*O*-methyl-26-*O*-β-D-glu-furost-5-ene-3β,22ξ,26-triol 3-*O*-α-L-rha-(1→2)-*O*-[α-L-ara-(1→3)-β-D glucoside	S_{14}	R_1 = G_5, R_2 = Me, R_3 = G_1

Table S2(continued)

No.	Compound	Type	R
**67**	(25*R*)-22-*O*-methyl-26-*O*-(*β*-D-glu)-furost-5-ene-3*β*,22*ξ*,26-triol 3-*O*-α-L-rha-(1→2)-*O*-[*β*-D-xyl-(1→3)]-*β*-D-glucoside	S_{14}	R_1 = G_{11}, R_2 = Me, R_3 = G_1
**68**	(25*R*) 26-*O*-(*β*-D-glu)-22-methoxyfurost-5-en-3*β*-yl *O*-α-L-rha-(1→2)-*O*-[*β*-D-glu-(1→6)]-*β*-D-glucoside	S_{14}	R_1 = G_4, R_2 = Me, R_3 = G_1
**69**	(25*R*)-26-*O*-(*β*-D-glu)-furost-5-en-3*β*,22α,26-triol 3-*O*-α-L-rha-(1→2)-[α-L-ara-(1→3)]-*β*-D-glucoside	S_{14}	R_1 = G_5, R_2 = H, R_3 = G_1
**70**	(25*R*)-26-*O*-(*β*-D-glu)-furost-5-en-3*β*,22α,26-triol 3-*O*-α-L-rha-(1→2)-[α-L-xyl-(1→3)]-*β*-D-glucoside	S_{14}	R_1 = G_{11}, R_2 = H, R_3 = G_1
**71**	(25*R*)-26-*O*-(*β*-D-glu)-furost-5-en-3*β*,22*ξ*, 26-triol 3-*O*-α-L-rha-(1→2)-*O*-[*β*-D-glu-(1→4)]-*β*-D-glucoside	S_{14}	R_1 = G_3, R_2 = H, R_3 = G_1
**72**	(25*R*)-26-*O*-(*β*-D-glu)-22α-hydroxyfurost-5-en-3*β*-yl*O*-α-L-ara-(1→3)-*O*-[*β*-D-glu-(1→4)]-*O*-[α-L-rha-(1→2)]-*β*-D-glucoside	S_{14}	R_1 = G_{20}, R_2 = H, R_3 = G_1
**73**	(25*R*)-26-*O*-(*β*-D-glu)-22α-hydroxyfurost-5-en-3*β*-yl*O*-α-L-ara-(1→4)-*O*-[*β*-D-glu-(1→3)]-*O*-[α-L-rha-(1→2)]-*β*-D-glucoside	S_{14}	R_1 = G_{21}, R_2 = H, R_3 = G_1
**74**	(25*R*)-26-*O*-(*β*-D-glu)-furosta-5, 20(22)-dien-3*β*-yl*O*-α-L-ara-(1→3)-*O*-[*β*-D-glu-(1→4)]-*O*-[α-L-rha-(1→2)]-*β*-D-glucoside	S_{15}	R_1 = G_{20}, R_2 = G_1
**75**	(25*R*)-26-*O*-(*β*-D-glu)-furosta-5, 20(22)-dien-3*β*-yl*O*-α-L-ara-(1→4)-*O*-[*β*-D-glu-(1→3)]-*O*-[α-L-rha-(1→2)]-*β*-D-glucoside	S_{15}	R_1 = G_{21}, R_2 = G_1
**76**	(25*R*)-26-*O*-(*β*-D-glu)-5α-furostan-3*β*,22*ξ*-diol 3-*O*-α-L-rha-(1→2)-*O*-[*β*-D-glu-(1→4)]-*β*-D-glucoside	S_{16}	R_1 = G_3, R_2 = G_1
**77**	(25*R*)-22-*O*-methyl-26-*O*-acetyl-5α-furost-3*β*,14α,17α,22α,26-pentaol 3-*O*-α-L-rha-(1→2)-*β*-D-glucoside (pardarinoside A)	S_{17}	R_1 = G_2, R_2 = OH, R_3 = Ac
**78**	(25*R*)-22-*O*-methyl-26-*O*-acetyl-5α-furost-3*β*,17α,22α,26-tetraol 3-*O*-α-L-rha-(1→2)-*β*-D-glucoside (pardarinoside B)	S_{17}	R_1 = G_2, R_2 = H, R_3 = Ac
**79**	(25*R*)-22-*O*-methyl-26-*O*-acetyl-5α-furost-3*β*,14α,17α,22α,26-pentaol 3-*O*-α-L-rha-(1→2)-[*β*-D-glu-(1→4)]-*β*-D-glucoside (pardarinoside C)	S_{17}	R_1 = G_3, R_2 = OH, R_3 = Ac
**80**	(25*R*)-22-*O*-methyl-26-*O*-acetyl-5α-furost-3*β*,17α,22α,26-tetraol 3-*O*-α-L-rha-(1→2)-[*β*-D-glu-(1→4)]-*β*-D-glucoside (pardarinoside D)	S_{17}	R_1 = G_3, R_2 = H, R_3 = Ac
**81**	(25*R*)-22-*O*-methyl-26-*O*-acetyl-5α-furost-3*β*,14α,17α,22α,26-pentaol 3-*O*-α-L-rha-(1→2)-[α-L-ara-(1→3)]-*β*-D-glucoside (pardarinoside F)	S_{17}	R_1 = G_5, R_2 = OH, R_3 = Ac
**82**	(25*R*)-22-*O*-methyl-26-*O*-acetyl-5α-furost-3*β*,17α,22α,26-tetraol 3-*O*-α-L-rha-(1→2)-[α-L-ara-(1→3)]-*β*-D-glucoside (pardarinoside G)	S_{17}	R_1 = G_5, R_2 = H, R_3 = Ac
**83**	Regaloside F	S_{18}	R_1=OCH_3 ,R_2=3-O-β-D-Glu-glycerol
**84**	6' -O-feruloylsucrose	S_{18}	R_1=OCH_3 ,R_2=sucrose
**85**	3,6' -diferuloylsucrose	S_{18}	R_1=OCH_3 ,R_2=sucrose-6'-feruloyl
**86**	1-O-feruloylglycerol	S_{18}	R_1=OCH_3 ,R_2=CH_2-CH(OH)-CH_2(OH)
**87**	1-O-p-coumaroylglycerol	S_{18}	R_1=H ,R_2=CH_2-CH(OH)-CH_2(OH)
**88**	1-O-caffeoylglycerol	S_{18}	R_1=OH ,R_2=CH_2-CH(OH)-CH_2(OH)
**89**	1-O-caffeoyl-3-O-p-coumaroylglycerol	S_{19}	R_1=OH ,R_2=H
**90**	1,3-O-difeRuloylglycerol	S_{19}	R_1=OCH_3 ,R_2=OCH_3
**91**	1-O-feRuloyl-3-O-p-coumaRoylglyceRol	S_{19}	R_1=OCH_3 ,R_2=H
**92**	1,3-O-di-p-coumaroylglycerol	S_{19}	R_1=H ,R_2=H
**93**	1,2-O-diferuloylglycerol	S_{20}	R_1=OCH_3 ,R_2=OCH_3
**94**	1-O-feruloyl-2-O-p-coumaroylglycerol	S_{20}	R_1=OCH_3 ,R_2=H
**95**	1-O-p-coumaroyl-2-O-feruloylglycerol	S_{20}	R_1=H ,R_2=OCH_3
**96**	Regaloside A	S_{21}	R_1=H ,R_2=H ,R_3=β-D-Glu
**97**	Regaloside B	S_{21}	R_1=H ,R_2=β-D-Glu ,R_3=OCH_3
**98**	Regaloside D	S_{21}	R_1=H ,R_2=β-D-Glu ,R_3=H
**99**	Regaloside H	S_{21}	R_1=H ,R_2=H ,R_3=β-D-Glu
**100**	Regaloside C	S_{21}	R_1=OH ,R_2=H ,R_3=β-D-Glu
**101**	Regaloside E	S_{21}	R_1=OH ,R_2=β-D-Glu ,R_3=acetyl

Table S2(continued)

No.	Compound	Type	R
**102**	Solamargine	S_{22}	R = α-L-Rha-(1→2)-β-D-Glu
**103**	Khasianine	S_{22}	R = α-L-Rha-(1→2)-O-[β-D-Glu-(1→4)]-β-D-Glu
**104**	(22R,25R)-spirosol-5-en-3β-yl-O-α-L-Rha-(1→2)-6-O-acetyl-β-D-Glc-(1→4)-β-D- Glu	S_{22}	R=α-L-Rha-(1→2)-O-[6-O-acetyl-β-D-Glu-(1→4)]-β- D-Glu
**105**	Jatropham	S_{23}	R=H
**106**	Jatropham-5-O-β-D- Glu	S_{23}	R=β-D-Glu
**107**	Jatropham-5-O-[O-β-D- Glu -(1→3)]-β-D- Glu	S_{23}	R=β-D-Glu-(1→3)-β-D-Glu
**108**	Jatropham-5-O-(6-O-p-coumaroyl-β-D- Glu)	S_{23}	R=6-O-p-coumaroyl-β-D-Glu
**109**	Adenosine	S_{24}	R=OH
**110**	2'-deoxyadenosine	S_{24}	R=H
**111**	Colchichine	-	-
**112**	Berberine	-	-
**113**	Lilaline	-	-
**114**	Etioline	-	-
**115**	Rutin	S_{25}	R_1=rha*p* -(1→6)-glu*p*,R_2=H ,R_3=OH
**116**	Quercetin	S_{25}	R_1=H ,R_2=H ,R_3=OH
**117**	Dihydroquercetin	S_{26}	R_1=H ,R_2=H ,R_3=H
**118**	Kaempferol	S_{25}	R_1=H ,R_2=OH ,R_3=OH
**119**	Myricetin	S_{25}	R_1=OH ,R_2=H
**120**	Dihydromyricetin	S_{26}	R_1=OH ,R_2=OH
**121**	Eriodictyol	S_{26}	R_1=H ,R_2=H
**122**	Catechin	-	-
**123**	Epi-catechin	-	-
**124**	Cyanidin 3- rutinoside	-	-
**125**	Phloridzin	-	-

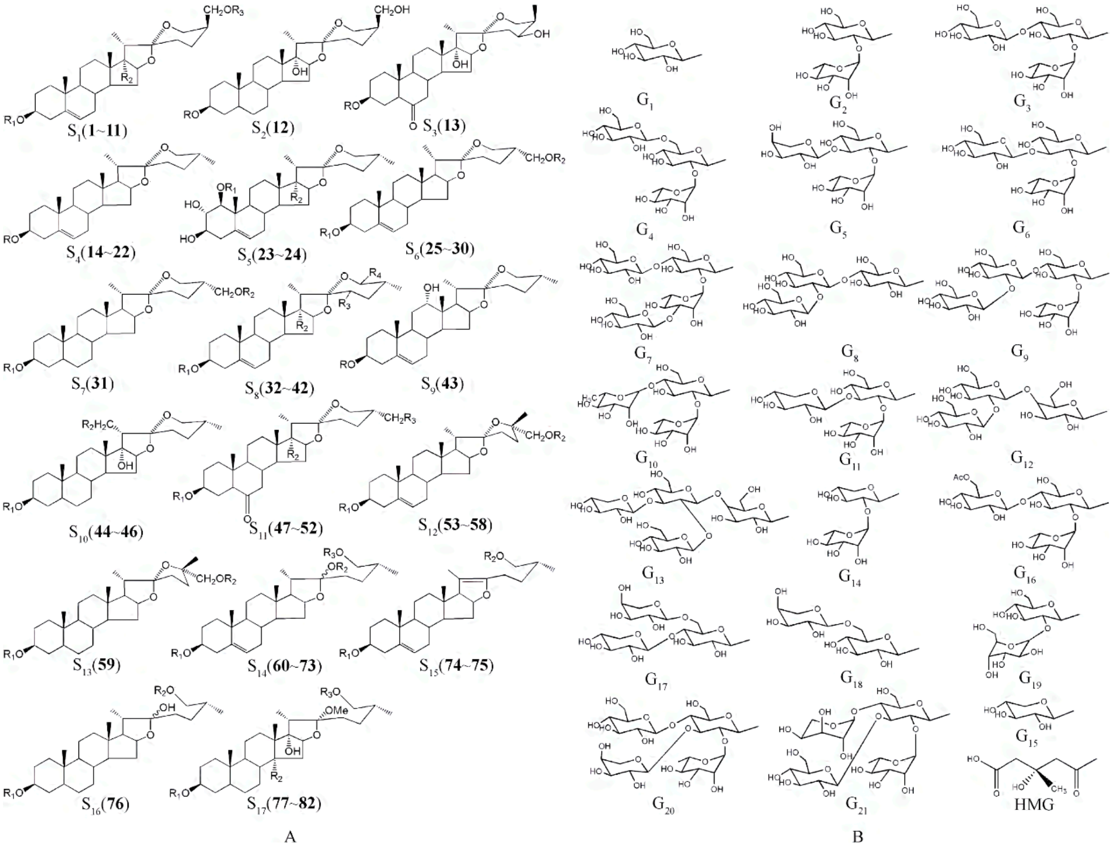

Figure S3. The structure of compounds in *Lilium lancifolium* Thumb

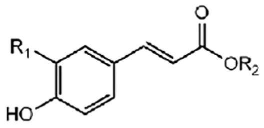

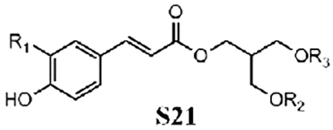

S21

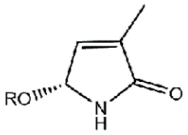

S23

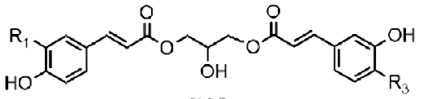

S19

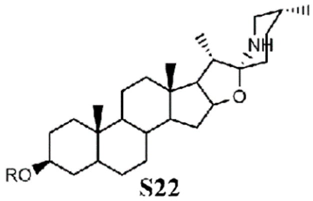

S22

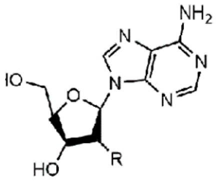

S24

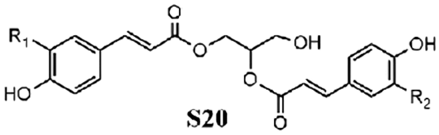

S20

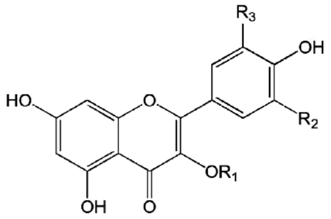

S25

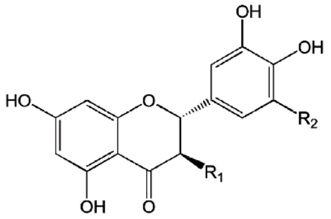

S26

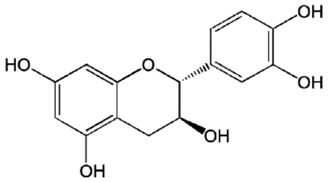

Catechin

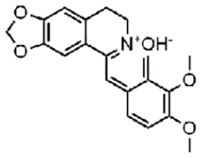

Berberine

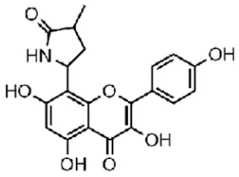

Lilaline

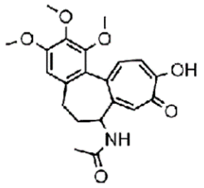

Colchichine

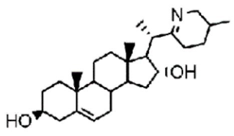

Etioline

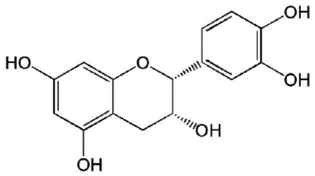

Epi-catechin

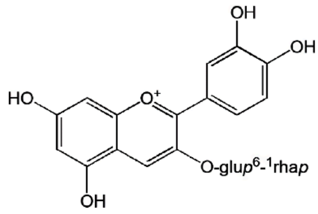

Cyanidin 3-rutinside

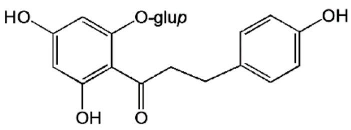

Phloridzin

Figure S3. (continued)

Table S3 Chemical compositions in *Panax ginseng* C.A mey (Part.1)

NO.	Compound	R_1	R_2	R_3	R_4	C_{20}
1	20(*S*)-25-OCH_3-PPD	H	OH	OH	OCH_3	*S*
2	20(*R*)-Protopanaxadiol	H	OH	OH	CH_3	*S*
3	20(*S*)- Protopanaxadiol	H	OH	CH_3	OH	*R*
4	Ginsenoside Ra$_1$	Glc^2-Glc	OH	*O*-Glc^6-Ara(*p*)^4-Xyl	CH_3	*S*
5	Ginsenoside Ra$_2$	Glc^2-Glc	OH	*O*-Glc^6-Ara(*f*)^2-Xyl	CH_3	*S*
6	Ginsenoside Ra$_3$	Glc^2-Glc	OH	*O*-Glc^6-Glc^3-Xyl	CH_3	*S*
7	Ginsenosides Ra$_4$	S5	OH	*O*-Glc^6-Ara(*p*)^4-Xyl	CH_3	*S*
8	Ginsenosides Ra$_5$	S6	OH	*O*-Glc^6-Ara(*p*)^4-Xyl	CH_3	*S*
9	Ginsenosides Ra$_6$	S5	OH	*O*-Glc^6-Glc	CH_3	*S*
10	Ginsenosides Ra$_7$	S5	OH	$OGlc^6$-Ara(*f*)	CH_3	*S*
11	Ginsenosides Ra$_8$	S7	OH	$OGlc^6$-Ara(*f*)	CH_3	*S*
12	Ginsenosides Ra$_9$	S5	OH	$OGlc^6$-Ara(*f*)	CH_3	*S*
13	Ginsenoside Rb$_1$	Glc^2-Glc	OH	*O*-Glc^6-Glc	CH_3	*S*
14	Ginsenoside Rb$_2$	Glc^2-Glc	OH	*O*-Glc^6-Ara(*p*)	CH_3	*S*
15	Ginsenoside Rb$_3$	Glc^2-Glc	OH	*O*-Glc^6-Xyl	CH_3	*S*
16	Ginsenoside Rc	Glc^2-Glc	OH	*O*-Glc^6-Ara(*f*)	CH_3	*S*
17	Ginsenoside Rd	Glc^2-Glc	OH	*O*-Glc	CH_3	*S*
18	20(*S*)-Ginsenoside Rg$_3$	Glc^2-Glc	OH	OH	CH_3	*S*
19	20(*R*)-Ginsenoside Rg$_3$	Glc^2-Glc	OH	CH_3	OH	*R*
20	20(*S*)-ginsenoside Rh$_2$	Glc	OH	OH	CH_3	*S*
21	20(*R*)-ginsenoside Rh$_2$	Glc	OH	CH_3	OH	*R*
22	Ginsenoside F$_2$	Glc	OH	*O*-Glc	CH_3	*S*
23	Ginsenoside Mc	H	OH	*O*-Glc^6-Ara(*f*)	CH_3	*S*

Table S3(continued)

NO.	Compound	R_1	R_2	R_3	R_4	C_{20}
22	Ginsenoside F_2	Glc	OH	*O*-Glc	CH_3	*S*
23	Ginsenoside Mc	H	OH	*O*-Glc^{6}-Ara(*f*)	CH_3	*S*
24	Ginsenoside Rs_2	Glc^{2}-Glc^{6}-AC	OH	*O*-Glc^{2}-Ara(*f*)	CH_3	*S*
25	Ginsenoside Rs_3	Glc^{2}-Glc-(6-*O*-AC)	OH	OH	CH_3	*S*
26	6″-Acetyl-ginsenoside-Rd	Glc^{2}-Glc^{6}-$COCH_3$	OH	*O*-Glc	CH_3	*S*
27	Malonyl ginsenoside Ra_3	Glc^{2}-Glc-COCOCH2O	OH	*O*-Glc^{6}-Glc^{3}-Xyl	CH_3	*S*
28	Malonyl ginsenoside Rb_1	Glc^{2}-Glc-(6-*O*-Mal)	OH	*O*-Glc^{6}-Glc	CH_3	*S*
29	Malonyl ginsenoside Rb_2	Glc^{2}-Glc-(6-*O*-Mal)	OH	*O*-Glc^{6}-Ara(*p*)	CH_3	*S*
30	Malonyl ginsenoside Rc	Glc^{2}-Glc-(6-*O*-Mal)	OH	*O*-Glc^{6}-Ara(*f*)	CH_3	*S*
31	Malonyl ginsenoside Rd	Glc^{2}-Glc-(6-*O*-Mal)	OH	*O*-Glc	CH_3	*S*
32	Malonyl notoginsenoside R_4	Glc^{2}-(6-Mal)Glc	OH	*O*-Glc^{6}-Glc^{6}-Xyl	CH_3	*S*
33	Notoginsenoside Fa	Glc^{2}-Glc^{2}-Xyl	OH	*O*-Glc^{6}-Glc	CH_3	*S*
34	Notoginsenoside Fc	Glc^{2}-Glc^{2}-Xyl	OH	*O*-Glc^{6}-Xyl	CH_3	*S*
35	Notoginsenoside Fe	Glc	OH	*O*-Glc^{6}-Ara(*p*)	CH_3	*S*
36	Notoginsenoside Ft_1	Glc^{2}-Glc^{2}-Xyl	OH	OH	CH_3	*S*
37	Notoginsenoside FP_2	Glc^{2}-Glc^{2}-Xyl	OH	*O*-Glc^{6}-Ara(*p*)	CH_3	*S*
38	Notoginsenoside D	Glc^{2}-Glc^{2}-Xyl	OH	*O*-Glc^{6}-Glc^{6}-Xyl	CH_3	*S*
39	Notoginsenoside K	Glc^{6}-Glc	OH	*O*-Glc	CH_3	*S*
40	Notoginsenoside L	Glc^{2}-Xyl	OH	*O*-Glc^{6}-Glc	CH_3	*S*
41	Notoginsenoside O	Glc	OH	*O*-Glc^{6}-Xyl^{3}-Xyl	CH_3	*S*
42	Notoginsenoside P	Glc	OH	*O*-Glc^{6}-Xyl^{4}-Xyl	CH_3	*S*
43	Notoginsenoside Q	Glc^{2}-Glc^{2}-Xyl	OH	*O*-Glc^{6}-Xyl^{4}-Xyl	CH_3	*S*
44	Notoginsenoside S	Glc^{2}-Glc^{2}-Xyl	OH	*O*-Glc^{6}-Ara(*f*)^{5}-Xyl	CH_3	*S*
45	Notoginsenoside T	Glc^{2}-Glc^{2}-Xyl	OH	*O*-Glc^{6}-Glc^{3}-Xyl	CH_3	*S*
46	Notoginsenoside R_4	Glc^{2}-Glc	OH	*O*-Glc^{6}-Glc^{6}-Xyl	CH_3	*S*
47	Notoginsenoside ST_4	Glc^{2}-Glc^{2}-Xyl	OH	OH	CH_3	*S*
48	Notoginsenoside FZ	Glc^{2}-Glc^{2}-Xyl	OH	*O*-Glc^{6}-Ara(p)	CH_3	*S*
49	Notoginsenoside Fh_1	Glc^{2}-Glc^{2}-Xyl	OH	*O*-Glc^{6}-Ara(*p*)^{4}-Xyl	CH_3	*S*
50	Notoginsenoside L_5	S4	OH	*O*-Glc^{6}-Ara(*f*)	CH_3	*S*
51	Notoginsenoside L_6	S4	OH	*O*-Glc^{6}-Ara(*p*)	CH_3	*S*
52	Notoginsenoside L_7	S4	OH	*O*-Glc^{6}-Xyl	CH_3	*S*
53	Notoginsenoside L_8	S4	OH	*O*-Glc^{6}-Glc	CH_3	*S*
54	Gypenoside IX	Glc	OH	*O*-Glc^{6}-Xyl	CH_3	*S*
55	Gypenoside V	Glc^{2}-Glc	OH	*O*-Glc^{6}-Rha	CH_3	*S*
56	Gypenoside XVII	Glc	OH	*O*-Glc^{6}-Glc	CH_3	*S*
57	Gypenoside XIII	H	OH	*O*-Glc^{6}-Xyl	CH_3	*S*
58	Chikusetsusaponin VI	Glc-Xyl^{6}-Xyl	OH	*O*-Glc-Glc^{6}	CH_3	*S*
59	Chikusetsusaponin III	Glc-Glc^{6}-Xyl	OH	OH	CH_3	*S*
60	Chikusetsusaponin VII	Glc^{6}-Xyl	OH	*O*-Glc^{6}-Glc	CH_3	*S*
61	Chikusetsusaponin FK_4	Xyl-^{6}Glc^{2}-Glc	OH	*O*-Glc^{6}-Ara(*f*)	CH_3	*S*
62	Chikusetsusaponin FK_5	Xyl-^{6}Glc^{2}-Glc	OH	*O*-Glc^{6}-Xyl	CH_3	*S*
63	Chikusetsusaponin FK_6	Xyl-^{6}Glc^{2}-Glc	OH	*O*-Glc	CH_3	*S*
64	Chikusetsusaponin FK_7	Glc^{2}-Glc	*O*-Glc	OH	CH_3	*S*
65	Quinquenoside I	S1	OH	*O*-Glc	CH_3	*S*
66	Quinquenoside II	S2	OH	*O*-Glc^{6}-Glc	CH_3	*S*
67	Quinquenoside III	S3	OH	*O*-Glc	CH_3	*S*
68	Quinquenoside V	Glc^{2}-Glc	OH	*O*-Glc^{6}-Glc^{4}-Glc	CH_3	*S*
69	Quinquenoside L_2	Glc^{2}-Glc	OH	*O*-Glc	CH_3	*S*
70	Quinquenosides L_{10}	Glc	OH	*O*-Glc^{6}-Ara(*p*)	CH_3	*S*
71	Quinquenosides L_{14}	Glc^{2}-Glc	OH	*O*-Ara(*p*)	CH_3	*S*
72	Quinquenosides L_{16}	Glc^{2}-Glc	OH	*O*-Glc^{6}-Glc	CH_3	*S*
73	Compound O	Glc	OH	*O*-Glc^{2}-Ara(*p*)	CH_3	*S*
74	Compound K	H	OH	*O*-Glc	CH_3	*S*
75	Compound Y	H	OH	OH	CH_3	*S*
76	Pseu-ginsenoside Rc_1	Glc^{2}-Glc^{6}-AC	OH	*O*-Glc	CH_3	*S*
77	Yesanchinoside J	Glc^{2}-Glc^{6}-AC	OH	*O*-Glc^{6}-Glc^{6}-Xyl	CH_3	*S*
78	Vinaginsenoside R_{16}	Glc^{2}-Xyl	*O*-Glc	OH	CH_3	*S*
79	Vinaginsenoside R_3	Glc^{2}-Glc	H	*O*-Glc	CH_3	*S*
80	Quinquenoside Jb	Glc^{2}-Glc	OH	*O*-Glc^{6}-Glc^{6}-Ara(*f*)	CH_3	*S*
81	20(*R*)methoxyl-ginsenoside Rg_3	Glc^{2}-Glc	OH	OCH_3	CH_3	*R*
82	20(*S*)methoxyl-ginsenoside Rg_3	Glc^{2}-Glc	OH	OCH_3	CH_3	*S*
83	Malonyl-floralginsenosides Rb_1	Glc^{2}-Glc	H	*O*-Glc^{6}-(4-Mal)Glc	CH_3	*S*
84	Malonyl-floralginsenosides Rb_2	Glc^{2}-(3-Mal)Glc	H	*O*-Glc^{6}-Glc	CH_3	*S*
85	Malonyl-floralginsenosides Rd_1	Glc^{2}-(2-Mal)Glc	H	*O*-Glc	CH_3	*S*
86	Malonyl-floralginsenosides Rd_2	Glc^{2}-(3-Mal)Glc	H	*O*-Glc	CH_3	*S*
87	Malonyl-floralginsenosides Rd_3	Glc^{2}-(4-Mal)Glc	H	*O*-Glc	CH_3	*S*
88	Malonyl-floralginsenosides Rd_4	Glc^{2}-Glc	H	*O*-(3-Mal)Glc	CH_3	*S*
89	Malonyl-floralginsenosides Rd_5	Glc^{2}-Glc	H	*O*-(6-Mal)Glc	CH_3	*S*
90	Malonyl-floralginsenosides Rd_6	Glc^{2}-(6-Mal)Glc	H	*O*-(6-Mal)Glc	CH_3	*S*
91	Malonyl-floralginsenosides Rc_1	Glc^{2}-(6-Mal)Glc	H	*O*-Glc^{6}-Xyl	CH_3	*S*
92	Malonyl-floralginsenosides Rc_2	Glc^{2}-(4-Mal)Glc	H	*O*-Glc^{6}-Ara(*p*)	CH_3	*S*
93	Malonyl-floralginsenosides Rc_3	Glc^{2}-(3-Mal)Glc	H	*O*-Glc^{6}-Ara(*p*)	CH_3	*S*
94	Malonyl-floralginsenosides Rc_4	Glc^{2}-(3-Mal)Glc	H	*O*-Glc^{6}-Ara(*f*)	CH_3	*S*

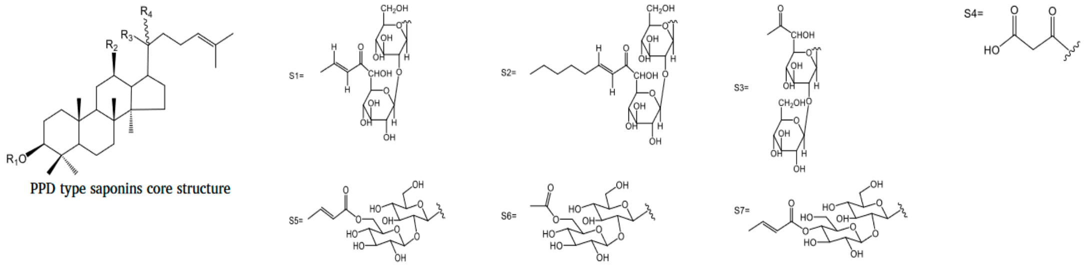

Figure S4. The structure of compounds in *Panax ginseng* C.A mey(Part.1)

Table S4 Chemical compositions in *Panax ginseng* C.A mey (Part.2)

NO.	Compound	R_1	R_2	R_3	R_4	R_5
95	20(*S*)- protopanaxatriol	H	H	H	OH	CH_3
96	20(*R*)-protopanaxatriol	H	H	H	CH_3	OH
97	Ginsenoside Rg$_1$	H	Glc	H	*O*-Glc	CH_3
98	20(*S*)-ginsenoside Rg$_2$	H	Glc^6-Rha	H	OH	CH_3
99	20(*R*)-ginsenoside Rg$_2$	H	Glc^6-Rha	H	CH_3	OH
100	20(*S*)-ginsenoside Rh$_1$	H	Glc	H	OH	CH_3
101	20(*R*)-ginsenoside Rh$_1$	H	Glc	H	CH_3	OH
102	Ginsenoside Rf	H	Glc^2-Glc	H	OH	CH_3
103	Ginsenoside F$_1$	H	H	H	*O*-Glc	CH_3
104	Ginsenoside Re	H	Glc^2-Rha	H	*O*-Glc	CH_3
105	Ginsenoside Re$_1$	H	Glc	H	*O*-Glc^3-Glc	CH_3
106	Ginsenoside Re$_2$	H	Glc^3-Glc	H	*O*-Glc	CH_3
107	Ginsenoside Re$_3$	H	Glc	H	*O*-Glc^4-Glc	CH_3
108	Ginsenoside Re$_4$	H	Glc	H	*O*-Glc^6-Ara(*f*)	CH_3
109	Ginsenoside F$_3$	H	H	H	*O*-Glc^6-Ara(*p*)	CH_3
110	Ginsenoside F$_5$	H	H	H	*O*-Glc^6-Ara(*f*)	CH_3
111	Ginsenoside Rh$_{24}$	H	Ara(*p*)^6-Rha	H	*O*-Glc	CH_3
112	Ginsenoside Rg$_{18}$	H	Glc	H	*O*-Glc^2-Rha	CH_3
113	Ginsenoside Rs$_{11}$	H	Glc^2-*O*Glc^6-AC	H	*O*-Glc^6-Ara(*f*)	CH_3
114	Ginsenoside Rh$_{25}$	*O*-Xyl-Glc^2	H	H	*O*-Ara-Glc^6	CH_3
115	Ginsenoside Rh$_{26}$	*O*-(*E*)-but-2-enoyl-Glc-Glc^2	H	H	*O*-Glc	CH_3
116	Ginsenoside Re$_5$	H	Glc^2-Glc	H	OH	CH_3
117	Ginsenoside Mb	Glc	H	H	*O*-Glc^6-Ara(*p*)	CH_3
118	20(*R*)-ginsenoside Rh$_{19}$	Glc	H	H	CH_3	OH
119	Notoginsenoside R$_1$	H	Glc^2-Xyl	H	*O*-Glc	CH_3
120	20(*R*)-Notoginsenoside R$_2$	H	Glc^2-Xyl	H	CH_3	OH
121	Notoginsenoside R$_3$	H	Glc	H	*O*-Glc^6-Glc	CH_3
122	Notoginsenoside R$_6$	H	Glc	H	*O*-Glc^6-Glc*	CH_3
123	Notoginsenoside N	H	Glc^4-Glc*	H	*O*-Glc	CH_3
124	Notoginsenoside Rt	H	Glc^6-OAC	H	*O*-Glc	CH_3
125	Notoginsenoside FP$_1$	H	Glc	H	*O*-Glc^6-Ara(*p*)	CH_3
126	Notoginsenoside Rw$_1$	H	Xyl	H	*O*-Glc^6-Xyl	CH_3
127	Notoginsenoside Fh$_7$	H	Glc^2-Glc	H	*O*-Glc^6-Glc	CH_3
128	Notoginsenoside M	H	Glc^6-Glc*	H	*O*-Glc	CH_3
129	Notoginsenoside L$_4$	S1′	H	H	*O*-Glc	OH
130	Notoginsenoside L$_9$	S4	H	H	*O*-Glc^6-Ara(*f*)	CH_3
131	Notoginsenoside L$_{10}$	S4	H	H	*O*-Glc^6-Ara(*p*)	CH_3
132	Notoginsenoside L$_{11}$	S4	H	H	*O*-Glc^6-Xyl	CH_3
133	Notoginsenoside L$_{12}$	S4	H	H	*O*-Glc^6-Glc	CH_3
134	Chikusetsusaponin LM$_1$	H	H	H	*O*-Glc^6-Xyl	CH_3
135	Chikusetsusaponin LM$_2$	H	H	H	*O*-Glc^6-Xyl^3-Xyl	CH_3
136	Chikusetsusaponin LM$_3$	H	H	H	*O*-Glc^6-Ara(*p*)^4-Xyl	CH_3
137	Chikusetsusaponin LM$_4$	Glc^2-Glc	H	Glc	OH	CH_3
138	Chikusetsusaponin LM$_5$	Glc^2-Glc	H	H	*O*-Glc^6-Ara(*f*)	CH_3
139	Chikusetsusaponin LM$_6$	Glc^2-Glc	H	H	*O*-Glc^6-Ara(*p*)^4-Ara(*f*)	CH_3
140	Chikusetsusaponin FK$_1$	Glc^2-Rha	Glc	H	CH_3	CH_3
141	Chikusetsusaponin L$_{10}$	H	H	Glc	OH	CH_3
142	Chikusetsusaponin L$_5$	H	H	H	*O*-Glc^6-Ara(*p*)^4-Xyl	CH_3
143	3-acetyl ginsenoside F$_1$	AC	H	H	*O*-Glc	CH_3
144	6′-acetyl-ginsenoside F$_1$	H	H	H	*O*-Glc^6-AC	CH_3
145	3β-acetoxyl ginsenoside F$_1$	COO	H	H	*O*-Glc	CH_3
146	6′-acetyl ginsenoside Rg$_3$	H	Glc^2-Glc^2	H	O-Glc^6-AC	CH_3
147	20-*O*-Gluco-ginsenoside R$_f$	H	Glc^2-Glc	H	*O*-Glc	CH_3
148	6′-malonyl formyl-ginsenoside F$_1$	H	H	H	*O*-Glc	CH_3
149	6′-emalonyl formyl ginsenoside F$_1$	H	H	H	S2′	CH_3
150	Pseudoginsenoside RT$_3$	H	Xvl	H	*O*-Glc	CH_3
151	Pseudo-ginsenosides F$_8$	AC-^{6}Glc^2-Glc	H	H	*O*-Glc^6-Xyl	CH_3
152	Pseudoginsenoside Rs$_1$	AC-^{6}Glc^2-Rha	H	H	*O*–CH_3	CH_3
153	Floralquinquenosides E	H	Glc^2-Rha	H	*O*-Glc^6-Xyl	CH_3
154	Floralginsenoside M	H	Glc^2-Rha	H	*O*-Glc^6-Ara(*f*)	CH_3
155	Floralginsenoside N	H	Glc^2-Rha	H	*O*-Glc^6-Ara(*p*)	CH_3
156	Floralginsenoside P	Glc^2-Glc	H	H	*O*-Glc^6-Ara(*p*)	CH_3
157	Quinquenoside L$_{17}$	H	Glc	H	*O*-Glc^6-Xyl	CH_3
158	Quinquenoside R$_1$	Glc^2-Glc^6-AC	H	H	*O*-Glc^6-Glc	CH_3
159	Koryoginsenoside R$_1$	H	Glc^6-Bu	H	*O*-Glc	CH_3
160	20(*S*)-sanchirhinosides A$_1$	H	S3′	H	OH	CH_3
161	20(*S*)-sanchirhinosides A$_2$	H	S4′	H	OH	CH_3
162	20(*S*)-sanchirhinosides A$_3$	H	Glc	H	*O*-Ara(*p*)	CH_3
163	20(*S*)-sanchirhinosides A$_4$	H	Ara(*p*)	H	*O*-Glc	CH_3
164	20(*S*)-sanchirhinosides A$_5$	H	Glc^2-Ara(*f*)	H	*O*-Glc	CH_3
165	20(*S*)-sanchirhinosides A$_6$	H	Glc^2-Xyl	H	*O*-Glc^6-Glc	CH_3
166	6α-acetoxy-3β,12β,20R-trihydroxydammar-24-ene	H	OAC	H	CH_3	OH
167	3-*O*-β-D-glucopyranosyl-20(*S*)-protopanaxatriol	Glc	H	H	OH	CH_3
168	3-formyloxy-20-*O*-β-D-glucopyranosyl-20(*S*)-protopanaxatriol	S5′	H	H	*O*-Glc	CH_3
169	Vina-ginsenosides R$_4$	Glc^4-Glc	H	H	*O*-Glc	CH_3
179	Vina-ginsenosides R$_7$	Glc^2-Glc-Xyl	H	H	*O*-Glc	CH_3
171	Yesanchinoside D	H	Glc^6-AC	H	*O*-Glc	CH_3
172	Yesanchinoside E	Glc^2-Rha	H	H	CH_3	*O*-Glc^6-Xyl
173	20(*R*)-ginsenoside Rh$_5$	OH	*O*-Glc	H	CH_3	*O*–CH_3
174	6-*O*-[β-D-glucopyranosyl-(1 → 2)-β-D–glucopyranosyl]-20-*O*-[β-D-glucopyranosyl-(1 → 4)-β-D-glucopyranosyl]-20(*S*)-protopanaxatriol	H	Glc^2-Glc	H	*O*-Glc^4-Glc	CH_3
175	20(*S*)-6-*O*-[β-D-xylopyranosyl-(1 → 2)-β-D-xylopyranosyl]dammar-24-ene-3β,6α,12β,20-tetrol	Xyl-Xyl^2	H	H	OH	CH_3
176	Malonyl-ginsenoside Rg$_1$	H	Glc^6-Mal	H	*O*-Glc	CH_3
177	Malonyl-ginsenoside Re	H	Glc^2-Rha-Mal	H	*O*-Glc	CH_3
178	Malonyl-floralginsenosides Re$_1$	H	(6-Mal)Glc^2-Rha	H	*O*-Glc	CH_3
179	Malonyl-floralginsenosides Re$_2$	H	Glc^2-Rha	H	*O*-(2-Mal)Glc	CH_3
180	Malonyl-floralginsenosides Re$_3$	H	Glc^2-Rha	H	*O*-(4-Mal)Glc	CH_3
181	20(*R*)-ginsenoside Rh$_1$ 6′-acetate	H	Glc^6-Ac	H	CH_3	OH
182	20(*S*)-ginsenoside Rh$_1$ 6′-acetate	H	Glc^6-Ac	H	OH	CH_3
183	(20S)-20-*O*-[β-D-xylopyranosyl-(1 → 6)-β-D-glucopyranosyl-(1 → 6)-β-D-glucopyranosyl] dammar-24-ene-3β,6α,12β,20-tetrol	H	H	H	*O*-Xyl-Glc^6-Glc^6	CH_3
184	(20S)-6-*O*-[(*E*)-but-2-enoyl-(1 → 6)-β-D-glucopyranosyl] dammar-24-ene-3β,6α,12β,20-tetrol	H	S3′	H	OH	CH_3
185	Ginsenoside la	Glc	H	H	*O*-Glc	CH_3
186	Pseudo-ginsenoside RT$_8$	Glc^2-Glc	H	H	CH_3	CH_3
187	Quinquenoside Ja	H	Glc^2-Glc	H	*O*-Glc^4-Glc	CH_3

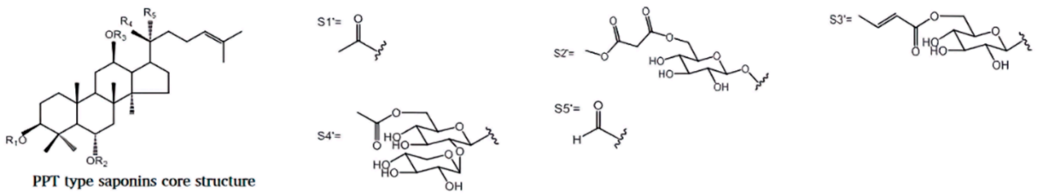

Figure S5. The structure of compounds in *Panax ginseng* C.A mey (Part.2)

Table S5 Chemical compositions in *Panax ginseng* C.A mey (Part.3)

NO.	Compound	R_1	R_2	R_3
188	Taibaienoside IV	Glc-UA²-Glc	CH_3	H
189	Calenduloside E	Glc-UA	CH_3	H
190	Oleanolic acid 3-*O*-[*β*-D-glucopyranosyl-(1 → 2)–*β*-D-glucuronopyranosyl-6′-O-n-butyl ester]	I2	CH_3	H
191	Calenduloside B	Glc⁴-Gal	CH_3	Glc
192	Pjs-1 (oleanolic acid 28-*O*-*β*-D-glucopyranoside)	H	CH_3	Glc
193	Pseudo-ginsenoside-RI_3	I3	Xyl	H
194	Pseudoginsenoside RP_1	GlcUA²-Xyl	CH_3	H
195	Polyacetyleneginsenoside Ro	I1	CH_3	Glc
196	28-desglucosyl chikusetsusaponin IV	GlcUA⁴-Ara(*p*)	CH_3	H
197	Oleanolic acid	H	CH_3	H

NO.	Compound	R_1 = I4 r_1	r_2	r_3	r_4	R_2	R_3
198	Bifinoside A	Ara(*p*)	H	H	$COOCH_3$	CH_3	H
199	Bifinoside B	H	Xyl⁶-Glc	H	$COOCH_3$	CH_3	H
200	Bifinoside C	Xyl	Ara(*p*)	H	$COOCH_3$	CH_3	Glc
201	Taibaienoside I	H	H	Ara(*f*)	n-Bu	CH_3	Glc
202	Chikusetsusaponin IV	H	H	Ara(*f*)	COOH	CH_3	Glc
203	Chikusetsusaponin IV methyl ester	H	H	Ara(*f*)	$COOCH_3$	CH_3	Glc
204	Chikusetsusaponin IV*a*	H	H	H	COOH	CH_3	Glc
205	Chikusetsusaponin IVa methyl ester	H	H	H	$COOCH_3$	CH_3	Glc
206	Chikusetsusaponin IVa butyl ester	H	H	H	n-Bu	CH_3	Glc
207	Chikusetsusaponin V	H	H	Glc	COOH	CH_3	Glc
208	Chikusetsusaponin Ib	Ara(*f*)	H	H	COOH	CH_3	Glc
209	Stipuleanoside R_1	H	Glc	Ara(*f*)	COOH	CH_3	H
210	Stipuleanoside R_2	H	Glc	Ara(*f*)	COOH	CH_3	Glc
211	Stipuleanoside R_2 methyl ester	H	Glc	Ara(*f*)	$COOCH_3$	CH_3	Glc
212	Pseudoginsenoside RT_1	Xyl	H	H	COOH	CH_3	Glc
213	Pseudoginsenoside RT_1 methyl ester	Xyl	H	H	$COOCH_3$	CH_3	Glc
214	Pseudoginsenoside Rp_1 methyl ester	Xyl	H	H	$COOCH_3$	CH_3	H
215	Ginsenoside Ro	Glc	H	H	COOH	CH_3	H
216	Ginsenoside Ro methyl ester	Glc	H	H	$COOCH_3$	CH_3	Glc
217	Spinasaponin A 28-*O*-glucoside	H	Glc	H	COOH	CH_3	H
218	Araloside A methyl ester	H	H	Ara(*f*)	$COOCH_3$	CH_3	Glc
219	3-*O*-*β*-D-glucopyranosyl (1 → 3)-*β*-D-glucuronopyranoside-28-*O*-*β*-D-glucopyranosyl oleanolic acid methyl ester	H	Glc	H	$COOCH_3$	CH_3	Glc
220	3-*O*-*β*-D-xylopyranosyl (1 → 2)-*β*-D-glucopyranosyl-28-*O*-*β*-D-glucopyranosyl oleanolic acid	Xyl	H	H	CH_2OH	CH_3	Glc

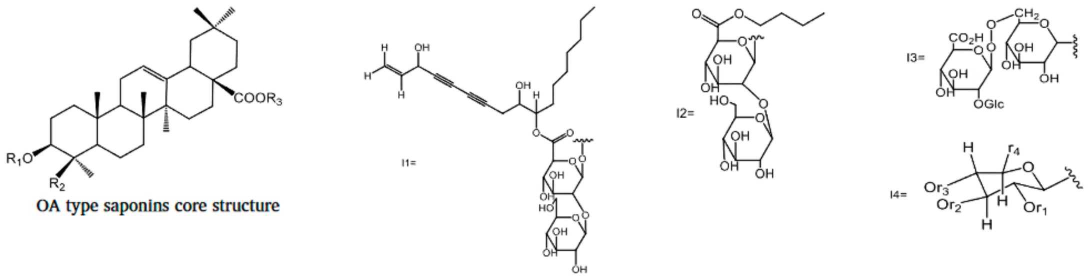

Figure S6. The structure of compounds in *Panax ginseng* C.A mey (Part.3)

Table S6 Chemical compositions in *Panax ginseng* C.A mey (Part.4)

NO.	Compound	R_1	R_2	R_3	C_{20}	C_{24}
221	Gypenoside F_{11}	H	*O*-Glc-Rha	CH_3	*R*	*S*
222	(20*R*,24*R*)-dammarane-20,24-epoxy-3*β*,6*α*,12*β*,25-tetraol	H	H	CH_3	*R*	*R*
223	Vina-ginsenosides R_{14}	H	*O*-Glc^2-Xyl	CH_3	*S*	*R*
224	24(*R*)-Ocotillol	H	OH	CH_3	*S*	*R*
225	Pseudoginsenoside F_{11}	H	*O*-Glc^2-Rha	CH_3	*S*	*R*
226	24(*R*)-majoroside R_1	H	OH	CH_3	*S*	*R*
227	(20*S*,24*R*,25*R*)-6-*O*-[*β*-D-glucopyranosyl-(1 → 2)-*β*-D-glucopyranosyl]-dammar-20,24-epoxy-3*β*,6*α*,12*β*,25,26-pentaol	H	*O*-Glc^2-Glc	CH_2OH	*S*	*R*
228	(20*S*,24*R*)-dammarane-20,24-epoxy-3*β*,6*α*,12*β*,25-tetraol	H	OH	CH_3	*S*	*R*
229	Pseudoginsenoside RT_5	H	*O*-Glc	CH_3	*S*	*R*
230	Pseudoginsenoside RT_2	H	*O*-Glc^2-Xyl	CH_3	*S*	*R*
231	(20*S*,24*R*)-Pseudoginsenoside F_{11}	H	*O*-Glc^2-Rha	CH_3	*S*	*R*
232	Vina-ginsenosides R_1	H	AC-Glc^2-Rha	CH_3	*S*	*S*
233	Vina-ginsenosides R_2	H	AC-Glc^2-Xyl	CH_3	*S*	*S*
234	Vina-ginsenosides R_5	H	Glc^2-Xyl^4-Glc	CH_3	*S*	*S*
235	Vina-ginsenosides R_6	H	Glc^6-Glc^2-Xyl	CH_3	*S*	*S*
236	Vina-ginsenosides R_{13}	H	Glc^2-Xyl	CH_3	*S*	*S*
237	(20*S*,24*S*,25*R**)-6-*O*-[*β*-D-glucopyranosyl-(1 → 2)-*β*-D-glucopyranosyl]-dammar-20,24-epoxy-3*β*,6*α*,12*β*,25,26-pentaol	H	Glc^2-Glc	CH_2OH	*S*	*S*
238	Pseudo-ginsenoside RT_4	H	Glc	CH_3	*S*	*S*
239	24(*S*)-majoroside R_1	H	Glc^2-Glc	CH_3	*S*	*S*
240	24(*S*)-6-*O*-[*β*-D-glucopyranosyl-(1 → 2)-*β*-D-glucopyranosyl]-dammar-20,25-epoxy-3*β*,6*α*,12*β*,24*α*-tetraol	H	Glc^2-Glc	CH_3	*S*	*S*
241	24(*S*)-Majoroside R_2	H	Glc^2-Xyl	CH_3	*S*	*S*
242	(20*S*,24*S*)-dammarane-20,24-epoxy-3*β*,6*α*,12*β*,25-tetraol	H	H	CH_3	*S*	*S*
243	Yesanchinoside A (24*S*)	H	AC-$^6Glc^2$-Glc	CH_3	*S*	*S*
244	Yesanchinoside B (24*S*)	H	Glc-$^6Glc^2$-Glc	CH_3	*S*	*S*
245	Yesanchinoside C (24*S*)	H	Glc^2-Glc^2-Xyl	CH_3	*S*	*S*

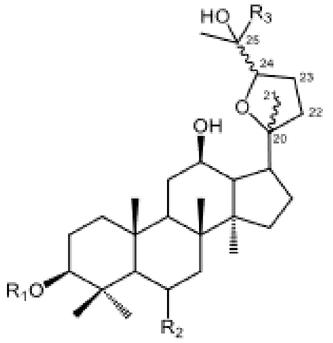

OC type saponins core structure

Figure S7 The structure of compounds in *Panax ginseng* C.A mey(Part.4)

Table S7 Chemical compositions in *Panax ginseng* C.A mey(Part.5)

NO.	Type	Compound	R_1	R_2	R_3	R_4	Chiral Carbon
246	V1	25-hydroxy-23-ene-20(*S*)-protopanaxadiol	H	OH	OH	–	C24:*S*
247	V1	Ginsenoside-M6a	Glc^2-Glc	OH	*O*-Glc	–	C25:*S*
248	V1	Floranotoginsenoside A	Glc^2-Glc	OH	*O*-Glc^6-Ara(*f*)	–	C25:*S*
249	V1	Gypenoside XLIX	Glc^2-Glc	OH	*O*-Glc^6-Xyl	–	C25:*S*
250	V1	Notoginsenoside L_{16}	Glc	OH	*O*-Glc^6-Ara(*f*)	–	C25:*S*
251	V1	Vina-ginsenosides R_8	Glc^2-Glc	OH	*O*-Glc	–	C25:*R*
252	V1	Dammar-23 (24)-ene-3*β*,12*β*, 20(*S*), 25-tetraol-20-*O*-*β*-D-glucopyranosyl-3-*O*-*β*-D-glucopyranoside	Glc	OH	Glc	–	C25:*R*
253	V1	Majoroside F_4	Glc	OH	*O*-Glc	–	C25:*R*
254	V1	dammar-23 (24)-ene-3*β*, 12*β*, 20(*S*), 25-tetraol–20-*O*-*β*-D-glucopyranoside	H	OH	*O*-Glc	–	C25:*R*
255	V2	Notoginsenoside Fh_6	Glc^2-Gen	OH	*O*-Glc	–	–
256	V3	Notoginsenoside SFt_2	Glc	OH	OH	–	–
257	V3	dammar-3*β*,12*β*,20(*S*),24(*ζ*),25-pentaol-20-*O*-*β*-D-glucopyranoside	H	OH	*O*-Glc	–	–
258	V3	Chikusetsusaponin FM_1	Glc^2-Glc	OH	*O*-Glc^2-Xyl	–	–
259	V4	Notoginsenoside R_7	Glc	OH	–	–	–

Table S7(continued)

NO.	Type	Compound	R$_1$	R$_2$	R$_3$	R$_4$	Chiral Carbon
260	V4	3*β*-acetoxy-12*β*-hydroxy-20 (*R*), 25-epoxy dammarane	AC	OH	–	–	–
261	V5	Notoginsenoside SFt$_1$	Glc	OH	OH	–	C24:*R*
262	V5	Notoginsenoside Fh$_3$	Glc^2-Glc^2-Xyl	OH	*O*-Glc^2-Xyl	–	C24:*R*
263	V5	Notoginsenoside Fh$_4$	Glc^2-Glc^2-Xyl	OH	*O*-Glc^6-Glc	–	C24:*R*
264	V5	Floranotoginsenoside D	Glc^2-Glc	OH	*O*-Glc^6-Ara(*f*)	–	C24:*R*
265	V5	Notoginsenosides LK$_6$	Glc^2-Glc	OH	*O*-Xyl	–	C24:*R*
266	V5	Notoginsenosides LK$_7$	Glc^2-Glc	OH	*O*-Glc^6-Ara(*f*)	–	C24:*R*
267	V5	Notoginsenosides LK$_8$	Glc^2-Glc^2-Xyl	OH	OH	–	C24:*R*
268	V5	Vinaginsenosides R$_9$	Glc^2-Glc	OH	*O*-Glc	–	C24:*S*
269	V5	Bipinnatifidusoside F$_1$	Glc^2-Glc	OH	*O*-Glc	–	C24:*R*
270	V5	Majoroside F$_1$	Glc^2-Glc	OH	*O*-Glc	–	C24:*R*
271	V6	Notoginsenoside Ft$_2$	Glc^2-Glc^2-Xyl	OH	OH	–	–
272	V7	Ginsenoside II	Glc^2-Glc	OH	*O*-Glc	OH	–
273	V7	Floranotoginsenoside B	Glc^2-Glc	OH	*O*-Glc^2-Xyl	OH	–
274	V7	Floranotoginsenoside C	Glc^2-Glc	OH	*O*-Glc^6-Ara(*f*)	OH	–
275	V7	Floralquinquenoside D	Glc	OH	*O*-Glc	OH	–
276	V8	Ginsenoside III	Glc^2-Glc	OH	*O*-Glc	–	–
277	V8	Notoginsenoside L$_{17}$	Glc	OH	*O*-Glc^6-Xyl	–	–
278	V8	Notoginsenoside L$_{18}$	Glc	OH	*O*-Glc^6-Ara(*f*)	–	–
279	V8	Notoginsenoside L$_{19}$	Glc	OH	*O*-Glc^6-Ara(*f*)	–	–
280	V9	Ginsenoside Rg$_{12}$	Glc-Glc^2	OH	OH	OH	–
281	V10	Ginsenoside L$_1$	Glc	OH	OH	–	–
282	V11	Ginsenoside L$_2$	Glc^2-Glc	OH	OH	–	–
283	V12	Notoginsenosides Ng$_2$	Glc^2-Glc	OH	*O*-Glc^6-Ara(*f*)	–	–
284	V12	Notoginsenosides LK$_1$	Glc^2-Glc	OH	*O*-Glc^6-Xyl	–	–
285	V12	Notoginsenosides LK$_4$	Glc^2-Glc^2-Xyl	OH	*O*-Glc^6-Xyl	–	–
286	V12	Notoginsenosides LK$_5$	Glc^2-Glc^2-Xyl	OH	*O*-Glc^6-Ara(*p*)	–	–
287	V12	Vinaginsenoside R$_{20}$	Glc^2-Glc	OH	*O*-Glc	–	–
288	V13	Notoginsenosides LK$_2$	Glc^2-Glc^2-Xyl	OH	*O*-Glc-Ara(*f*)	–	–
289	V13	Notoginsenosides LK$_3$	Glc^2-Glc^2-Xyl	OH	*O*-Glc	–	–
290	V13	Notoginsenosides LK$_{15}$	Glc	OH	*O*-Glc^6-Xyl	–	–
291	V14	Notoginsenoside L$_1$	H	OH	–	–	–
292	V15	Notoginsenoside L$_2$	Glc^2-Glc	OH	OH	–	–
293	V16	Notoginsenoside L$_3$	Glc	OH	–	–	–
294	V17	Sanchirhinoside D	Glc^2-Glc	OH	OH	–	–
295	V18	Koryoginsenoside R$_2$	Glc^2-Glc	OH	*O*-Glc^6-Glc	–	–
296	V19	3*β*,6*β*,12*β*,20(S)-trihydroxy dammar 24-methyl 1-23-ene-24-carbonyl	H	OH	OH	–	–
297	V20	3*β*,12*β*,20(*S*),25-tetrahydroxy dammar 23-ene	H	OH	OH	–	–
298	V20	Quinquenoside L$_3$	Glc	OH	*O*-Glc^6-Xyl	–	–
299	V21	27-demethyl-(*E*,*E*)-20 (22),23-dien-3*β*,12*β*-dihydroxydammar-25-one	H	OH	–	–	–
300	V22	20(*S*)-25-ethoxyl-dammarane-3*β*,12*β*,20-triol	H	OH	OH	CH$_3$	–
301	V22	20(*R*)-25-ethoxyl-dammarane-3*β*,12*β*,20-triol	H	OH	CH$_3$	OH	–
302	V23	Quinquenoside L$_1$	Glc^2-Glc	OH	*O*-Glc	–	–
303	V24	Isoginsenoside Rh$_3$	Glc	OH	–	–	–
304	V25	Floralginsenoside E	Glc^2-Glc	OH	OH	OH	–
305	V25	Floralginsenoside F	Glc	OH	*O*-Glc	OH	–
306	V26	Floralginsenoside Kb	Glc^2-Glc	OH	*O*-Glc	H	–
307	V26	Floralginsenoside K$_C$	Glc^2-Glc	OH	*O*-Glc	OH	–
308	V27	Bipinnatifidusoside F$_2$	Glc^2-Glc	OH	*O*-Glc	–	–
309	V28	Ginsenoside Rz$_1$	Glc^2-Glc	OH	–	–	–
310	V29	Vinaginsenoside R$_{24}$	Glc^2-Glc	OH	Glc	CH$_3$	–
311	V30	Ginsenoside RT$_5$	Glc	OH	CH$_3$	–	–

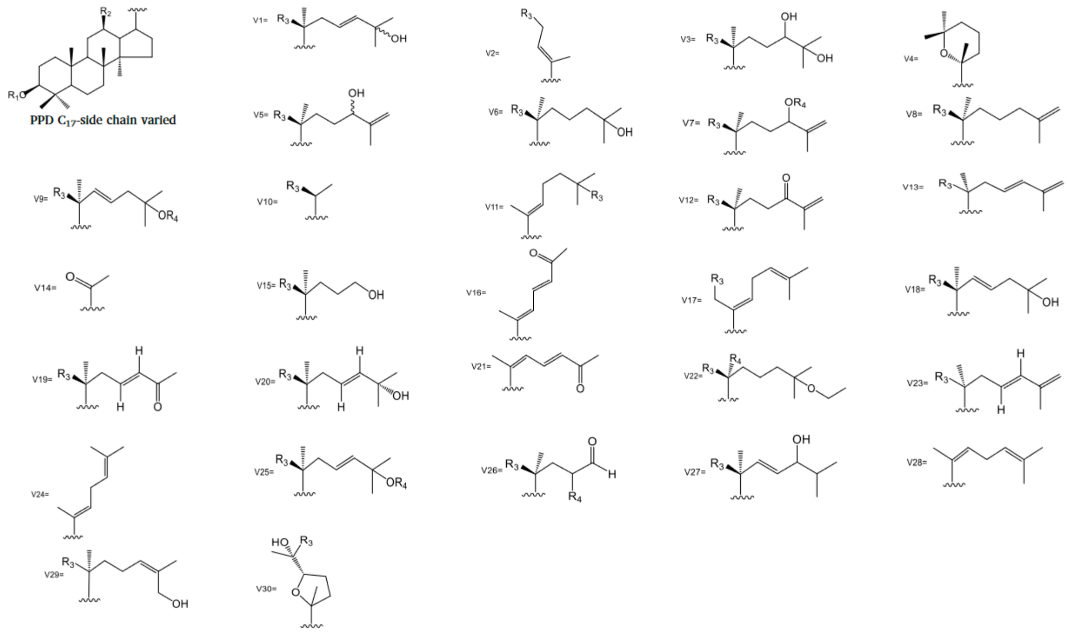

Figure S8. The structure of compounds in *Panax ginseng* C.A mey(Part.5)

Table S8 Chemical compositions in *Panax ginseng* C.A mey(Part.6)

NO.	Type	R_1	R_2	R_3	R_4	R_5	R_6	Chiral Carbon
312	W1	OH	OH	H	–	–	–	–
313	W1	OH	H	H	–	–	–	–
314	W1	OH	OH	H	–	–	–	–
315	W1	*O*-Glc	H	H	–	–	–	–
316	W1	OH	*O*-Glc	H	–	–	–	–
317	W2	OH	*O*-Glc	H	H	–	–	–
318	W2	OH	*O*-Glc^{2}-Xyl	H	H	–	–	–
319	W3	OH	*O*-Glc	H	H	–	–	–
320	W3	OH	*O*-Glc^{2}-Rha	H	Glc	–	–	–
321	W3	OH	*O*-Glc^{2}-Rha	H	H	–	–	–
322	W4	OH	*O*-Glc	H	Glc	–	–	–
323	W4	OH	*O*-Glc	H	H	–	–	–
324	W4	OH	*O*-Glc	H	H	–	–	–
325	W4	OH	*O*-Glc^{2}-Glc	H	H	–	–	–
326	W4	*O*-Glc^{2}-Glc	H	H	Glc	–	–	–
327	W4	OH	*O*-Glc	H	Glc	–	–	–
328	W5	OH	OH	H	OH	CH_3	–	C25:*S*
329	W5	OH	*O*-Glc^{2}-Rha	H	OH	CH_3	–	C25:*S*
330	W5	OH	OH	H	CH_3	OH	–	C25:*S*
331	W5	OH	*O*-Glc^{2}-Rha	H	CH_3	OH	–	C25:*S*
332	W5	OH	H	H	OH	CH_3	–	C25:*R*
333	W5	OH	H	H	CH_3	OH	–	C25:*R*
334	W5	OH	OH	H	OH	CH_3	–	C25:*R*
335	W5	OH	OH	H	CH_3	OH	–	C25:*R*
336	W5	OH	*O*-Glc^{2}-Rha	H	CH_3	OH	–	C25:*R*
337	W5	OH	*O*-Glc^{2}-Rha	H	OH	CH_3	–	C25:*R*
338	W6	OH	*O*-Glc^{2}-Xyl	H	OH	*O*-Glc	–	–
339	W6	*O*-Glc^{2}-Glc	OH	H	OH	*O*-Glc	–	–
340	W6	OH	OH	H	OH	*O*-Glc^{2}-Xyl	–	–
341	W6	*O*-Glc^{2}-Glc^{2}-Xyl	H	H	OH	*O*-Glc^{6}-Glc	–	–
342	W6	OH	H	H	OH	OH	–	–
343	W6	OH	Glc	H	OH	*O*-Glc	–	–
344	W6	OH	*O*-Glc^{2}-Rha	H	OH	*O*-Glc	–	–
345	W6	OH	*O*-Glc	H	OH	OH	–	–
346	W6	OH	OH	H	CH_3	*O*-Glc	–	–
347	W6	OH	OH	H	OH	*O*-Glc	–	–
348	W6	OH	OH	H	OH	*O*-Glc	–	–
349	W6	*O*-Glc^{2}-Glc	OH	H	OCH_3	*O*-Glc^{6}-Xyl	–	–
350	W6	OH	*O*-Glc	H	OOH	OH	–	–
351	W6	*O*-Glc^{2}-Glc^{2}-Xyl	OH	H	OOH	*O*-Glc^{6}-Glc	–	–
352	W6	= O	OH	H	OOH	OH	–	–
353	W6	H	OH	= O	OOH	OH	–	–
354	W6	= O	H	H	OOH	OH	–	–
355	W6	OH	*O*-Glc	H	OOH	*O*-Glc	–	–
356	W6	OH	OH	H	OOH	*O*-Glc^{6}-Ara(f	–	–
357	W6	OH	*O*-Glc	H	OOH	OH	–	–
358	W6	OH	*O*-Glc^{2}-Rha	H	OOH	OH	–	–
359	W6	OH	OH	H	OOH	*O*-Glc	–	–
360	W6	OH	*O*-Glc	H	OOH	OH	–	–
361	W7	*O*-Glc^{2}-Glc^{2}-Xyl	H	H	Glc^{6}-Glc	–	–	–
362	W7	OH	*O*-Glc^{2}-Rha	H	Glc	–	–	–
363	W7	OH	Glc	H	Glc	–	–	–
364	W7	OH	OH	H	Glc	–	–	–
365	W8	*O*-Glc^{2}-Glc	OH	H	Glc^{6}-Glc	OH	–	C24:*R*
366	W8	OH	*O*-Glc	H	H	OH	–	C24:*R*
367	W12	OH	*O*-Glc^{2}-Glc	H	H	–	–	C24:*S*
368	W8	OH	OH	H	Glc^{6}-Ara(*p*)	OH	–	C24:*R*
369	W8	OH	*O*-Glc	H	Glc	OH	–	C24:*R*
370	W8	*O*-AC-^{6}Glc^{2}-Glc	H	H	Glc	OH	–	C24:*R*
371	W8	*O*-Glc^{2}-Glc	H	H	Glc^{6}-Ara(*p*)	OH	–	C24:*R*
372	W8	*O*-Glc^{2}-Glc	H	H	Glc^{6}-Ara(*p*)	OH	–	C24:*R*
373	W8	OH	*O*-Glc^{2}-Rha	H	Glc	OH	–	C24:*R*
374	W8	OH	OH	H	Glc	OH	–	C24:*R*
375	W8	OH	*O*-Glc^{2}-Rha	H	H	OH	–	C24:*R*
376	W8	OH	OH	H	Glc	OH	–	C24:*S*
377	W9	OH	*O*-Glc	H	–	–	–	–
378	W10	OH	*O*-Glc	H	H	–	–	–
379	W10	OH	OH	H	H	–	–	–
380	W11	OH	*O*-Glc	H	H	–	–	–
381	W11	OH	*O*-Glc	H	CH_3	–	–	–
382	W11	*O*-Glc^{2}-Rha	OH	H	H	–	–	–
383	W12	OH	*O*-Glc^{3}-Xyl	H	–	–	–	–
384	W12	OH	*O*-Glc^{2}-Rha	H	–	–		
385	W12	OH	OH	H	–	–	–	–
386	W12	*O*-Glc^{2}-Glc^{2}-Xyl	H	H	–	–	–	–
387	W13	OH	*O*-Glc	H	H	–	–	–
388	W13	*O*-Glc^{2}-Glc	H	H	OCH_3	–	–	–
389	W14	*O*-Glc^{2}-Glc	H	H	OCH_3	–	–	–
390	W15	OH	*O*-Glc	H	–	–	–	–
391	W15	OH	OH	H	–	–	–	–
392	W16	OH	Glc^{2}-Rha	H	–	–	–	–
393	W16	*O*-Glc^{2}-Glc^{2}-Xyl	H	H	–	–	–	–

Table S8(continued)

NO.	Type	R_1	R_2	R_3	R_4	R_5	R_6	Chiral Carbon
394	W16	OH	OH	H	–	–	–	–
395	W16	*O*-Glc^{2}-Glc^{6}-AC	H	H	–	–	–	–
396	W16	OH	*O*-Glc^{2}-Rha	H	–	–	–	–
397	W17	OH	*O*-Glc	H	–	–	–	–
398	W18	OH	*O*-Glc	H	–	–	–	–
399	W19	OH	*O*-Glc	H	–	–	–	–
400	W19	*O*-Glc	OH	H	–	–	–	–
401	W20	OH	*O*-Glc^{2}-Glc	H	–	–	–	–
402	W21	*O*-Glc^{2}-Xyl	OH	H	–	–	–	–
403	W21	*O*-Glc	OH	H	–	–	–	–
404	W21	OH	OH	H	–	–	–	–
405	W21	OH	*O*-Glc^{2}-Rha	H	–	–	–	–
406	W21	OH	*O*-Glc	Glc	–	–	–	–
407	W22	OH	*O*-Glc	H	–	–	–	–
408	W23	OH	*O*-Glc	H	OH	–	–	C24:*R*
409	W23	OH	*O*-Glc^{2}-Glc	H	OH	–	–	C24:*R*
410	W23	OH	*O*-Glc^{2}-Rha	H	*O*-Glc	–	–	C24:*R*
411	W23	OH	*O*-Glc^{2}-Rha	H	*O*-Glc	–	–	C24:*R*
412	W23	OH	OH	H	*O*-Glc	–	–	C24:*S*
413	W23	OH	OH	H	*O*-Glc	–	–	C24:*S*
414	W23	OH	Glc	H	OH	–	–	C24:*R*
415	W24	OH	OH	H	*O*-Glc	CH_3	–	–
416	W25	OH	*O*-Glc	H	*O*-Glc^{6}-Ara(*f*)	CH_3	–	–
417	W26	*O*-Glc^{2}-Glc	OH	H	–	–	–	–
418	W27	OH	OH	H	O–CH_2CH_3	–	–	–
419	W27	OH	OH	H	OH	–	–	–
420	W27	*O*-Glc	H	H	OH	–	–	–
421	W27	OH	H	H	OH	–	–	–
422	W27	*O*-AC	OH	H	OH	–	–	–
423	W27	OH	OH	AC	OH	–	–	–
424	W28	OH	OH	H	*O*-Glc	–	–	–
425	W29	OH	OH	H	–	–	–	–
426	W30	OH	*O*-Glc	H	CH_3	–	–	–
427	W30	*O*-Glc	H	H	CH_3	–	–	–
428	W30	*O*-Glc^{2}-Glc	OH	H	CH_3	–	–	–
429	W30	OH	*O*-Glc	H	OH	–	–	–
430	W30	OH	*O*-Glc	H	OOH	–	–	–
431	W30	OH	*O*-Glc^{2}-Rha	H	OOH	–	–	–
432	W31	OH	*O*-Glc	H	CH_3	CH_3	–	C24:*S*
433	W31	OH	*O*-Glc^{2}-Rha	H	–	*O*-Glc	–	C24:*R*
434	W32	OH	*O*-Glc	H	OH	–	–	C22:*S*
435	W33	OH	*O*-Glc	H	OH	–	–	–
436	W34	OH	*O*-Glc	H	–	–	–	–
437	W34	OH	*O*-Glc^{2}-Xyl	H	–	–	–	–
438	W35	OH	OH	H	CH_3	OH	–	–
439	W35	OH	*O*-Glc	H	OH	CH_3	–	–
440	W35	OH	*O*-Glc^{2}-Xyl	H	OH	CH_3	–	–
441	W35	OH	*O*-Glc^{2}-Glc	H	OH	CH_3	–	–
442	W35	OH	OH	H	*O*-Glc	CH_3	–	–
443	W36	OH	OH	H	–	–	–	C24:*S*
444	W36	OH	OH	H	–	–	–	C24:*R*
445	W37	OH	$OCOCH_3$	H	–	–	–	–
446	W38	OH	OH	H	OH	CH_3	–	–
447	W38	OH	*O*-Glc	H	CH_3	OH	–	–
448	W38	*O*-Glc	H	H	OH	CH_3	–	–
449	W39	OH	H	H	CH_3	OH	CH_3	–
450	W39	OH	H	H	H	OCH_3	H	–
451	W39	OH	OH	H	H	OH	CH_3	–
452	W39	OH	*O*-Glc^{2}-Rha	H	H	OH	CH_3	–
453	W40	OH	OH	H	–	–	–	–
454	W40	OH	*O*-Glc	H	–	–	–	–
455	W41	OH	OH	H	–	–	–	–
456	W42	OH	OAC	H	–	–	–	–
457	W42	AC	OH	H	–	–	–	–
458	W43	*O*-AC-^{6}Glc^{2}-Glc	H	H	Glc	OH	–	–
459	W43	OH	*O*-Glc^{2}-Rha	H	Glc	OH	–	–
460	W43	*O*-Glc^{2}-Glc	H	H	Glc	OH	–	–
461	W43	*O*-Glc^{2}-Glc	H	H	Glc^{6}-Ara(*f*)	OH	–	–
462	W44	OH	OH	H	*O*-Glc	–	–	–
463	W45	OH	*O*-Glc	H	–	–	–	–
464	W45	OH	*O*-Glc^{2}-Rha	H	–	–	–	–

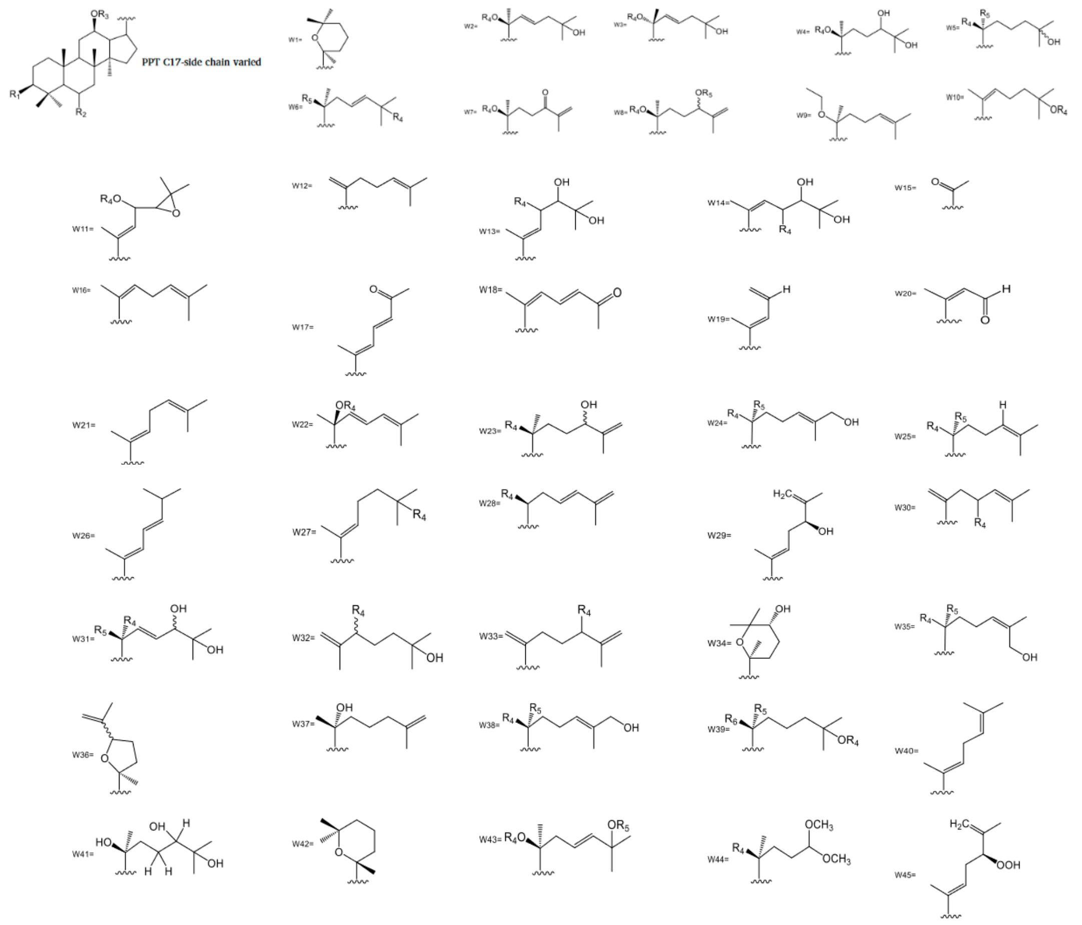

Figure S9. The structure of compounds in *Panax ginseng* C.A mey(Part.6)

Table S9 Chemical compositions in *Panax ginseng* C.A mey (Part.7)

NO.	Type	Compound	R_1	R_2	R_3	R_4
465	A	Baisanqisaponin A	H	H	X	Glc
466	A	Baisanqisaponin B	H	Ara(*f*)	X	Glc
467	A	Baisanqisaponin C	Glc	H	X	H
468	B	Pseudo-ginsenoside RT_6	*O*-Glc	–	–	–
469	B	Pseudoginsengenin R_1	OH	–	–	–
470	C	Gypenoside L	Glc^2-Glc	H	–	–
471	C	Gypenoside L_1	Glc^2-Glc	H	–	–
472	D	24(*R*)-Pseudoginsenoside G_1	Glc^2-Glc	–	–	–
473	D	24(*S*)-Pseudoginsenoside G_2	Glc^2-Glc	–	–	–
474	E	3*β*,4*α*,12*β*-trihydroxystigmast-5-en-21-yl octadecan-9′,12′-dienoate	R = $CO(CH_2)_7CH{=}CHCH_2CH{=}CH(CH_2)_4CH_3$			
475	E	stigmast-5-en-3*β*,4*α*,12*β*, 21-tetraol-21-octadec-9′,12′-dienoate	R = $CO(CH_2)_7CH{=}CHCH_2(CH_2)_3CH_3$			
476	F	3*β*-*cis*-feruloyloxy-16*β*-hydroxylup-20 (29)-ene	S1"	–	–	–
477	F	3*β*-*trans*-feruloyloxy-16*β*-hydroxylup-20 (29)-ene	S2"	–	–	–
478	G	Notoginsenoside G	Glc^2-Glc	Glc	–	–
479	G	Yesanchinoside G	Glc^2-Glc	Glc^6-Xyl	–	–
480	G	Quinquenoside IV	Glc^2-Glc	Glc^6-Glc	–	–
481	H	Notoginsenoside I	Glc^2-Glc	*O*-Glc^6-Glc	–	–
482	H	Yesanchinoside I	Glc^2-Glc	*O*-Glc^6-Glc-Xyl	–	–
483	H	Lanost-24-en-3*β*-ol-3-*O*-*β*-D-arabinopyranosyl-(2′→1″)-*O*-*β*-D-arabinoside	Glc^2-Ara(*p*)	CH_3	–	–
484	I	Vina-ginsenoside R_3	Glc^2-Glc	H	Glc	–
485	J	Chikusetsusaponins LT_5	Glc	H	Glc^6-Glc	–
486	J	Chikusetsusaponins LT_8	Glc	H	Glc	–
487	J	Chikusetsusaponin LN_4	Glc^6-Xyl	H	Glc^6-Ara (*p*)	–
488	J	3*β*,6*α*-20(*S*)-6,20-bis(*β*-D-glucopyranosyloxy)-3-hydroxy dammar-24-en-12-one	H	Glc	Glc	–
489	J	Chikusetsusaponin FK_2	Glc^2-Glc	Glc	–	–
490	J	Chikusetsusaponin FK_3	Xyl-^{6}Glc^2-Glc	Glc	–	–
491	K	7*β*-hydroxyl ginsenoside Rd	*O*-Glc-Glc^2	Glc	–	–
492	L	5,6-didehydroginsenoside Rg_3	*O*-Glc-Glc^2	OH	–	–
493	L	5,6-didehydroginsenoside Rd	Glc^2-Glc	Glc	–	–
494	L	5,6-didehydroginsenoside Rb_1	Glc^2-Glc	Glc^6-Glc	–	–
495	M	Notoginsenoside LX	Glc	Glc^6-Ara(*f*)	–	–
496	M	Notoginsenoside LY	H	Glc^6-Ara(*f*)	–	–
497	M	Notoginsenosides Ng_1	Glc	Glc^6-Xyl	–	–
498	M	Notoginsenoside Fh_8	Glc-Glc	Glc-Ara(*f*)	–	–
499	M	Notoginsenoside Fh_9	Glc-Glc	Glc-Ara(*p*)	–	–
500	M	Notoginsenoside L14	Glc	Glc^6-Xyl	C23:*S*	–
501	M	Notoginsenoside L13	Glc	Glc^6-Xyl	C23:*R*	–

Table S9(continued)

NO.	Type	Compound	R_1	R_2	R_3	R_4
502	M	3*β*,20*S*-dihydroxy-12*β*, 23*R*-epoxydammar-24-ene 3-*O*-[*β*-D-glucopyranosyl (1 → 2)-*β*-D-glucopyranosyl]-20-*O*-*β*-D-glucopyranoside	Glc^{2}-Glc	*O*-Glc	–	–
503	M	Ginsenoside La	Glc	Glc	–	–
504	N	Ginsenosides Rh_{18}	OH	*O*-Glc^{2}-Rha	*O*-Glc	–
505	N	12*β*,23(*R*)-epoxydammara-24-ene-3*β*,6*α*,20(*S*)-triol	OH	OH	OH	–
506	N	3*β*,6*α*, 20S-trihydroxy-12*β*, 23*R*-epoxydammar-24-ene 6-*O*-[*α*-L-rhamnosyl (1 → 2)-*β*-D-glucopyranosyl]-20-*O*-*β*-D-glucopyranoside	H	*O*-Glc^{2}-Rha	*O*-Glc	–
507	P	Notoginsenoside P_1	Glc	OH	–	–
508	Q	6-*O*-*β*-D-glucopyranosyl-20*R*-protopanaxadiol-3-one	Glc	H	–	–
509	R	6-*O*-*β*-D-glucopyranosyl-20-*O*-*β*-D-glucopyranosyl-20(*S*)-protopanaxadiol-3-one	Glc	Glc	–	–
510	T	6*α*-hydroxy-22,23,24,25,26,27-hexanordammar-3,12,20-trione	Ara(*p*)	Ara(*p*)	–	–
511	S	Dammar-12,24-dien-3*α*,6*β*,15*α*-triol-3*α*-D-arabinopyranosyl-6*β*-L-arabinopyranoside	–	–	–	–
512	U	Dammar-24-en-3*α*,6*β*,16*α*,20*β*-tetraol-3*α*-D-arabinopyranosyl-6*β*-D-arabinopyranoside	Xyl	Xyl	–	–
513	V	(20*S*,22*S*)-dammar-22,25-epoxy-3*β*,12*β*,20-triol	–	–	–	–
514	W	Pseudoginsenoside-RI_2	–	–	–	–
515	X	12-one-pseudoginsenoside F_{11}	Glc^{2}-Rha	–	–	–
516	Y	(12*R*,20*S*,24*S*)-20,24-;12,24-diepoxy-dammarane-3*β*-ol	–	–	–	–

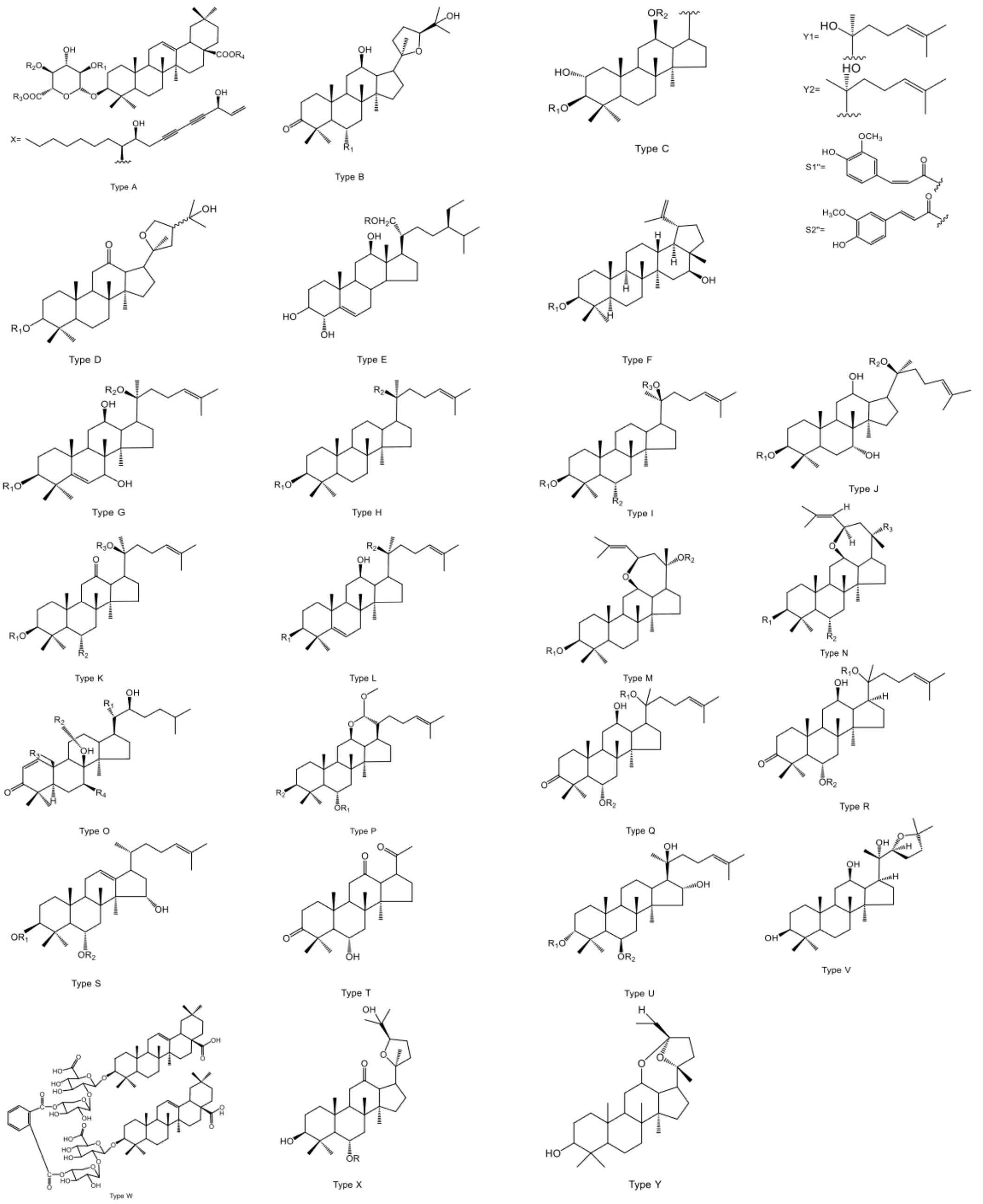

Figure S10. The structure of compounds in *Panax ginseng* C.A mey (Part.7)

Table S10 Chemical compositions in *Ziziphus jujuba* mill. Var

NO.	Compound	NO.	Compound
S1	Ursolic acid	F5	Epiafzelechin
S2	3-Oxours-12-en-28-oic acid	F6	Afzelechin
S3	Pomonic acid	F7	Rutoside
S4	Pomolic acid	F8	Isospinosin
S5	Corosolic Acid	F9	Spinosin
S6	2α,3β,13β,23-Tetrahydroxyurs-11-en-28-oic acid	F10	6'''-sinapoyl spinosin
S7	2α,3β-Dihydroxyurs-20-en-28-oic acid	F11	6'''-feruloyl spinosin
S8	2α,3β,28-Trihydroxyurs-20-en	F12	6'''-coumaroyl spinosin
S9	3β,12β,13β-Trihydroxyurs-28-oic acid	A-1	6-(2',3'-dihydroxy-4'-hydroxymethyl-tetrahydro-furan-1'-yl) cyclopentadiene[c]pyrrole-1,3-diol
S10	Betulinic acid		
S11	Alphitolic acid	A-2	3S-1-N-β-D-glucopyranosyl-2-oxo-3-hydroxy-indole-3-acetic acid
S12	Betulonic acid	A-3	3R-1-N-β-D-glucopyranosyl-2-oxo-3-hydroxy-indole-3-acetic acid
S13	3-O-trans-p-Coumaroylalthitolic acid	A-4	Coclaurine
S14	3-O-cis-p-Coumaroylalthitolic acid	A-5	Juzirine
S15	Betulin	A-6	Norisocorynoline
S16	2α, 3β-dihydroxy-lup-20(29)-en-28-oic acid methyl ester	A-7	Caaverine
S17	3β-Hydroxy-30-norlupan-20-one-28-oic acid	A-8	Asimilobine
S18	Oleanic acid	A-9	Nornuciferine
S19	Oleanonic acid	A-10	N-Methylasimilobine
S20	3-O-Trans-p-coumaroyl maslinic acid	A-11	Nuciferine
S21	3-O-cis-p-coumaroyl maslinic acid	A-12	4H-Dibenzo[de,g]quinolinium
S22	Hydroxyoleanonic acid lacton	A-13	(+)-magnoflorine iodide
S23	Maslinic acid	A-14	Lysicamine
S24	Epiceanothic acid	A-15	6-glc-coclaurine
S25	Ceanothic acid	A-16	Sanjoinine A
S26	Zizyberenalic acid	A-17	Sanjoinine F
S27	Colubrinic acid	A-18	Sanjoinine B
S28	Ceanothenic acid	A-19	Sanjoinine D
S29	Cecropiacic acid	A-20	Sanjoinine G1
S30	Zizyphus saponin I	A-21	Lotusine B
S31	Zizyphus saponin II	A-22	Ramosine A
F1	Pinocembrin	A-23	Sanjoinine G2
F2	7, 4'-dihydroxy-5-methoxy flavanon	A-24	Amphibine D
F3	5,7,3',4'-tetramethoxycatechin	A-25	Sanjoinenine
F4	Catechin	A-26	Mucronine J

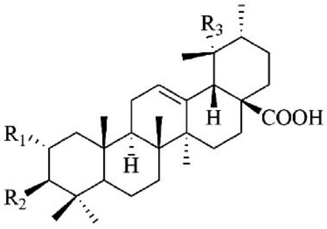

S1 R_1=H, R_2=OH, R_3=H
S2 R_1=H, R_2=O, R_3=H
S3 R_1=H, R_2=O, R_3=OH
S4 R_1=H, R_2=OH, R_3=OH
S5 R_1=OH, R_2=OH, R_3=H

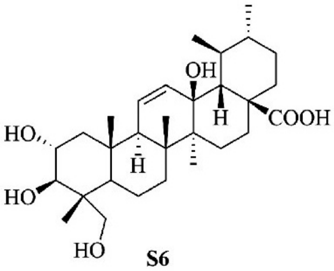

S6

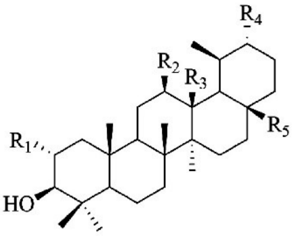

S7 R_1=OH, R_2=R_3=H, R_4=O, R_5=COOH
S8 R_1=OH, R_2=R_3=H, R_4=O, R_5=CH_2OH
S9 R_1=H, R_2=R_3=OH, R_4=H, R_5=COOH

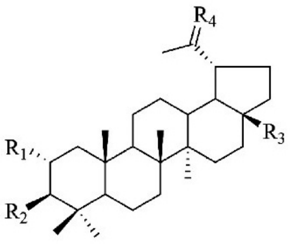

S10 R_1=H, R_2=OH, R_3=COOH, R_4=CH_2
S11 R_1=R_2=OH, R_3=COOH, R_4=CH_2
S12 R_1=H, R_2=O, R_3=COOH, R_4=CH_2
S13 R_1=OH, R_2=*cis-p*-coumaroyl, R_3=COOH, R_4=CH_2
S14 R_1=OH, R_2=*trans-p*-coumaroyl, R_3=COOH, R_4=CH_2
S15 R_1=H, R_2=OH, R_3=CH_2OH, R_4=CH_2
S16 R_1=OH, R_2=OH, R_3=$COOCH_3$, R_4=CH_2
S17 R_1=H, R_2=OH, R_3=COOH, R_4=O

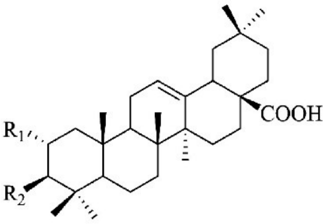

S18 R_1=H, R_2=OH
S19 R_1=H, R_2=O
S20 R_1=OH, R_2=*cis-p*-coumaroyl
S21 R_1=OH, R_2=*trans-p*-coumaroyl
S23 R_1=OH, R_2=OH

Figure S11. The structure of compounds in *Ziziphus jujuba* mill. Var

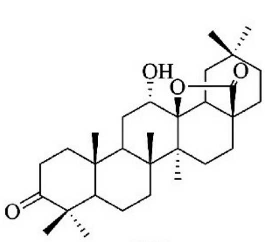

S22

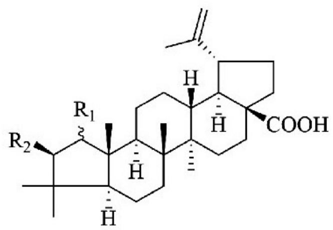

S24 R_1=αCOOH, R_2=αOH
S25 R_1=βCOOH, R_2=αOH
S27 R_1=αCHO, R_2=βOH

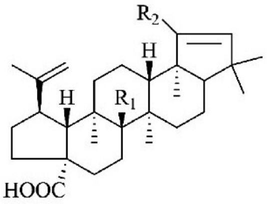

S26 R_1=CH_3, R_2=CHO
S28 R_1=COOH, R_2=H

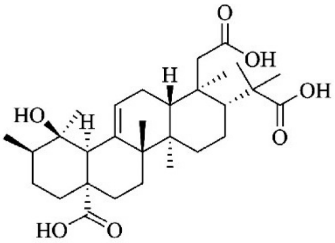

S29

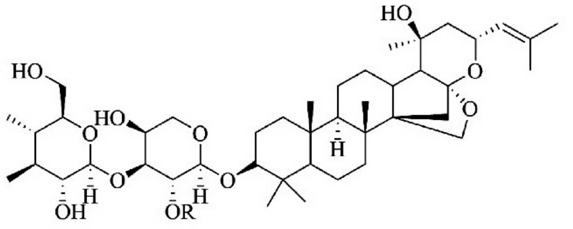

S30 R=*O*-6-deoxy-α-*L*-Tal
S31 R=*O*-6-deoxy-α-*L*-Man

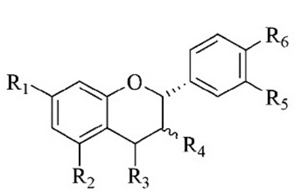

F1 R_1=R_2=OH, R_3=O, R_4=R_5=R_6=H
F2 R_1=R_6=OH, R_2=OCH_3, R_3=O, R_4=R_5=H
F3 R_1=R_2=R_5=R_6=OCH_3, R_3=H, R_4=βOH
F4 R_1=R_2=R_5=R_6=OH, R_3=H, R_4=βOH
F5 R_1=R_2=R_6=OH, R_3=R_5=H, R_4=αOH
F6 R_1=R_2=R_6=OH, R_3=R_5=H, R_4=βOH

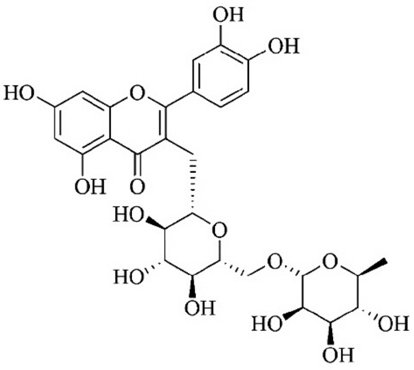

F7

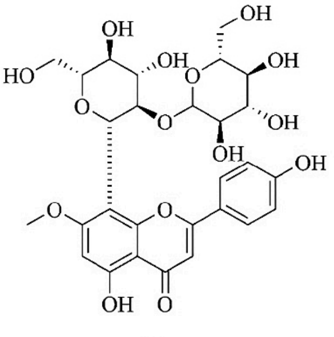

F8

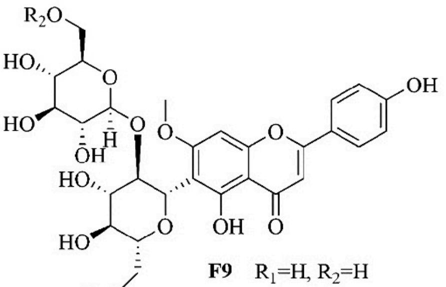

F9 R_1=H, R_2=H
F10 R_1=H, R_2=sinapoyl
F11 R_1=H, R_2=feruloyl
F12 R_1=H, R_2=*p*-coumaroyl

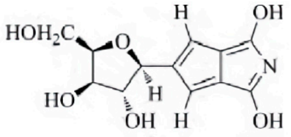

A-1

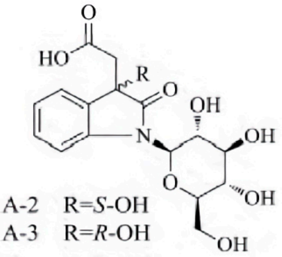

A-2 R=*S*-OH
A-3 R=*R*-OH

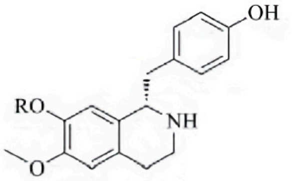

A-4 R=H
A-15 R=Glu

Figure S11 (continued)

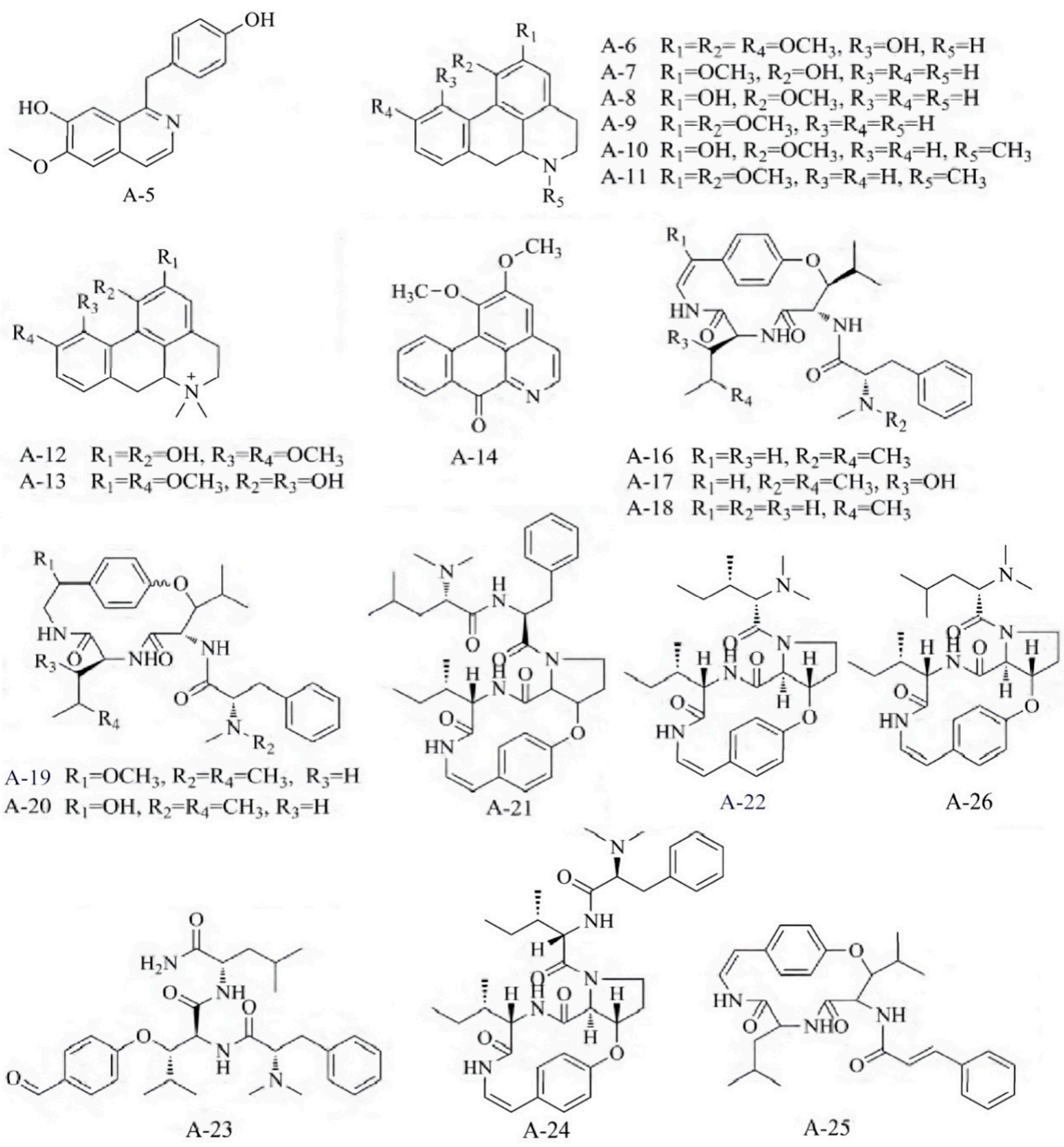

Figure S11 (continued)

Table S11 Chemical compositions in *Bambusa tuldoids* munro

No.	Compound	No.	Compound
**1**	(1S,2R)-1,2-bis(4-hydroxy-3-methoxyphenyl)-1,3-propanediol	**9**	(+)-lyoniresinol
**2**	2-Propenal, 3-[4-[(1S,2R)-2-hydroxy-2-(4-hydroxy-3-methoxyphenyl)-1-(hydroxymethyl)ethoxy]-3-methoxyphenyl]-, (2E)-	**10**	Isolariciresinol 9,9'-acetonide
		**11**	2,6-Dimethyl-4H-pyran-4-one
**3**	erythro-Guaiacylglycerol β-dihydroconiferyl ether	**12**	Friedelin
**4**	5'-Methoxylariciresinol	**13**	Friedelanol
**5**	5,5'-Dimethoxylariciresil	**14**	Lupenone
**6**	(+)-glaberide I	**15**	Lupeol
**7**	(-)-Syringaresinol	**16**	alpha-Amyrin
**8**	(-)-medioresinol		

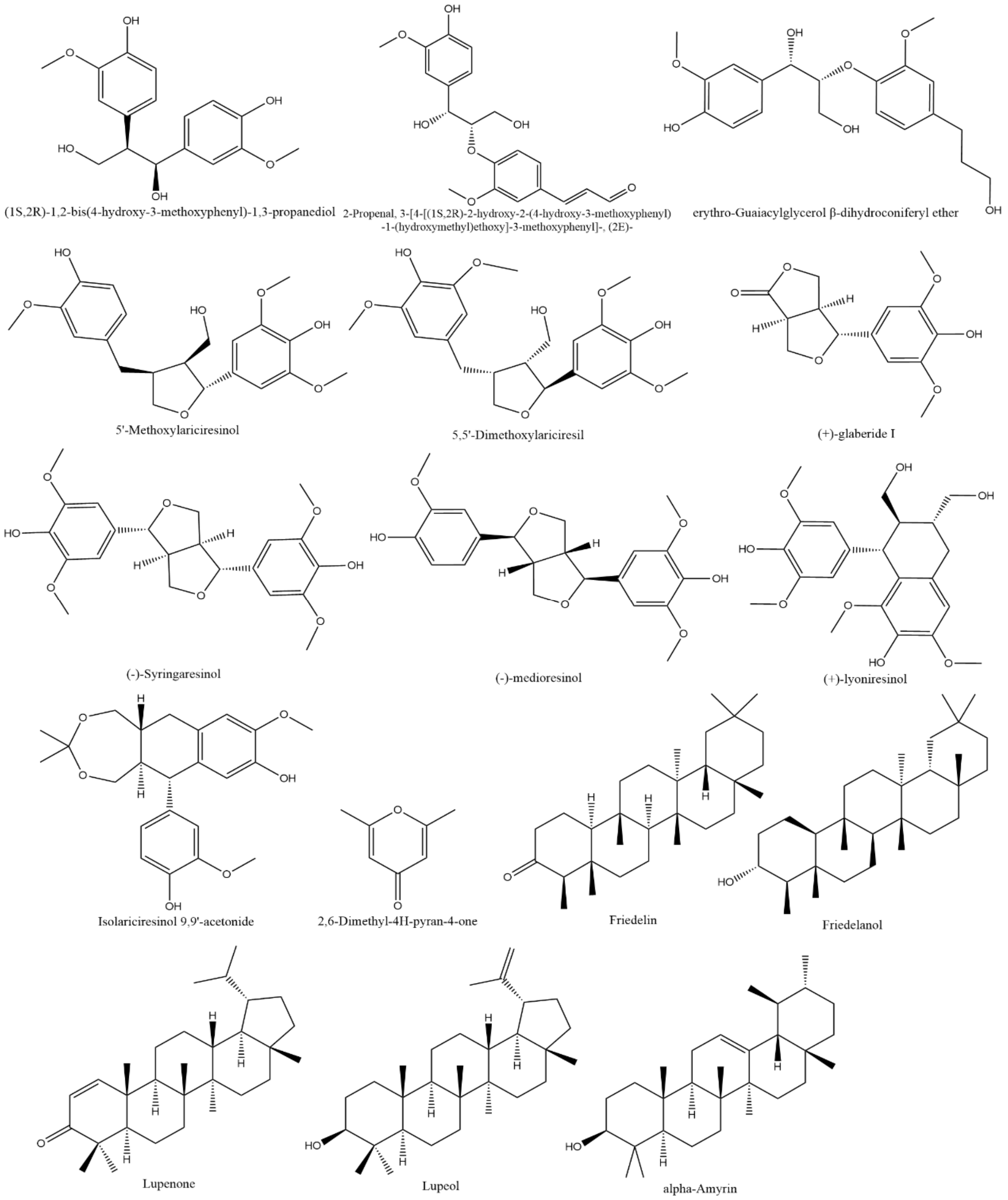

Figure S12. The structure of compounds in *Bambusa tuldoids* munro

Table S12 Chemical compositions in *Physochlaina infundibularis* kuang

No.	Compound	No.	Compound
**S1**	Scopolamine	**S11**	6, 7-dimethoxycoumarin
**S2**	Anisodamine	**S12**	3-O-Methylquercetin
**S3**	Hyoscyamine	**S13**	Isoquercitrin
**S4**	Atropine	**S14**	Kaempferol 7-O-glucoside
**S5**	Scopoline	**S15**	Syringarenol
**S6**	choline	**S16**	Protocatechuic acid
**S7**	Scopoletin	**S17**	Methyl 4-hydroxymethylbenzoate
**S8**	Scopolin	**S18**	2-hydroxybenzoic acid
**S9**	Fabiatrin	**S19**	4-hydroxy-benzoic acid
**S10**	Umbellifero		

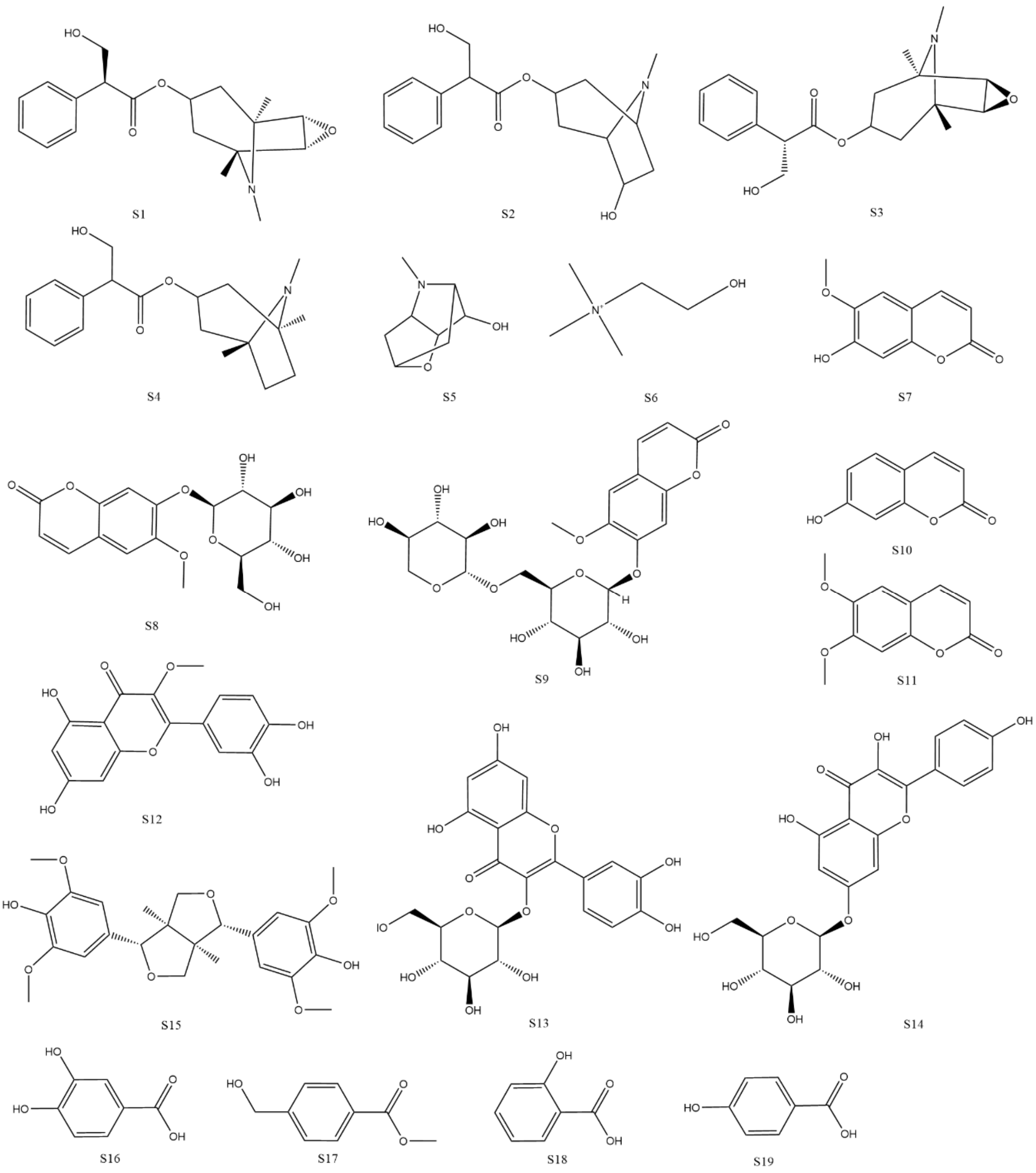

Figure S13. The structure of compounds in *Physochlaina infundibularis* kuang

Table S13 The main compound in *Ganoderma lucidum*

Cpd	Name
1	n-Butyl ganoderate H (n-butyl 12β-acetoxy-3β-hydroxy-7,11,15, tetraoxo-5α-lanost-8-en-Cheng et al. 2010-oate)
2	Butyl ganoderate A
3	Butyl ganoderate B
4	Ganoderic acid α (12β-acetoxy-3β, 15β-dihydroxy-7,11,23-trioxo-5α-lanosta-8-en-26-oic acid)
5	Ganolucidic acid A
6	Methyl ganolucidate A (methyl 15α-hydroxy-3,11,23-trioxo-5α-lanost-8-en-26-oate)
7	Ganolucidic acid B
8	Methyl ganolucidate B
9	Ganoderic acid A (7β, 15α-dihydroxy-3,11,23-trioxo-5α-lanost-8-en-26-oic acid)
10	Methyl ganoderate A (methyl 7β, 15α-dihydroxy-3,11,23-trioxo-5α-lanost-8-en-26-oate)
11	Methyl ganoderate A (methyl 7β, 15α-dihydroxy-3,11,23-trioxo-5α-lanost-8-en-26-oate)
12	Methyl ganoderate B (methyl 3β, 7β-dihydroxy-11,15,23-trioxo-5α-lanost-8-en-26-oate)
13	Ganoderic acid C (3β, 7β, 15α-trihydroxy-11,23-dioxo-5α-lanost-8-en-26-oic acid)
14	Methyl ganoderate C
15	Ganoderic acid D (7β-hydroxy-3,11,15,23-tetraoxo-5α-lanost-8-en-26-oic acid)
16	Methyl ganoderate D
17	Methyl ganoderate C 2 (methyl 3β, 7β, 15α-trihydroxy-11,23-dioxo-5α-lanost-8-en-26-oate)
18	Methyl ganoderate K
19	Compound B_8
20	Compound B_9
21	3β-Oxo-formyl-7β, 12β-dihydroxy-5α-lanost-11,15,23-trioxo-8-en(E)-26-oic acid
22	Ganoderic acid B_8
23	Ganoderic acid C_1
24	12β-Acetoxy-3,7,11,15,23-pentaoxo-5α-lanosta-8-en-26-oic acid ethyl ester
25	3β, 7β-Dihydroxy-12β-acetoxy-11,15,23-trioxo-5α-lanosta-8-en-26-oic acid methyl ester
26	3β-Hydroxy-7,11,12,15,23-pentaoxolanost-8-en-26-oic acid
27	Ganoderic acid Df (7β, 11β-dihydroxy-3,15,23-trioxo-5α-lanosta-8-

	en-26-oic acid)
**28**	Ganoderic acid H
**29**	Ganoderic acid F (12β-acetoxy-3,7,11,15-pentaoxo-5α-lanost-8-en-26-oic acid)
**30**	Ganoderic acid E (3,7,11,15,23-pentaoxo-5α-lanost-8-en-26-oic acid)
**31**	Ganoderic acid K
**32**	Ganoderic acid AM_1
**33**	Ganoderic acid J
**34**	Ganoderic acid C2 (3β, 7β, 15α-trihydroxy-11,23-dioxo-5α-lanosta-8-en-26-oic acid)
**35**	Ganoderic acid G (3β, 7β, 15β-trihydroxy-11,15,23-trioxo-5α-lanosta-8-en-26-oic acid)
**36**	7β, 12β-Dihydroxy-3,11,15,23-tetraoxo-5α-lanosta-8-en-26-oic acid
**37**	12β-Hydroxy-3,7,11,15,23-pentaoxo-5α-lanosta-8-en-26-oic acid
**38**	Methyl ganoderate D (methyl 3β, 7β, 15α-trihydroxy-11,23-dioxo-5α-lanost-8-en-26-oate)
**39**	Methyl ganoderate E (methyl 3β, 7β, 15α-trihydroxy-11,23-dioxo-5α-lanost-8-en-26-oate)
**40**	Methyl ganoderate F (methyl 12β-acetoxy-3,7,11,15,23-pentaoxo-5α-lanost-8-en-26-oate)
**41**	Methyl ganoderate H (methyl 3β-hydroxy-12β-acetoxy-7,11,15,23-tetraoxo-5α-lanost-8-en-26-oate)
**42**	Methyl ganoderate G (methyl 3β, 7β, 12β-trihydroxy-11,15,23-trioxo-5α-lanost-8-en-26-oate)
**43**	Compound C_5
**44**	Compound C_6
**45**	Ganoderic acid AP3 (15α, 20ξ-dihydroxy-3,7,11,23-tetraoxolanost-8-en-26-oic acid)
**46**	23-Dihydroganoderic acid I (3β, 7β, 20,23ξ-tetrahydroxy-11,15-dioxolanosta-8-en-26-oic acid)
**47**	23-Dihydroganoderic acid N (7β, 20,23ξ-trihydroxy-3,11,15-trioxolanosta-8-en-26-oic acid)
**48**	20-Hydroxylganoderic acid G
**49**	Ganoderic acid I
**50**	Methyl ganoderate I
**51**	Methyl ganoderate AP (methyl 12β,15α, 20-trihydroxy-3,7,11,23- tetraoxo-5α-lanost-8-en-26-oate)
**52**	Methyl ganoderate N (Methyl 7β, 20-dihydroxy-3,11,15,23-tetraoxo-5α-lanost-8-en-26-oate)
**53**	Methyl ganoderate M (methyl 7β, 12α-dihydroxy-3,11,15,23-tetraoxo-5α-lanost-8-en-26-oate)
**54**	3-O-Acetylganoderic acid B (3β-acetoxy-7β-hydroxy-11,15,23-trioxolanost-8-en-26-oic acid)

**55**	3-O-Acetylganoderic acid K (3*β*-acetyloxy-15*α*-hydroxy-7,11,23-trioxolanost-8-en-26-oic acid)
**56**	Ethyl 3-O-acetylganoderate B
**57**	Ethyl ganoderate J
**58**	Leucocontextin S
**59**	Leucocontextin T
**60**	Leucocontextin U
**61**	3*α*-Carboxyacetoxy-24-methylene-23-oxolanost-8-en-26-oic acid
**62**	3*α*-Carboxyacetoxy-24-methyl-23-oxolanost-8-en-26-oic acid
**63**	Ganodernoid E
**64**	Ganodernoid G
**65**	3*β*,7*β*,15*α*,24-Tetrahydroxy-11,23-dioxo-lanost-8-en-26-oic Acid
**66**	24*S*,25*R*-dihydroxy-3,7-dioxo-8-en-5a-lanosta-26-ol
**67**	(24S)-3-oxo-7*α*,15*α*,24,25-tetrahydroxylanosta-8-ene
**68**	Ganodaustralic acid A
**69**	Ganoderense H
**70**	Ganoderense I
**71**	Ganoderiol D
**72**	7*β*,15*β*,28-Trihydroxy-3,11,23-trioxolanost-8-en-26-oate
**73**	3*β*,7*β*,15*β*-Trihydroxy-11,23-dioxolanost-8-en-26-oate
**74**	15*β*,20-Dihydroxy-3,7,11,23-tetraoxolanost-8-en-26-oate
**75**	Ganohainanic acid A
**76**	Acetyl ganohainanic acid A
**77**	Ganohainanic acid B
**78**	Ganohainanic acid C
**79**	Ganohainanic acid D
**80**	Acetyl ganohainanic acid C
**81**	Lucidumol A ((24*S*)-24,25-dihydroxylanost-8-ene-3,7-dione)
**82**	Ganoderiol C (7*α*-ethoxy-24,25,26-trihydroxy-5*α*-lanost-8-en-3-one)
**83**	Ganoderiol D (24,25,26-trihydroxy-5*α*-lanost-8-en-3,7-dione)
**84**	Ganoderiol G (24,25,26-trihydroxy-7*α*-methoxy-5*α*-lanost-8-en-3-one)
**85**	Applanaic acids A
**86**	(20*E*)-3*β*,7*β*,15*β*-Trihydroxy-11,23-dioxolanost-8,20(22)-dien-26-oate
**87**	(20*E*)-3*β*,15*β*-Dihydroxy-7,11,23-trioxolanost-8,20(22)-dien-26-oate
**88**	(20*E*)-3*β*,7*β*-Dihydroxy-11,23-dioxolanost-8,20(22)-dien-26-oicacid
**89**	(20*E*)-15*β*-Hydroxy-3,7,11,23-tetraoxolanost-8,20(22)-dien-26-oic acid
**90**	(20*E*)-7*β*,15*β*,28-Trihydroxy-3,11,23-trioxolanost-8,20(22)-dien-26-oate
**91**	3*β*-hydroxy-12*β*-acetoxy-7,11,15,23-tetraoxolanosta-8,20*E*(22)-dien-26-oic acid methyl ester
**92**	15*α*-hydroxy-3,11, 23-trioxolanosta-8,20*E*(22)-dien-26-oic acid methyl ester

**93**	12*β*-Acetoxy-3*β*-hydroxy-7,11,15,23-tetraoxo-lanost-8,20*E*-diene-26-oic acid
**94**	23-Dihydroganoderenic acid D (7*β*, 23ξ-dihydroxy-3,11,15-trioxolanosta-8,20*E*(22)-dien-26-oic acid)
**95**	Methyl ganoderenate D (7*β*-hydroxy-3,11,15,23-tetraoxolanosta-8,20*E*(22)-dien-26-oic acid methyl ester)
**96**	Ganoderenic acid A ((20*E*)-7*β*, 15*α*-dihydroxy-3,11,23-trioxo-5*α*-lanost-8,20-dien-26-oic acid)
**97**	Ganoderenic acid B ((20*E*)-3*β*, 7*β*-dihydroxy-11,15,23-trioxo-5*α*-lanost-8,20-dien-26-oic acid)
**98**	Ganoderenic acid C ((20*E*)-3*β*, 7*β*, 15*α*-trihydroxy-11,23-dioxo-5*α*-lanost-8,20-dien-26-oic acid)
**99**	Ganoderenic acid D ((20*E*)-7*β*-hydroxy-3,11,15,23-tetraoxo-5*α*-lanost-8,20-dien-26-oic acid)
**100**	12*β*-Acetoxy-7*β*-hydroxy-3,11,15,23-tetraoxo-5*α*-lanosta-8,20-dien-26-oic acid
**101**	Ganoderenic acid F (3,7,11,15,23-pentaoxo-5*α*-lanosta-8,20*E*-dien-26-oic acid)
**102**	Ganoderenic acid G (15*α*-hydroxy-3,7,11,23-tetraoxo-5*α*-lanosta-8,20*E*-dien-26-oic acid)
**103**	Methy ganoderenate H (methyl 3*β*-hydroxy-7,11,15,23-tetraoxo-5*α*-lanosta-8,20*E*-dien-26-oate)
**104**	Methyl ganoderenate I (3*β*, 15*α*-dihydroxy-7,11,23-trioxo-5*α*-lanosta-8,20*E*-dien-26-oate)
**105**	Ganoderenic acid H
**106**	12*β*-Acetoxy-3*β*, 7*β*-dihydroxy-11,15,23-trioxo-5*α*-lanosta-8,20-dien-26-oic acid
**107**	Gibbosic acid I
**108**	Gibbosic acid J
**109**	Gibbosic acid K
**110**	Methyl ganoderenate E (7*β*, 12*β*-dihydroxy-3,11,15,23-tetraoxo-5*α*-lanosta-8,20*E*-dien-26-oate)
**111**	12*β*-acetoxy-3,7,11,15,23-pentaoxo-lanost-8,20*E*-dien-26-oic acid
**112**	Ganoderic acid GS-1 (7*β*-hydroxy-3,11,15-trioxolanosta-8,24(*E*)-dien-26-oic acid)
**113**	Ganoderic acid GS-2 (7*β*,15*α*-dihydroxy-3,11-dioxolanosta-8,24(*E*)-dien-26-oic acid)
**114**	Ganoderic acid GS-3 (12*β*-acetoxy-3*β*, 7*β*-dihydroxy-11,15-dioxo-lanosta-8,24(*E*)-dien-26-oic acid
**115**	Ganoderic acid AP 2 (12*β*, 15*α*-diacetoxy-3*β*-hydroxy-11-oxolanost-8,24(*E*)-dien-26-oic acid)
**116**	23*S*-Hydroxy-3,7,11,15-tetraoxolanost-8,24*E*-diene-26-oic acid
**117**	7-Oxoganoderic acid Z (3*β*-hydroxy-7-oxo-5*α*-lanosta-8,24(*E*)-dien-26-oic acid)
**118**	Ganoderic acid LM2 ((23*S*) 7*β*,-dihydroxy-3,11,15-trioxo-5*α*-lanosta-8,24-dien-26-oic acid)

**119**	Lucialdehyde B ((24*E*)-3,7-dioxo-5α-lanosta-8,24-dien-26-al)
**120**	Lucialdehyde C ((24*E*)-3*β*-hydroxy-7-oxo-5*α*-lanosta-8,24-dien-26-al)
**121**	Ganoderic acid γ ((23*S*)-7*β*, 15α, 23-trihydroxy-3,11-dioxolanosta-8,24(*E*)-diene-26-oic acid)
**122**	Ganoderic acid δ ((23*S*)-7*α*, 15*α*, 23-trihydroxy-3,11-dioxolanosta-8,24(*E*)-diene-26-oic acid)
**123**	Ganoderic acid ε ((23*S*)-3*β*, 7*β*, 23-trihydroxy-11,15-dioxolanosta-8,24(*E*)-diene-26-oic acid)
**124**	Ganoderic acid ζ ((23*S*)-3*β*, 23-dihydroxy-7,11,15-trioxolanosta-8,24(*E*)-diene-26-oic acid)
**125**	Ganoderic acid η ((23*S*)-3*β*, 7*β*, 12*β*, 23-tetrahydroxy-11,15-dioxolanosta-8,24(*E*)-diene-26-oic acid)
**126**	Ganoderic acid θ ((23*S*)-3*β*, 12*β*, 23-trihydroxy-7,11,15-trioxolanosta-8,24(*E*)-diene-26-oic acid)
**127**	Ganoderic acid *β* (3*β*, 7*β*-dihydroxy-11,15-dioxolanosta-8,24(*E*)-dien-26-oic acid)
**128**	Ganolucidic acid E (15*α*-hydroxy-3,11-dioxo-5*α*-lanosta-8,24*E*-dien-26-oic acid)
**129**	Ganoderal B (7*α*-hydroxy-3-oxo-5*α*-lanosta-8,24*E*-dien-26-al)
**130**	Ganoderic acid Ma (3*α*, 7*α*-diacetoxy-15*α*-hydroxy-5α-lanost-8,24*E*-dien-26-oic acid)
**131**	Lucialdehyde D (3,7,11-trioxo-5*α*-lanosta-8,24-diene-26-al)
**132**	Ganoderone A (5*α*-lanosta-8,24-diene-26-hydroxy-3,7-dione)
**133**	ganoderic acid Mi (3α-acetoxy-15α-hydroxy-7*α*-methoxy-5*α*-lanost-8,24*E*-dien-26-oic acid)
**134**	11α-Hydroxy-3,7-dioxo-5*α*-lanosta-8,24(*E*)-dien-26-oic acid
**135**	11*β*-Hydroxy-3,7-dioxo-5*α*-lanosta-8,24(*E*)-dien-26-oic acid
**136**	Lucidadiol (5α-lanosta-8,24-dien-3*β*, 26-dihydroxy-7-one)
**137**	Lucidal (5*α*-lanosta-8,24*E*-dien-3*β*-hydroxy-7-on-26-al)
**138**	Ganoderic acid DM (3,7-dioxo-8,24(*E*)-dien-lanosta-26-oic acid)
**139**	Ganoderic acid V
**140**	Ganolucidic acid γa (3*β*, 7*β*, 15α, 23-tetrahydroxy-11-oxo-5*α*-lanosta-8,24-dien-26-oic acid)
**141**	Ganolucidate F (3*β*, 15*α*, 23-trihydroxy-11-oxo-5*α*-lanosta-8,24-dien-26-oic acid)
**142**	Lucialdehyde E (7*β*, 15*α*-dihydroxy-3,11-dioxo-5*α*-lanosta-8,24-dien-26-al)
**143**	Ganolucidic acid D
**144**	Ganoderic acid W
**145**	Ganoderic acid Mb (3*α*, 15*α*, 22-triacetoxy-7*α*-hydroxy-5α-lanost-8,24*E*-dien-26-oic acid)
**146**	Ganoderic acid Mc (3α, 7*α*, 22-triacetoxy-15*α*-hydroxy-5*α*-lanost-8, 24*E*-dien-26-oic acid)
**147**	Ganoderic acid Md (3*α*, 22-diacetoxy-7*α*-methoxy-5*α*-lanost-8, 24*E*-dien-26-oic acid)

**148**	Ganoderic acid Mg (3α, 22-diacetoxy-15α-hydroxy-7α-methoxy-5α-lanost-8,24*E*-dien-26-oic acid)
**149**	Ganoderic acid Mh (3α, 22-diacetoxy-7α, 15α-dihydroxy-5α-lanost-8,24*E*-dien-26-oic acid)
**150**	Ganoderic acid Mj (22-acetoxy-3α-hydroxy-7α-methoxy-5α-lanost-8,24*E*-dien-26-oic acid)
**151**	3α, 22β-Diacetoxy-7α-hydroxyl-5α-lanost-8,24*E*-dien-26-oic acid
**152**	Ganorbiformin B
**153**	Ganorbiformin C
**154**	Ganorbiformin D
**155**	Ganorbiformin E
**156**	Ganorbiformin F
**157**	Ganoderic acid O ((22*S*, 24*E*)-3α, 15α, 22-triacetoxy-7α-hydroxy-5α-lanosta-7,24-dien-26-oic acid)
**158**	7-O-Methylganoderic acid O ((22*S*, 24*E*)-3α, 15α, 22-triacetoxy-7α-methoxy-5α-lanosta-8,24-dien-26-oic acid)
**159**	(22*S*,24*E*)-15α,22-Diacetoxy-3-oxolanosta-8,24-dien-26-oic acid
**160**	(22*S*,24*E*)-3β,15α,22-Triacetoxylanosta-8,24-dien-26-oic acid
**161**	(22*S*,24*E*)-15α,22-Diacetoxy-3β-hydroxylanosta-8,24-dien-26-oicacid
**162**	(24*E*)-15α-hydroxy-3-oxolanosta-8,24-dien-26-oic acid.
**163**	(24*E*)-7α,15α-dihydroxy-3-oxolanosta-8,24-dien-26-oic acid.
**164**	(24*E*)-3β-Acetoxy-7α-hydroxylanosta-8,24-dien-26-oic acid
**165**	(24*E*)-3β,15α-Diacetoxy-7α-hydroxylanosta-8,24-dien-26-oicacid
**166**	(22*S*,24*E*)-7α-Hydroxy-3β,15α,22-triacetoxylanosta-8,24-dien-26-oic acid
**167**	Leucocontextin W
**168**	Ganoleucoin Y
**169**	Ganoleucoin Z
**170**	Hainanaldehyde A
**171**	Sinensoic acid (3,26-dihydroxy-5-lanosta-8,24*E*-dien-21-oic acid)
**172**	Tsugarioside B (3α-acetoxy-5α-lanosta-8,24-diene-21-O-β-D- xyloside)
**173**	Tsugaric acid A (3α-acetoxy-5α-lanosta-8,24-dien-21-oic acid)
**174**	Ganosinoside A (3-oxo-5α-lanosta-8,24-dien-21-oic acid ester β-D- glucoside)
**175**	Tsugarioside A (3α-acetoxy-5α-lanosta-8,24-dien-21-oic acid esterβ-D-glucoside)
**176**	3-Oxo-5α-lanosta-8,24-dien-21-oic acid
**177**	3β-Hydroxy-5α-lanosta-8,24-dien-21-oic acid
**178**	Leucocontextins A
**179**	Leucocontextins B
**180**	Lleucocontextins C
**181**	Leucocontextins D
**182**	Leucocontextins J

**183**	Leucocontextins K
**184**	Lleucocontextins L
**185**	Lleucocontextins M
**186**	Lleucocontextins N
**187**	Lleucocontextins R
**188**	Lleucocontextins E
**189**	Leucocontextins H
**190**	Leucocontextins F
**191**	Leucocontextins G
**192**	Lleucocontextins I
**193**	Leucocontextins Q
**194**	(24*Z*)-3-oxo-7*α*,15*α*,27-trihydroxylanosta-8,24-diene.
**195**	3-oxo-7*α*,15*α*,26,27-tetrahydroxylanosta-8,24-diene.
**196**	Hainanic acid A
**197**	Hainanic acid B
**198**	Ganodrol A
**199**	21-hydroxy-3,7-dioxo-8,24*E*-dien-5a-lanosta-26-ol
**200**	(20*S*,24*E*)-21-chloro-15*β*,20,29-trihydroxy-3,7,11-trioxolanosta-8, 24-dien-26-oic acid.
**201**	(20*S*,24*E*)-15*β*,20,29-trihydroxy-3,7,11-trioxolanosta-8,24-dien-26-oic acid.
**202**	(20*S*,24*E*)-15*β*,20-dihydroxy-3,7,11-trioxolanosta-8,24-dien-26-oicacid
**203**	(20*S*,24*E*)-7,11-dioxo-3*β*,15*β*,20,29-tetrahydroxylanosta-8,24-dien-26-oic acid
**204**	Ganoderiol E (3*β*,26,27-trihydroxy-5*α*-lanosta-8,24-dien-7-one)
**205**	Ganoderiol I (15*α*,26,27-trihydroxy-5*α*-lanosta-8,24-dien-3-one)
**206**	Ganoderiol J (26,27-dihydroxy-5*α*-lanosta-8,24-dien-3,7-dione)
**207**	3*β*, 15*α*-Diacetoxylanosta-8,24-dien-26-oic acid
**208**	Ganoderic acid U5
**209**	Ganoderic acid U6
**210**	(22*S*,24*E*)-22-acetoxy-3,7-dioxolanosta-8,24-dien-26-oic acid
**211**	(22*S*,24*E*)-3*α*,22-diacetoxy-7-oxolanosta-8,24-dien-26-oic acid
**212**	3*α*-acetoxy-5*α*-lanosta-8,24(24′)-dien-21-oic acid
**213**	(24*E*)-7*α*-acetoxy-15*α*-hydroxy-3-oxolanosta-8,24-dien-26-oic acid
**214**	Colossolactone A
**215**	Ganocolossusins H
**216**	Ganoderic acid U4
**217**	3b,7b-dihydroxy-11-oxo-8,24E-dien-5a-lanosta-26-ol
**218**	(24*Z*)-7*α*-hydroxy-3-oxo-lanosta-8,24-dien-26-oic acid
**219**	(24*E*)-7*α*-Methoxy-3-oxolanosta-8,24-dien-26-oic acid
**220**	(22*S*,24*E*)-3*β*,22-Diacetoxy-7*α*-methoxylanosta-8,24-dien-26-oicacid
**221**	(22*S*,24*E*)-7*α*-Methoxy-3*β*,15*α*,22-triacetoxylanosta-8,24-dien-26-oic acid

**222**	(24*E*)-3*β*-Acetoxy-15*α*-hydroxy-7*α*-methoxylanosta-8,24-dien-26-oic acid
**223**	(24*S*)-7*α*,24-dihydroxy-3-oxo-lanosta-8,25-diene,
**224**	(24*S*)-3,7-dioxo-24-hydroxylanasta-8,25-diene
**225**	(24*S*)-24-hydroxy-3-oxo-lanasta-7,9(11),25-triene
**226**	(24*S*)-3*β*,24-dihydroxylanasta-7,9(11),25-triene
**227**	Tsugaric acid C ((24*R*,*S*)-3*α*-acetoxy-24-hydroxy-5*α*-lanosta-8,25-dien-21-oic acid)
**228**	3*β*-hydroxy-7,22-dioxo-5*α*-lanosta-8,24-dien-21-oic acid
**229**	Applanaic acid B
**230**	(17*Z*)-3*β*,7*β*,15*β*-Trihydroxy-11,23-dioxolanost-8,17(20)-dien-26-oate
**231**	Ganodernoid D
**232**	Tsugaric acid B (3*α*-acetoxy-16*α*-hydroxy-24ξ-methyl-5*α*-lanosta-8,25-dien-21-oic acid)
**233**	3-Epipachymic acid (3*α*-acetoxy-16*α*-hydroxy-24-methylene-5*α*-lanost-8-en-21-oic acid)
**235**	Epoxyganoderiol A (24*S*, 25*S*-epoxy-7*α*, 26-dihydroxy-5*α*-lanost-8-en-3- one)
**236**	Ganoderone C (5*α*-lanosta-8-ene-24,25-epoxy-26-hydroxy-3,7-dione)
**237**	Colossolactone B
**238**	Ganodermalactones X
**239**	Colossolactone I ((22*S*)-3-*β*-hydroxylanosta-8,24-dien-26,22-olide)
**240**	Colossolactone II ((22*S*)-1,3-*β*-dihydroxylanosta-8,24-dien-26,22-olide)
**241**	Offiffifficimalonic acid L
**242**	Offiffifficimalonic acid M
**243**	Offiffifficimalonic acid N
**244**	Offiffifficimalonic acid O
**245**	Spiroganocalitone A
**246**	Spiroganocalitone B
**247**	Spiroganocalitone C
**248**	Spiroganocalitone D
**249**	Ganotropic Acid
**250**	(24*E*)-20,21-epoxy-15*β*-hydroxy-3,7,11-trioxolanasta-8,24-dien-26-oic acid.
**251**	Applanaic acids C
**252**	Furanoganoderic acid (21,23-epoxy-15*α*-hydroxy-3,7,11-trioxo-5*α*-lanosta-8,20(21),22-trien-26-oic acid)
**253**	Ganodermacetal (methyl 7*β*, 15*α*-isopropylide-nedioxy-3*β*-hydroxy-11,23-dioxo-5*α*-lanost-8-en-26-oate)
**254**	Methyl ganoderate A acetonide (methyl 7*β*, 15*α*-isopropylidenedioxy-3,11,23-trioxo-5*α*-lanost-8-en-26-oate)
**255**	Leucocontextin V
**256**	Austrolactone (I)

257	Ganodermalactones W
258	Colossolactone III ((22*S*)-3β, 19-epoxy-lanosta-8,24-dien-26,22-olide)
259	Butyl lucidenate N
260	Butyl lucidenate A
261	20(21)-Dehydrolucidenic acid N (3β, 7β-dihydroxy-11,15-dioxo-25,26,27- trinorlanosta-8,20-dien-24-oic acid)
262	20-Hydroxylucidenic acid A (7β, 20ξ-dihydroxy-3,11,15-trioxo-25,26,27-trinorlanost-8-en-24-oic acid)
263	Methyl lucidenate D (methyl 12β-acetoxy-3,7,11,15-tetraoxo-5α-lanost-8-en-24-oate)
264	20(21)-Dehydrolucidenic acid A (7β-Hydroxy-3,11,15-trioxo-25,26,27-trisnorlanosta-8,20(21)-dien-24-oic acid)
265	Methyl 20(21)-dehydrolucidenate A (methyl 7β-hydroxy-3,11,15-trioxo-25,26,27-trisnorlanosta-8,20(21)-dien-24-oate)
266	Lucidenic acid N (3,7-dihydroxy-4,4,14-trimethyl-11,15-dioxo-5-chol-8-en-24-oic acid)
267	Lucidenic acid D (12β-acetoxy-4,4,14α-trimethyl-3,7,11,15-tetraoxo-5α-chol-8-en-24-oic acid)
268	Methyl lucidenate E
269	Methyl lucidenate F
270	Ethyl lucidenates A (ethyl 7β-hydroxy-4,4,14α-trimethyl-3,11,15-trioxo-5α-chol-8-en-24-oate)
271	3β-Oxo-formyl-7β, 12β-dihydroxy-4,4,14α-trimethyl-5α-chol-11,15-dioxo-8-en(E)-24-oic acid
272	Lucidenic acid A 273(7β-hydroxy-4,4,14α-trimethyl-3,11,15-trioxo-5α-chol-8-en-24-oic acid)
273	Lucidenic acid B (7β, 12-dihydroxy-4,4,14α-trimethyl-3,11,15-trioxo-5α-chol-8-en-24-oic acid)
274	Lucidenic acid C (3β, 7β, 12-trihydroxy-4,4,14α-trimethyl-11,15-dioxo-5α-chol-8-en-24-oic acid)
275	4,4,14α-Trimethyl-3,7-dioxo-5α-chol-8-en-24-oic acid
276	(3β, 7β-dihydroxy-12β-acetoxy-25,26,27-trinor-11,15-dioxo dioxolanost-8-en-24-oic acid)
277	Methyl lucidenate P
278	Methyl lucidenate Q (methyl-7β, 15α-dihydroxy-25,26,27-trinor-3,11-dioxolanost-8-en-24-oate)
279	3β-Hydroxy-4,4,14-trimethyl-7,11,15-trioxochol-8-en-24-oic acid
280	Methyl lucidenate D2
281	Methyl lucidenate E2
282	Methyl lucidenate N (methyl 3β, 7β-dihydroxy-4,4,14α-trimethyl-11,15-dioxo-5α-chol-8-en-24-oate)

**283**	t-Butyl lucidenate B (t-butyl 7*β*, 12*β*-dihydroxy-4,4,14*α*-trimethyl-3,11,15-trioxo-5*α*-chol-8-en-24-oate)
**284**	Methyl lucidenate A
**285**	Lucidenic acid D2
**286**	20-Hydroxylucidenic acid D 2 ((20ξ)-12*β*-acetoxy-20-hydroxy-3,7,11,15-tet-raoxo-25,26,27-trisnorlanost-8-en-24-oic acid)
**287**	20-Hydroxylucidenic acid F ((20ξ)-20-hydroxy-3,7,11,15-tetraoxo-25,26,27-trisnorlanost-8-en-24-oic acid)
**288**	20-Hydroxylucidenic acid E 2 (12β-acetoxy-3*β*-hydroxy-7,11,15-trioxo-25,26,27-trisnorlanost-8-en-24-oic acid)
**289**	20-Hydroxylucidenic acid N ((20ξ)-3*β*, 7*β*, 20-trihydroxy-11,15-dioxo-25,26,27-trisnorlanost-8-en-24-oic acid)
**290**	20-Hydroxylucidenic acid P ((20ξ)- 12*β*-acetoxy-3*β*, 7*β*, 20-trihydroxy-11,15-dioxo-25,26,27-trisnorlanost-8-en-24-oic acid)
**291**	Lucidenic acid F
**292**	Methyl lucidenate C
**293**	Lucidenic acid E2
**294**	Luciderie acid A
**295**	Methyl lucidenate H (methyl 3*β*, 7*β*-dihydroxy-4*α*-hydroxymethyl-4*β*, 14α-dimethyl-11,15-dioxo-5*α*-chol-8-en-24-oate)
**296**	Methyl lucidenate I (3*β*-hydroxy-4*α*-hydroxymethyl-4*β*, 14*α*-dimethyl-7,11,15-trioxo-5*α*-chol-8-en-24-oate)
**297**	Methyl lucidenate J (3*β*, 12*β*-dihydroxy-4*α*-hydroxymethyl-4*β*, 14*α*-dimethyl-7,11,15-trioxo-5*α*-chol-8-en-24-oate)
**298**	Methyl lucidenate Ha
**299**	Ganodernoid C
**300**	Lucidone A
**301**	Lucidenol
**302**	Ganosineniol A
**303**	Ganolucidoid A.
**304**	Ganolucidoid B
**305**	3*β*-acetyloxy-lucidone H
**306**	Ganoluciduone A
**307**	Ganosporelactone A
**308**	Ganosporelactone B
**309**	Ganolactone (7*β*-hydroxy-3,11,15-trioxo-lanosta-8-en-24→20s lactone)
**310**	Ganoderlactone B
**311**	Ganoderlactone C
**312**	Ganoderlactone D
**313**	Ganoderlactone E

314	Ganolactone B (3β, 7β-dihydroxy-11,15-dioxolanosta-8-en-24→20 lactone)
315	8α, 9α-Epoxy-4,4,14α-trimethyl-3,7,11,15,20-pentaoxo-5α-pregnane
316	3β, 7β-Dihydroxy-11,15,23-trioxolanost-8,16-dien-26-oic acid
317	3β, 7β-Dihydroxy-11,15,23-trioxolanost-8,16-dien-26-oic acid methyl ester
318	12β-Acetoxy-3β, 7β-dihydroxy-11,15,23-trioxolanost-8,16-dien-26-oic acid
319	20-carbinol-7β,15α-dihydroxy-4,4,14α-trimethyl-3,11-dioxo-5α-pregn-8,16-dien
320	Ganoderal A
321	Ganoderol A
322	15-Hydroxy-ganoderic acid S (15α-hydroxy-3-oxo-5α-lanosta-7,9(11),24(*E*)-trien-26-oic acid)
323	3α, 16α-Dihydroxylanosta-7,9(11),24-trien-21-oic acid
324	3α, 16α, 26-Trihydroxylanosta-7,9(11),24-trien-21-oic acid
325	Ganoderic acid S1
326	Ganoderic acid SZ (3-oxo-lanosta-7,9(11),24(*Z*)-trien-26-oic acid)
327	5α-Lanosta-7,9(11),24-triene-15α-26-dihydroxy-3-one
328	Ganoderic acid Me (3α, 15α-diacetoxy-5α-lanost-7,9(11),24E-trien-26-oic acid)
329	Ganoderic acid Mf (3α-acetoxy-15α-hydroxy-5α-lanost-7,9(11),24E-trien-26-oic acid)
330	Ganodermenonol (26-hydroxy-5α-lanosta-7,9(11),24-trien-3-one)
331	Ganodermadiol (5α-lanosta-7,9(11),24-triene-3β, 26-diol)
332	Ganodermatriol (5α-lanosta-7,9(11),24-triene-3β, 26,27-triol)
333	Ganodermic acid S (lanosta-7,9(11),24-trien-3β, 15α-diacetoxy-26-oic acid)
334	Carnosodione (26,27-dihydroxylanosta-7,9(11),24-trien-3,16-dione)
335	Canoderol B ((24*E*)-5α-lanosta-7,9(11),24-trien-3,26-diol)
336	Ganoderic acid Mk (3α, 22-diacetoxy-15α-hydroxy-5α-lanost-7,9(11),24*E*-trien-26-oic acid)
337	Ganoderiol B (15α, 26,27-trihydroxy-5α-lanosta-7,9(11),24-trien-3- one)
338	Ganoderic acid T ((22*S*, 24*E*)-3α, 15α, 22-triacetoxy-5α-lanosta-7,9,(11),24-trien-26-oic acid)
339	Ganoderic acid S ((22*S*, 24*E*)-22-acetoxy-3α-hydroxy-5α-lanosta-7,9(11),24-trien-26-oic acid)
340	Ganoderic acid R ((22*S*, 24*E*)-3α, 22-diacetoxy-5α-lanosta-7,9,(11),24-trien-26-oic acid)
341	Ganorbiformin G
342	Lanosta-7,9(11),24-trien-3β, 15α, 22β-triacetoxy-26-oic acid
343	Lanosta-7,9(11),24-trien-15α-acetoxy-3α-hydroxy-23-oxo-26-oic acid
344	Lanosta-7,9(11),24-trien-3α, 15α-diacetoxy-23-oxo-26-oic acid
345	Lanosta-7,9(11),24-trien-3α, 15α-hydroxy-23-oxo-26-oic acid
346	Lanosta-7,9(11),24-trien-3α-acetoxy-15α, 22β-dihydroxy-26-oic acid
347	Ganodermic acid T-N (3β-hydroxy-15α-acetoxy-lanosta-7,9(11),24-trien-26-oic acid)

**348**	Ganodermic acid T-O (3β-acetoxy-15α-hydroxy-lanosta-7,9(11),24-trien-26-oic acid)
**349**	Ganodermic acid T-Q (3β-oxo-15α-acetoxy-lanosta-7,9(11),24-trien-26-oic acid)
**350**	Compound 10
**351**	Ganoderic acid P ((22S, 24E)-15α, 22-diacetoxy-3α-hydroxy-5α-lanosta-7,9(11),24-trien-26-oic acid)
**352**	Ganoderic acid Q ((22S, 24E)-3α, 22-diacetoxy-15α-hydroxy-5α-lanosta-7,9(11),24-trien-26-oic acid)
**353**	Ganoderic acid Jc (15α, 23-dihydroxy-3-oxo-5α-lanosta-7,9(11),24-trien-26-oic acid)
**354**	Ganodermatetraol (3β, 15α, 26,27-tetrahydroxy-5α-lanosta-7,9(11),24-triene)
**355**	Lucialdehyde A ((24E)-3β-hydroxy-5α-lanosta-7,9(11),24-trien-26-al)
**356**	Ganoderiol F (26,27-dihydroxy-5α-lanosta-7,9(11),24-trien-3-one)
**357**	26,27-Dihydroxy-5α-lanosta-7,9(11),24-triene-3,22-dione
**358**	26-Hydroxy-5α-lanosta-7,9(11),24-triene-3,22-dione
**359**	Ganodermic acid P1 (lanosta-7,9(11),24-trien-3α, 22β-diacetoxy-15α-hydroxy-26-oic acid)
**360**	Ganodermic acid P 2 (lanosta-7,9(11),24-trien-15α, 22β-diacetoxy-3β-hydroxy-26-oic acid)
**361**	Lanosta-7,9(11),24-trien-3β, 15α, 22-triacetoxy-26-oic acid
**362**	16α-Hydroxy-3-oxolanosta-7,9(11),24-trien-21-oic acid
**363**	(20S,24E)-15β,20-dihydroxy-3-oxolanosta-7,9(11),24-trien-26-oic acid.
**364**	Ganodaustralic acid D
**365**	15α-acetoxy-5α-lanosta-7,9(11),24-trien-3β,26-dio
**366**	3β-hydroxy-15α-acetoxy-5α-lanosta-7,9(11),24-trien-26-al
**367**	3α, 15α, 22α-Trihydroxylanosta-7,9(11),24-trien-26-oic acid
**368**	3β, 15α, 22β-Trihydroxylanosta-7,9(11),24-trien-26-oic acid
**369**	3α, 15α-Diacetoxy-22α-hydroxylanosta-7,9(11),24-trien-26-oic acid
**370**	3β, 15α-Diacetoxy-22α-hydroxylanosta-7,9(11),24-trien-26-oic acid
**371**	22β-Acetoxy-3α, 15α-dihydroxylanosta-7,9(11),24-trien-26-oic acid
**372**	22β-Acetoxy-3β, 15α-dihydroxylanosta-7,9(11),24-trien-26-oic acid
**373**	Ganoderic acid TR
**374**	Ganodermic acid Ja (lanosta-7,9(11),24-trien-3α, 15α-dihydroxy-26-oic acid)
**375**	Ganodermic acid Jb (lanosta-7,9(11),24-trien-3β, 15α-dihydroxy-26-oic acid)
**376**	Ganodermic acid R (lanosta-7,9(11),24-trien-3α, 15α-diacetoxy-26-oic acid)
**377**	Ganoderic acid U1
**378**	15α, 26-Dihydroxy-5α-lanosta-7,9,24(E)-trien-3-one
**379**	3β-Hydroxy-5α-lanosta-7,9,24(E)-trien-26-oic acid
**380**	(24E)-3β-Acetoxylanosta-7,9(11),24-trien-26-oic acid

**381**	(22*S*,24*E*)-3*β*,22-Diacetoxylanosta-7,9(11),24-trien-26-oic acid (15)
**382**	(22*S*,24*E*)-22-Acetoxy-3*β*-hydroxylanosta-7,9(11),24-trien-26-oic acid
**383**	Ganodaustralic acid G
**384**	Ganodaustralic acid F
**385**	Ganodrol B
**386**	3-oxo-25-methoxy-24,26-dihydroxy-lanosta-7,9(11)-diene
**387**	Ganoweberianones B
**388**	(24*S*, 25*R*)-25-methoxylanosta -7,9(11)-dien-3*β*,24,26-triol
**389**	Ganoderiol a triacetate (3*β*, 24,26-triacetoxy-5*α*-lanosta-7, 9(11)-dien-25-ol)
**390**	Lucidumol B ((24*S*)-lanosta-7,9(11)-diene-3*β*, 24,25-triol)
**391**	Ganodermanontiol (24,25,26-trihydroxy-5*α*-lanosta-7,9(11)-dien-3-one)
**392**	Ganoderiol A (5*α*-lanosta-7,9(11)-dien-3*β*, 24,25,26-tetraol)
**393**	Ganodermanondiol
**394**	Ganoderic acid X (3*α*-hydroxy-15*α*-acetoxy-lanosta-7,9(11),24-trien-26-oic acid)
**395**	Ganosinensin A (ganodermanontriol 26-O-{(2*Z*, 5*E*, 9*E*)-2-[2-(2,5-dihydroxyphenyl)-2-oxo-ethylidene]-11-hydroxy-6,10-dimethylundeca-5,9-dienate})
**396**	Ganosinensin B (ganodermanontriol 24-O- {(2*Z*, 5*E*, 9*E*)-2-[2-(2,5-dihydroxyphenyl)-2-oxo-ethylidene]-11-hydroxy-6,10-dimethylundeca-5,9-dienate)
**397**	Ganosinensin C (ganodermanontriol 24-O- {(2*Z*, 5*E*, 9*E*)-2-[2-(2,5-dihydroxyphenyl)ethylidene]-11-hydroxy-6,10-dimethylundeca-5,9-dien-ate)
**398**	Epoxyganoderiol B (24*S*, 25*S*-epoxy-26-hydroxy-5*α*-lanosta-7,9(11)-diene-3-one)
**399**	Epoxyganoderiol C (24*S*, 25*S*-epoxy-5*α*-lanosta-7,9(11)-diene-3*β*, 26-diol)
**400**	26,27-dihydroxy-24,25-epoxylanosta-7,9(11)-dien-3-one
**401**	Gibbosicolids C
**402**	Applanoxidic acid A (15*α*-hydroxy-7*α*, 8*α*-epoxy-3,12,23-trioxo-5*α*-lanosta-9(11),20*E*-dien-26-oic acid)
**403**	Applanoxidic acid B (3*β*-hydroxy-7*α*, 8*α*-epoxy-12,15,23-trioxo-5*α*-lanosta-9(11),20*E*-dien-26-oic acid)
**404**	Applanoxidic acid F (7*α*, 8*α*-epoxy-3,12,15,23-tetraoxo-5*α*-lanosta-9(11),20*E*-dien-26-oic acid)
**405**	Applanoxidic acid C (20-hydroxy-7*α*, 8*α*-epoxy-3,12,15,23-tetraoxo-5*α*-lanosta-9(11),16-dien-26-oic acid)
**406**	Applanoxidic acid D (3*β*, 20-dihydroxy-7*α*, 8*α*-epoxy-12,15,23-trioxo-5*α*-lanosta-9(11),16-dien-26-oic acid)
**407**	Applanlactone A
**408**	Applanoic acid F
**409**	Applanoic acid E

**410**	Lucialdehyde A ((24*E*)-3*β*-hydroxy-5*α*-lanosta-7,9(11),24-trien-26-al)
**411**	Ganoderal A
**412**	Ganoderol A
**413**	Ganoderic acid TR
**414**	Ganodermic acid Ja (lanosta-7,9(11),24-trien-3*α*, 15*α*-dihydroxy-26-oic acid)
**415**	Ganodermic acid Jb (lanosta-7,9(11),24-trien-3*β*, 15*α*-dihydroxy-26-oic acid)
**416**	Ganodermic acid R (lanosta-7,9(11),24-trien-3*α*, 15*α*-diacetoxy-26-oic acid)
**417**	15*α*-Hydroxy-3-oxo-5*α*-lanosta-7,9,24(*E*)-triene-26-oic acid
**418**	15*α*, 26-Dihydroxy-5*α*-lanosta-7,9,24(*E*)-trien-3-one
**419**	3*β*-Hydroxy-5*α*-lanosta-7,9,24(*E*)-trien-26-oic acid
**420**	Ganodermenonol (26-hydroxy-5*α*-lanosta-7,9(11),24-trien-3-one)
**421**	Ganoluciduone B
**422**	Lanosta-7,9(11),24-trien-3-one15,26-dihydroxy
**423**	Lanosta-7,9(11),24-trien-26-oic,3-hydroxy
**424**	Ganoderic acid Y ((24*E*)-3-ol-5*α*-lanosta-7,9(11),24-trien-26-oic acid)
**425**	4,4,14*α*-Trimethyl-5*α*-chol-7,9(11)-dien-3-oxo-24-oic acid
**426**	Ganoderic acid Jd (15*α*-hydroxy-3-oxo-5*α*-lano-sta-7,9(11)-dien-24-oic acid)
**427**	Applanone A
**428**	Applanone B
**429**	16,17-dehydroapplanone E
**430**	Applanone D
**431**	Applanone E
**432**	Fornicatin C ((3*β*)-3-hydroxy-18(13→12*β*)-abeo-lanosta-13(17),24-dien- 18-oic acid)
**433**	Applanoxidic acid G (15*β*, 20-dihydroxy-7*α*, 8*α*-epoxy-3,12,23-trioxo-5*α*-lanosta-9(11),16-dien-26-oic acid)
**434**	Applanoxidic acid H (3*β*, 12*α*, 20-trihydroxy-7*α*, 8*α*-epoxydioxo-5*α*-lanosta-9(11),16-dien-26-oic acid)
**435**	Methyl applaniate B
**436**	8*β*, 9*α*-Dihydroganoderic acid J
**437**	Methyl 8*β*, 9*α*-dihydroganoderate J
**438**	Ganosporeric acid A (3,7,11,12,15,23-hexaoxo-5*α*-lanosta-8-en-26-oic acid)
**439**	24ξ-Methyl-5*α*-lanosta-25-one
**440**	16*α*,20-dihydroxy-3,23-dioxo-5*α*-lanosta-6,8-dien-26-oic acid
**441**	Piptolinic acids F
**442**	Piptolinic acids I
**443**	Piptolinic acids G
**444**	Fornicatin C ((3*β*)-3-hydroxy-18(13→12*β*)-abeo-lanosta-13(17),24-dien- 18-oic acid)
**445**	Ganorbiformin A
**446**	Gibbosic acid M

**447**	Ganoderol X.
**448**	8*β*, 9*α*-Dihydroganoderic acid J
**449**	Methyl 8*β*, 9*α*-dihydroganoderate J
**450**	Ganosporeric acid A (3,7,11,12,15,23-hexaoxo-5*α*-lanosta-8-en-26-oic acid)
**451**	24ξ-Methyl-5*α*-lanosta-25-one
**452**	(20*E*)-15*β*-Hydroxy-3,7,11,23-tetraoxolanost-20(22)-en-26-oate
**453**	(20*E*)-15*β*-Hydroxy-3,7,11,23-tetraoxolanost-20(22)-en-26-oate
**454**	Fornicatins D
**455**	Fornicatins E
**456**	Fornicatins F
**457**	Fornicatin B (7*β*-hydroxy-11-oxo-3,4-seco-25,26,27-trinorlanosta-4(28),8-dien-3,24-dioic acid)
**458**	Fornicatin G (7*β*-hydroxy-11-oxo-3,4-seco-25,26,27-trinorlanosta-4(28),8-dien-24-oic-3-acetyl ester)
**459**	Fornicatin A (4, 7*β*-epoxy-28-hydroxy-11-oxo-3,4-seco-25,26,27-trinorlanosta-8-en-3,24-dioic acid)
**460**	Fornicatin H (4, 7*β*-epoxy-28-hydroxy-11-oxo-3,4-seco-25,26,27-trinorlansta-8-en-3,24-diester)
**461**	Australic acid ((20*Z*, 23*R*, 25*R*)-15*α*-acetyl-7*α*, 8*α*-epoxy-12-oxo-3,4-seco-5*α*-lanosta-4(28),9,20(22)-trien-23,26-olid-3-oic acid)
**462**	Gibbosicolid E
**463**	Gibbosicolid F
**464**	Gibbosicolid G
**465**	Cochlates A
**466**	Cochlates B
**467**	Colossolactone E
**468**	Colossolactone F
**469**	Colossolactone G
**470**	Colossolactone G
**471**	Colossolactone VIII ((22*S*, 23*R*)-A,B-dihomo-19-nor-15-*β*-acetoxy-23-hydroxy-4-oxa-3-oxolanosta-1,8,19,24-tetraen-26,22-olide)
**472**	Ganosinensic acid A
**473**	Methyl ganosinensate A
**475**	Ganocolossusins E
**476**	Ganocolossusins F
**477**	Gganorbifate H
**478**	Ganorbifate F

**479**	Ganorbifate G
**480**	Colossolactone VI ((22*R*)-3,4-seco-19,22-diacetoxy-4-hydroxylanosta-7,9(11),24(*Z*)-trien-3,26-dioic acid 3-methyl ester)
**481**	Colossolactone V ((22*R*)-3,4-seco-19,22-diacetoxy-4-hydroxylanosta-8,24(*Z*)-dien-3,26-dioic acid 3-methyl-ester)
**482**	Colossolactone VII ((22*S*)-3,4-seco-19-acetoxy-4-hydroxylanosta-8,24-3dien-26,22-olide 3-methyl ester)
**483**	Ganodermalactones U
**484**	Ganocolossusins D
**485**	Ganorbifates A
**486**	Ganorbifate C
**487**	Ganorbifate D
**488**	Ganorbifate E
**489**	Ganorbifates B
**490**	Ganorbifate I
**491**	Colossolactone IV ((22*S*)-A,B-dihomo-19-nor-4-oxalanosta-8,24-dien-26,22-olide)
**492**	Colossolactone C
**493**	Ganocolossusins B
**494**	Ganocolossusins C
**495**	Ganodermalactones V
**496**	Ganocolossusins A
**497**	Ganosidone A
**498**	Ganosinensic acid B

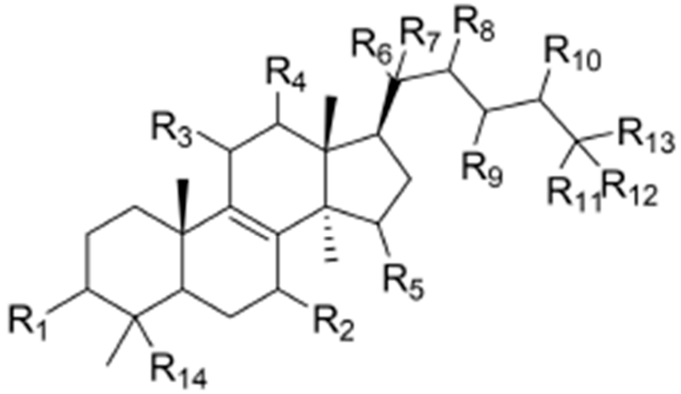

Figure S14 Chemical structure of compound 1-231

Table S14 Chemical Structures of compounds 1-231

Cpd	R1	R2	R3	R4	R5	R6	R7	R8	R9	R10	R11	R12	R13	R14
**1**	β-OH	=O	=O	β-*O*-Ac	=O	α-CH_3	H	H	H	H	COOBu	H	CH_3	β-CH_3
**2**	=O	β-OH	=O	H	α-OH	α-CH_3	H	H	H	H	COOBu	H	CH_3	β-CH_3
**3**	β-OH	β-OH	=O	H	=O	α-CH_3	H	H	H	H	COOBu	H	CH_3	β-CH_3
**4**	β-OH	=O	=O	β-*O*-Ac	α-OH	α-CH_3	H	H	H	H	COOH	H	CH_3	β-CH_3
**5**	=O	H	=O	H	α-OH	β-CH_3	H	H	H	H	COOH	H	CH_3	β-CH_3
**6**	=O	H	=O	H	α-OH	β-CH_3	H	H	H	H	$COOCH_3$	H	CH_3	β-CH_3
**7**	β-OH	H	=O	H	α-OH	β-CH_3	H	H	H	H	COOH	H	CH_3	β-CH_3
**8**	β-OH	H	=O	H	α-OH	β-CH_3	H	H	H	H	$COOCH_3$	H	CH_3	β-CH_3
**9**	=O	β-OH	=O	H	α-OH	β-CH_3	H	H	H	H	COOH	H	CH_3	β-CH_3
**10**	=O	β-OH	=O	H	α-OH	β-CH_3	H	H	H	H	$COOCH_3$	H	CH_3	β-CH_3
**11**	β-OH	β-OH	=O	H	=O	β-CH_3	H	H	H	H	COOH	H	CH_3	β-CH_3
**12**	β-OH	β-OH	=O	H	=O	β-CH_3	H	H	H	H	$COOCH_3$	H	CH_3	β-CH_3
**13**	β-OH	β-OH	=O	H	α-OH	β-CH_3	H	H	H	H	COOH	H	CH_3	β-CH_3
**14**	β-OH	β-OH	=O	H	α-OH	β-CH_3	H	H	H	H	$COOCH_3$	H	CH_3	β-CH_3
**15**	=O	β-OH	=O	H	=O	β-CH_3	H	H	H	H	COOH	H	CH_3	β-CH_3
**16**	=O	β-OH	=O	H	=O	β-CH_3	H	H	H	H	$COOCH_3$	H	CH_3	β-CH_3
**17**	β-OH	β-OH	=O	H	α-OH	α-CH_3	H	H	H	H	$COOCH_3$	H	CH_3	β-CH_3
**18**	β-OH	=O	=O	H	α-OH	α-CH_3	H	H	H	H	$COOCH_3$	H	CH_3	β-CH_3
**19**	=O	α-OH	=O	H	α-OH	α-CH_3	H	H	H	H	$COOCH_3$	H	CH_3	β-CH_3
**20**	β-OH	α-OH	=O	H	α-OH	α-CH_3	H	H	H	H	$COOCH_3$	H	CH_3	β-CH_3
**21**	*O*-CHO	β-OH	=O	β-OH	=O	β-CH_3	H	H	H	H	COOH	H	CH_3	β-CH_3
**22**	=O	α-OH	=O	H	α-OH	α-CH_3	H	H	H	H	COOH	H	CH_3	β-CH_3
**23**	=O	β-OH	=O	H	=O	α-CH_3	H	H	H	H	COOH	H	CH_3	β-CH_3
**24**	=O	=O	=O	β-*O*-Ac	=O	α-CH_3	H	H	H	H	COOEt	H	CH_3	β-CH_3
**25**	β-OH	β-OH	=O	β-*O*-Ac	=O	α-CH_3	H	H	H	H	$COOCH_3$	H	CH_3	β-CH_3
**26**	β-OH	=O	=O	=O	=O	α-CH_3	H	H	H	H	COOH	H	CH_3	β-CH_3
**27**	=O	β-OH	β-OH	H	=O	α-CH_3	H	H	H	H	COOH	H	CH_3	β-CH_3
**28**	β-OH	=O	=O	β-*O*-Ac	=O	β-CH_3	H	H	H	H	COOH	H	CH_3	β-CH_3
**29**	=O	=O	=O	β-*O*-Ac	=O	β-CH_3	H	H	H	H	COOH	H	CH_3	β-CH_3
**30**	=O	=O	=O	H	=O	β-CH_3	H	H	H	H	COOH	H	CH_3	β-CH_3
**31**	β-OH	β-OH	=O	β-*O*-Ac	=O	α-CH_3	H	H	H	H	COOH	H	CH_3	β-CH_3
**32**	β-OH	=O	=O	H	=O	α-CH_3	H	H	H	H	COOH	H	CH_3	β-CH_3
**33**	=O	=O	=O	H	α-OH	α-CH_3	H	H	H	H	COOH	H	CH_3	β-CH_3
**34**	β-OH	β-OH	=O	H	α-OH	α-CH_3	H	H	H	H	COOH	H	CH_3	β-CH_3
**35**	β-OH	β-OH	=O	β-OH	=O	β-CH_3	H	H	H	H	COOH	H	CH_3	β-CH_3
**36**	=O	β-OH	=O	β-OH	=O	α-CH_3	H	H	H	H	COOH	H	CH_3	β-CH_3
**37**	=O	=O	=O	β-OH	=O	α-CH_3	H	H	H	H	COOH	H	CH_3	β-CH_3
**38**	β-OH	β-OH	=O	H	α-OH	α-CH_3	H	H	H	H	$COOCH_3$	H	CH_3	β-CH_3
**39**	=O	=O	=O	H	=O	α-CH_3	H	H	H	H	$COOCH_3$	H	CH_3	β-CH_3
**40**	=O	=O	=O	β-*O*-Ac	=O	α-CH_3	H	H	H	H	$COOCH_3$	H	CH_3	β-CH_3
**41**	β-OH	=O	=O	β-*O*-Ac	=O	α-CH_3	H	H	H	H	$COOCH_3$	H	CH_3	β-CH_3
**42**	β-OH	β-OH	=O	β-OH	=O	α-CH_3	H	H	H	H	$COOCH_3$	H	CH_3	β-CH_3
**43**	=O	β-OH	=O	OH	=O	α-CH_3	H	H	H	H	$COOCH_3$	H	CH_3	β-CH_3
**44**	β-OH	=O	=O	OH	=O	α-CH_3	H	H	H	H	$COOCH_3$	H	CH_3	β-CH_3
**45**	=O	=O	=O	H	α-OH	β-CH_3	ξ-OH	H	=O	H	COOH	H	CH_3	β-CH_3
**46**	β-OH	β-OH	=O	H	=O	α-CH_3	β-OH	H	ξ-OH	H	ξ-COOH	H	CH_3	β-CH_3
**47**	=O	β-OH	=O	H	=O	α-CH_3	β-OH	H	ξ-OH	H	ξ-COOH	H	CH_3	β-CH_3
**48**	β-OH	β-OH	=O	β-OH	=O	β-CH_3	β-OH	H	=O	H	COOH	H	CH_3	β-CH_3
**49**	β-OH	β-OH	=O	=O	=O	α-CH_3	ξ-OH	H	=O	H	COOH	H	CH_3	β-CH_3
**50**	β-OH	β-OH	=O	H	=O	α-CH_3	β-OH	H	H	H	$COOCH_3$	H	CH_3	β-CH_3
**51**	=O	=O	=O	β-OH	α-OH	α-CH_3	β-OH	H	H	H	$COOCH_3$	H	CH_3	β-CH_3
**52**	=O	β-OH	=O	H	=O	α-CH_3	β-OH	H	H	H	$COOCH_3$	H	CH_3	β-CH_3
**53**	=O	β-OH	=O	α-OH	=O	α-CH_3	β-OH	H	H	H	$COOCH_3$	H	CH_3	β-CH_3
**54**	β-*O*-Ac	β-OH	=O	H	=O	α-CH_3	H	H	H	H	COOH	H	CH_3	β-CH_3
**55**	β-*O*-Ac	=O	=O	H	α-OH	α-CH_3	H	H	H	H	COOH	H	CH_3	β-CH_3

Cpd	R1	R2	R3	R4	R5	R6	R7	R8	R9	R10	R11	R12	R13	R14
**56**	β-*O*-Ac	β-OH	=O	H	=O	α-CH$_3$	H	H	H	H	COOEt	H	CH$_3$	β-CH$_3$
**57**	=O	=O	=O	H	α-OH	α-CH$_3$	H	H	H	H	COOEt	H	CH$_3$	β-CH$_3$
**58**	=O	=O	α-OH	H	H	α-CH$_3$	H	H	H	α-OH	CH$_2$OH	β-OH	CH$_3$	β-CH$_3$
**59**	=O	β-OH	=O	H	H	α-CH$_3$	H	H	H	α-OH	CH$_2$OH	β-OH	CH$_3$	β-CH$_3$
**60**	β-OH	=O	α-OH	H	H	α-CH$_3$	H	H	H	α-OH	CH$_2$OH	β-OH	CH$_3$	β-CH$_3$
**61**	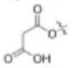	H	H	H	H	α-CH$_3$	H	H	=O	=CH$_2$	COOH	H	CH$_3$	β-CH$_3$
**62**		H	H	H	H	α-CH$_3$	H	H	=O	α-CH$_3$	COOH	H	CH$_3$	β-CH$_3$
**63**	β-OH	β-OH	=O	α-OH	=O	α-CH$_3$	H	H	=O	H	COOCH$_3$	H	CH$_3$	β-CH$_3$
**64**	β-OH	β-OH	=O	α-OH	=O	α-CH$_3$	H	H	=O	H	COOH	H	CH$_3$	β-CH$_3$
**65**	β-OH	β-OH	=O	H	α-OH	α-CH$_3$	H	H	=O	α-OH	COOH	H	CH$_3$	α-CH$_3$
**66**	=O	=O	=O	H	H	α-CH$_3$	H	H	H	α-OH	CH$_2$OH	α-OH	CH$_3$	α-CH$_3$
**67**	=O	α-OH	H	H	β-OH	α-CH$_3$	H	H	H	α-OH	CH$_3$	H	CH$_3$	α-CH$_3$
**68**	β-OH	=O	=O	=O	β-OH	α-CH$_3$	β-OH	H	=O	H	COOH	H	CH$_3$	α-CH$_3$
**69**	β-OH	H	=O	H	H	α-CH$_3$	H	H	H	α-OH	CH$_2$OH	OH	CH$_3$	α-CH$_3$
**70**	β-OH	=O	α-OH	=O	H	α-CH$_3$	H	H	H	α-OH	CH$_3$	OH	CH$_3$	α-CH$_3$
**71**	=O	=O	=O	H	H	α-CH$_3$	H	H	H	OH	CH$_2$OH	OCH$_3$	CH$_3$	α-CH$_3$
**72**	=O	β-OH	=O	H	β-OH	α-CH$_3$	H	H	=O	H	COOCH$_3$	H	CH$_3$	α-CH$_2$OH
**73**	β-OH	β-OH	=O	H	β-OH	α-CH$_3$	H	H	=O	H	COOCH$_3$	H	CH$_3$	β-CH$_3$
**74**	=O	=O	=O	H	β-OH	α-CH$_3$	OH	H	=O	H	COOCH$_3$	H	CH$_3$	β-CH$_3$
**75**	=O	=O	H	H	β-OH	α-CH$_3$	H	H	H	H	COOH	H	CH$_3$	α-CH$_2$OH
**76**	=O	=O	H	H	β-OH	α-CH$_3$	H	H	H	H	COOH	H	CH$_3$	α-CH$_2$OAc
**77**	=O	=O	=O	H	β-OH	α-CH$_3$	H	H	H	H	COOH	H	CH$_3$	H
**78**	=O	=O	H	H	H	α-CH$_3$	H	H	H	α-OH	CH$_3$	OH	CH$_3$	β-CH$_3$
**78**	=O	α-O-Et	H	H	H	β-CH$_3$	H	H	H	ξ-OH	CH$_2$OH	OH	CH$_3$	β-CH$_3$
**80**	=O	=O	H	H	H	β-CH$_3$	H	H	H	ξ-OH	CH$_2$OH	OH	CH$_3$	β-CH$_3$
**81**	=O	α-O-CH$_3$	H	H	H	β-CH$_3$	H	H	H	ξ-OH	CH$_2$OH	OH	CH$_3$	β-CH$_3$
**82**	β-OH	=O	H	H	H	β-CH$_3$	H	H	H	ξ-OH	CH$_2$OH	OH	CH$_3$	β-CH$_3$
**83**	β-OH	=O	H	H	H	α-CH$_3$	H	H	H	α-OH	CH$_3$	OH	CH$_3$	β-CH$_3$
**84**	=O	=O	=O	H	H	α-CH$_3$	H	H	H	α-OH	CH$_3$	OH	CH$_3$	β-CH$_3$
**85**	=O	=O	=O	H	=O	CH$_3$	-	H, Δ^{20,22}	=O	H	COOH	H	CH$_3$	β-CH$_3$
**85**	β-OH	β-OH	=O	H	β-OH	CH$_3$	-	H, Δ^{20,22}	=O	H	COOCH$_3$	H	CH$_3$	β-CH$_3$
**86**	β-OH	=O	=O	H	β-OH	CH$_3$	-	H, Δ^{20,22}	=O	H	COOCH$_3$	H	CH$_3$	β-CH$_3$
**87**	β-OH	β-OH	=O	H	H	CH$_3$	-	H, Δ^{20,22}	=O	H	COOH	H	CH$_3$	β-CH$_3$
**88**	=O	=O	=O	H	H	CH$_3$	-	H, Δ^{20,22}	=O	H	COOH	H	CH$_3$	β-CH$_3$
**89**	=O	β-OH	=O	H	β-OH	CH$_3$	-	H, Δ^{20,22}	=O	H	COOCH$_3$	H	CH$_3$	α-CH$_2$OH
**90**	β-OH	=O	=O	β-*O*-Ac	=O	CH$_3$	-	H, Δ^{20,22}	=O	H	COOCH$_3$	H	CH$_3$	β-CH$_3$
**91**	=O	H	=O	H	α-OH	CH$_3$	-	H, Δ^{20,22}	=O	H	COOCH$_3$	H	CH$_3$	β-CH$_3$
**92**	β-OH	=O	=O	β-*O*-Ac	=O	CH$_3$	-	H, Δ^{20,22}	=O	H	COOH	H	CH$_3$	β-CH$_3$
**93**	=O	β-OH	=O	H	=O	CH$_3$	-	H, Δ^{20,22}	ξ-OH	H	ξ-COOH	H	CH$_3$	β-CH$_3$
**94**	=O	β-OH	=O	H	=O	CH$_3$	-	H, Δ^{20,22}	=O	H	ξ-COOCH$_3$	H	CH$_3$	β-CH$_3$
**95**	=O	β-OH	=O	H	α-OH	CH$_3$	-	H, Δ^{20,22}	=O	H	COOH	H	CH$_3$	β-CH$_3$
**96**	β-OH	β-OH	=O	H	=O	CH$_3$	-	H, Δ^{20,22}	=O	H	COOH	H	CH$_3$	β-CH$_3$
**97**	β-OH	β-OH	=O	H	α-OH	CH$_3$	-	H, Δ^{20,22}	=O	H	COOH	H	CH$_3$	β-CH$_3$
**98**	=O	β-OH	=O	H	=O	CH$_3$	-	H, Δ^{20,22}	=O	H	COOH	H	CH$_3$	β-CH$_3$
**99**	=O	β-OH	=O	β-*O*-Ac	=O	CH$_3$	-	H, Δ^{20,22}	=O	H	COOH	H	CH$_3$	β-CH$_3$
**100**	=O	=O	=O	H	=O	CH$_3$	-	H, Δ^{20,22}	=O	H	COOH	H	CH$_3$	β-CH$_3$
**101**	=O	=O	=O	H	α-OH	CH$_3$	-	H, Δ^{20,22}	=O	H	COOH	H	CH$_3$	β-CH$_3$
**102**	β-OH	=O	=O	H	=O	CH$_3$	-	H, Δ^{20,22}	=O	H	COCH$_3$	H	CH$_3$	β-CH$_3$
**103**	β-OH	=O	=O	H	α-OH	CH$_3$	-	H, Δ^{20,22}	=O	H	COCH$_3$	H	CH$_3$	β-CH$_3$
**104**	β-OH	=O	=O	H	=O	CH$_3$	-	H, Δ^{20,22}	=O	H	COOH	H	CH$_3$	β-CH$_3$
**105**	β-OH	β-OH	=O	β-*O*-Ac	=O	CH$_3$	-	H, Δ^{20,22}	=O	H	COOH	H	CH$_3$	β-CH$_3$
**106**	=O	=O	=O	α-OH	β-OH	CH$_3$	-	H, Δ^{20,22}	=O	H	COOH	H	CH$_3$	β-CH$_3$
**107**	=O	=O	OH	α-OH	β-OH	CH$_3$	-	H, Δ^{20,22}	=O	H	COOH	H	CH$_3$	β-CH$_3$
**108**	=O	=O	H	α-OH	β-OH	CH$_3$	-	H, Δ^{20,22}	=O	H	COOH	H	CH$_3$	β-CH$_3$
**109**	=O	OH	=O	OH	=O	CH$_3$	-	H, Δ^{20,22}	=O	H	COOCH$_3$	H	CH$_3$	β-CH$_3$
**110**	=O	=O	=O	β-*O*-Ac	=O	CH$_3$	-	H, Δ^{20,22}	=O	H	COOH	H	CH$_3$	β-CH$_3$

Cpd	R1	R2	R3	R4	R5	R6	R7	R8	R9	R10	R11	R12	R13	R14
**111**	=O	β-OH	=O	H	=O	α-CH_3	H	H	H	H, $\Delta^{24,25}$	COOH	-	CH_3	β-CH_3
**112**	=O	β-OH	=O	H	α-OH	α-CH_3	H	H	H	H, $\Delta^{24,25}$	COOH	-	CH_3	β-CH_3
**113**	β-OH	β-OH	=O	β-*O*-Ac	=O	α-CH_3	H	H	H	H, $\Delta^{24,25}$	COOH	-	CH_3	β-CH_3
**114**	β-OH	H	=O	β-*O*-Ac	α-*O*-Ac	α-CH_3	H	H	H	H, $\Delta^{24,25}$	COOH	-	CH_3	β-CH_3
**115**	=O	=O	=O	H	=O	α-CH_3	H	H	β-OH	H, $\Delta^{24,25}$	COOH	-	CH_3	β-CH_3
**116**	β-OH	=O	H	H	H	β-CH_3	H	H	H	H, $\Delta^{24,25}$	COOH	-	CH_3	β-CH_3
**117**	=O	OH	=O	H	=O	α-CH_3	H	H	OH	H, $\Delta^{24,25}$	COOH	-	CH_3	β-CH_3
**118**	=O	=O	H	H	H	α-CH_3	H	H	H	H, $\Delta^{24,25}$	CHO	-	CH_3	β-CH_3
**119**	β-OH	=O	H	H	H	α-CH_3	H	H	H	H, $\Delta^{24,25}$	CHO	-	CH_3	β-CH_3
**120**	=O	β-OH	=O	H	α-OH	α-CH_3	H	H	β-OH	H, $\Delta^{24,25}$	COOH	-	CH_3	β-CH_3
**121**	=O	α-OH	=O	H	α-OH	α-CH_3	H	H	β-OH	H, $\Delta^{24,25}$	COOH	-	CH_3	β-CH_3
**122**	β-OH	β-OH	=O	H	=O	α-CH_3	H	H	β-OH	H, $\Delta^{24,25}$	COOH	-	CH_3	β-CH_3
**123**	β-OH	=O	=O	H	=O	α-CH_3	H	H	β-OH	H, $\Delta^{24,25}$	COOH	-	CH_3	β-CH_3
**124**	β-OH	β-OH	=O	β-OH	=O	α-CH_3	H	H	β-OH	H, $\Delta^{24,25}$	COOH	-	CH_3	β-CH_3
**125**	β-OH	=O	=O	β-OH	=O	α-CH_3	H	H	β-OH	H, $\Delta^{24,25}$	COOH	-	CH_3	β-CH_3
**126**	β-OH	β-OH	=O	H	=O	α-CH_3	H	H	H	H, $\Delta^{24,25}$	COOH	-	CH_3	β-CH_3
**127**	=O	H	=O	H	α-OH	β-CH_3	H	H	H	H, $\Delta^{24,25}$	COOH	-	CH_3	β-CH_3
**128**	=O	α-OH	H	H	H	β-CH_3	H	H	H	H, $\Delta^{24,25}$	CHO	-	CH_3	β-CH_3
**129**	α-*O*-Ac	α-*O*-Ac	H	H	α-OH	β-CH_3	H	H	H	H, $\Delta^{24,25}$	COOH	-	CH_3	β-CH_3
**130**	=O	=O	=O	H	H	α-CH_3	H	H	H	H, $\Delta^{24,25}$	CHO	-	CH_3	β-CH_3
**131**	=O	=O	H	H	H	α-CH_3	H	H	H	H, $\Delta^{24,25}$	CH_2OH	-	CH_3	β-CH_3
**132**	α-*O*-Ac	α-*O*-CH_3	H	H	α-OH	β-CH_3	H	H	H	H, $\Delta^{24,25}$	COOH	-	CH_3	β-CH_3
**133**	=O	=O	α-OH	H	H	α-CH_3	H	H	H	H, $\Delta^{24,25}$	COOH	-	CH_3	β-CH_3
**134**	=O	=O	β-OH	H	H	α-CH_3	H	H	H	H, $\Delta^{24,25}$	COOH	-	CH_3	β-CH_3
**135**	β-OH	=O	H	H	H	α-CH_3	H	H	H	H, $\Delta^{24,25}$	CH_2OH	-	CH_3	β-CH_3
**136**	β-OH	=O	H	H	H	α-CH_3	H	H	H	H, $\Delta^{24,25}$	CHO	-	CH_3	β-CH_3
**137**	=O	=O	H	H	H	α-CH_3	H	H	H	H, $\Delta^{24,25}$	COOH	-	CH_3	β-CH_3
**138**	=O	α-OH	H	H	α-O-Ac	α-CH_3	H	H	H	H, $\Delta^{24,25}$	COOH	-	CH_3	β-CH_3
**139**	β-OH	β-OH	=O	H	α-OH	α-CH_3	H	H	β-OH	H, $\Delta^{24,25}$	COOH	-	CH_3	β-CH_3
**140**	β-OH	H	=O	H	α-OH	α-CH_3	H	H	β-OH	H, $\Delta^{24,25}$	COOH	-	CH_3	β-CH_3
**141**	=O	β-OH	=O	H	α-OH	α-CH_3	H	H	H	H, $\Delta^{24,25}$	CHO	-	CH_3	β-CH_3
**142**	=O	H	=O	H	α-OH	α-CH_3	H	H	β-OH	H, $\Delta^{24,25}$	COOH	-	CH_3	β-CH_3
**143**	α-*O*-Ac	α-OH	H	H	α-*O*-Ac	β-CH_3	H	H	H	H, $\Delta^{24,25}$	COOH	-	CH_3	β-CH_3
**144**	α-*O*-Ac	α-OH	H	H	α-*O*-Ac	β-CH_3	H	ξ-*O*-Ac	H	H, $\Delta^{24,25}$	COOH	-	CH_3	β-CH_3
**145**	α-*O*-Ac	α-*O*-Ac	H	H	α-OH	β-CH_3	H	ξ-*O*-Ac	H	H, $\Delta^{24,25}$	COOH	-	CH_3	β-CH_3
**146**	α-*O*-Ac	α-*O*-CH_3	H	H	H	β-CH_3	H	ξ-*O*-Ac	H	H, $\Delta^{24,25}$	COOH	-	CH_3	β-CH_3
**147**	α-*O*-Ac	α-*O*-CH_3	H	H	α-OH	β-CH_3	H	ξ-*O*-Ac	H	H, $\Delta^{24,25}$	COOH	-	CH_3	β-CH_3
**148**	α-*O*-Ac	α-OH	H	H	α-OH	β-CH_3	H	ξ-*O*-Ac	H	H, $\Delta^{24,25}$	COOH	-	CH_3	β-CH_3
**149**	α-OH	α-*O*-CH_3	H	H	H	β-CH_3	H	ξ-*O*-Ac	H	H, $\Delta^{24,25}$	COOH	-	CH_3	β-CH_3
**150**	α-*O*-Ac	α-OH	H	H	H	α-CH_3	H	β-*O*-Ac	H	H, $\Delta^{24,25}$	COOH	-	CH_3	β-CH_3
**151**	β-*O*-Ac	=O	H	H	H	α-CH_3	H	β-*O*-Ac	H	H, $\Delta^{24,25}$	COOH	-	CH_3	β-CH_3
**152**	β-OH	=O	H	H	H	α-CH_3	H	β-*O*-Ac	H	H, $\Delta^{24,25}$	COOH	-	CH_3	β-CH_3
**153**	=O	α-OH	H	H	α-*O*-Ac	α-CH_3	H	β-*O*-Ac	H	H, $\Delta^{24,25}$	COOH	-	CH_3	β-CH_3
**154**	=O	α-OH	H	H	H	α-CH_3	H	β-*O*-Ac	H	H, $\Delta^{24,25}$	COOH	-	CH_3	β-CH_3
**155**	=O	α-*O*-CH_3	H	H	H	α-CH_3	H	β-*O*-Ac	H	H, $\Delta^{24,25}$	COOH	-	CH_3	β-CH_3
**156**	α-*O*-Ac	α-OH	H	H	α-*O*-Ac	α-CH_3	H	β-*O*-Ac	H	H, $\Delta^{24,25}$	COOH	-	CH_3	β-CH_3
**157**	α-*O*-Ac	α-*O*-CH_3	H	H	α-*O*-Ac	α-CH_3	H	β-*O*-Ac	H	H, $\Delta^{24,25}$	COOH	-	CH_3	β-CH_3
**158**	=O	H	H	H	α-*O*-Ac	α-CH_3	H	β-*O*-Ac	H	H, $\Delta^{24,25}$	COOH	-	CH_3	β-CH_3
**159**	β-*O*-Ac	H	H	H	α-*O*-Ac	α-CH_3	H	β-*O*-Ac	H	H, $\Delta^{24,25}$	COOH	-	CH_3	β-CH_3
**160**	β-OH	H	H	H	α-*O*-Ac	α-CH_3	H	β-*O*-Ac	H	H, $\Delta^{24,25}$	COOH	-	CH_3	β-CH_3
**161**	=O	H	H	H	α-OH	α-CH_3	H	H	H	H, $\Delta^{24,25}$	COOH	-	CH_3	β-CH_3
**162**	=O	α-OH	H	H	α-OH	α-CH_3	H	H	H	H, $\Delta^{24,25}$	COOH	-	CH_3	β-CH_3
**163**	β-*O*-Ac	α-OH	H	H	H	α-CH_3	H	H	H	H, $\Delta^{24,25}$	COOH	-	CH_3	β-CH_3
**164**	β-*O*-Ac	α-OH	H	H	α-*O*-Ac	α-CH_3	H	H	H	H, $\Delta^{24,25}$	COOH	-	CH_3	β-CH_3
**165**	β-*O*-Ac	α-OH	H	H	α-*O*-Ac	α-CH_3	H	β-*O*-Ac	H	H, $\Delta^{24,25}$	COOH	-	CH_3	β-CH_3
**166**	β-OH	=O	α-OH	H	H	α-CH_3	H	H	H	H, $\Delta^{24,25}$	CH_2OH	-	CH_2OH	β-CH_3

Cpd	R1	R2	R3	R4	R5	R6	R7	R8	R9	R10	R11	R12	R13	R14
**167**	β-OH	=O	H	β-OH	=O	α-CH_3	H	H	H	H, $\Delta^{24,25}$	COOH	-	CH_3	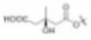
**168**	β-OH	=O	H	β-*O*-Ac	=O	α-CH_3	H	H	H	H, $\Delta^{24,25}$	COOH	-	CH_3	β-CH_3
**169**	=O	=O	α-OH	H	H	α-CH_3	H	H	H	H, $\Delta^{24,25}$	CHO	-	CH_3	β-CH_3
**170**	β-OH	H	H	H	H	α-COOH	H	H	H	H, $\Delta^{24,25}$	CH_2OH	-	CH_3	β-CH_3
**171**	α-*O*-Ac	H	H	H	H	CH_2O-β-D -xylosyl	H	H	H	H, $\Delta^{24,25}$	CH_3	-	CH_3	β-CH_3
**172**	α-*O*-Ac	H	H	H	H	α-COOH	H	H	H	H, $\Delta^{24,25}$	CH_3	-	CH_3	β-CH_3
**173**	=O	H	H	H	H	α-COO-β-D-glucopy ranosyl	H	H	H	H, $\Delta^{24,25}$	CH_3	-	CH_3	β-CH_3
**174**	α-*O*-Ac	H	H	H	H	α-COOH	H	H	H	H, $\Delta^{24,25}$	CH_3	-	CH_3	β-CH_3
**175**	=O	H	H	H	H	α-COOH	H	H	H	H, $\Delta^{24,25}$	CH_3	-	CH_3	β-CH_3
**176**	β-OH	H	H	H	H	α-COOH	H	H	H	H, $\Delta^{24,25}$	CH_3	-	CH_3	β-CH_3
**177**	=O	β-OH	=O	β-OH	=O	α-CH_3	H	H	H	H, $\Delta^{24,25}$	COOH	-	CH_3	β-CH_2OH
**178**	=O	β-OH	=O	β-OH	α-OH	α-CH_3	H	H	H	H, $\Delta^{24,25}$	COOH	-	CH_3	β-CH_2OH
**179**	=O	α-OH	=O	β-OH	α-OH	α-CH_3	H	H	H	H, $\Delta^{24,25}$	COOH	-	CH_3	β-CH_2OH
**180**	=O	=O	=O	β-OH	α-OH	α-CH_3	H	H	H	H, $\Delta^{24,25}$	COOH	-	CH_3	β-CH_2OH
**181**	=O	β-OH	=O	β-*O*-Ac	=O	α-CH_3	H	H	H	H, $\Delta^{24,25}$	COOH	-	CH_3	β-CH_2OH
**182**	=O	α-OH	=O	β-*O*-Ac	α-OH	α-CH_3	H	H	H	H, $\Delta^{24,25}$	COOH	-	CH_3	β-CH_2OH
**183**	=O	=O	=O	β-*O*-Ac	α-OH	α-CH_3	H	H	H	H, $\Delta^{24,25}$	COOH	-	CH_3	β-CH_2OH
**184**	=O	=O	=O	β-*O*-Ac	=O	α-CH_3	H	H	H	H, $\Delta^{24,25}$	COOH	-	CH_3	β-CH_2OH
**185**	=O	H	=O	β-*O*-Ac	α-OH	α-CH_3	H	H	H	H, $\Delta^{24,25}$	COOH	-	CH_3	β-CH_2OH
**186**	=O	β-OH	=O	β-*O*-Ac	=O	α-CH_3	H	H	H	H, $\Delta^{24,25}$	$COOCH_3$	-	CH_3	β-CH_2OH
**187**	=O	=O	=O	β-OH	=O	α-CH_3	H	H	H	H, $\Delta^{24,25}$	COOH	-	CH_3	β-CH_2OH
**188**	=O	H	=O	β-OH	α-OH	α-CH_3	H	H	H	H, $\Delta^{24,25}$	COOH	-	CH_3	β-CH_2OH
**189**	=O	β-OH	=O	H	α-OH	α-CH_3	H	H	H	H, $\Delta^{24,25}$	COOH	-	CH_3	β-CH_2OH
**190**	=O	=O	=O	H	=O	α-CH_3	H	H	H	H, $\Delta^{24,25}$	COOH	-	CH_3	β-CH_2OH
**191**	=O	H	=O	H	α-OH	α-CH_3	H	H	H	H, $\Delta^{24,25}$	COOH	-	CH_3	β-CH_2OH
**192**	=O	H	=O	β-OH	α-OH	α-CH_3	H	H	H	H, $\Delta^{24,25}$	COOH	-	CH_3	β-CH_2OH
**193**	=O	α-OH	H	H	α-OH	α-CH_3	H	H	H	H, $\Delta^{24,25}$	CH_2OH	-	CH_3	β-CH_3
**194**	=O	α-OH	H	H	α-OH	α-CH_3	H	H	H	H, $\Delta^{24,25}$	CH_2OH	-	CH_2OH	β-CH_3
**195**	=O	=O	H	H	H	α-CH_3	H	H	H	H, $\Delta^{24,25}$	COOH	-	CH_3	β-CH_3
**196**	=O	=O	=O	H	β-OH	α-CH_3	H	H	H	H, $\Delta^{24,25}$	COOH	-	CH_3	β-CH_3
**197**	=O	=O	α-OH	H	H	α-CH_3	H	H	H	H, $\Delta^{24,25}$	COOH	-	CH_3	β-CH_3
**198**	=O	=O	H	H	H	α-CH_2OH	H	H	H	H, $\Delta^{24,25}$	CH_2OH	-	CH_3	β-CH_3
**199**	=O	=O	=O	H	β-OH	CH_2Cl	ξ-OH	H	H	H, $\Delta^{24,25}$	COOH	-	CH_3	CH_2OH
**200**	=O	=O	=O	H	β-OH	CH_3	ξ-OH	H	H	H, $\Delta^{24,25}$	COOH	-	CH_3	β-CH_2OH
**201**	=O	=O	=O	H	β-OH	CH_3	ξ-OH	H	H	H, $\Delta^{24,25}$	COOH	-	CH_3	β-CH_3
**202**	β-OH	=O	=O	H	β-OH	CH_3	ξ-OH	H	H	H, $\Delta^{24,25}$	COOH	-	CH_3	β-CH_2OH
**203**	=O	=O	=O	H	=O	CH_3	ξ-OH	H	H	H, $\Delta^{24,25}$	COOH	-	CH_3	CH_3
**204**	β-OH	=O	H	H	H	β-CH_3	H	H	H	H, $\Delta^{24,25}$	CH_2OH	-	CH_2OH	β-CH_3
**205**	=O	β-CH_3	H	H	α-OH	β-CH_3	H	H	H	H, $\Delta^{24,25}$	CH_2OH	-	CH_2OH	β-CH_3
**206**	=O	=O	H	H	H	α-CH_3	H	H	H	H, $\Delta^{24,25}$	CH_2OH	-	CH_2OH	β-CH_3
**207**	β-*O*-Ac	H	H	H	α-*O*-Ac	α-CH_3	H	H	H	H, $\Delta^{24,25}$	COOH	-	CH_3	β-CH_3
**208**	α-*O*-Ac	=O	=O	H	α-*O*-Ac	α-CH_3	H	β-*O*-Ac	H	H, $\Delta^{24,25}$	COOH	-	CH_3	β-CH_3
**209**	α-*O*-Ac	=O	=O	H	α-OH	α-CH_3	H	β-*O*-Ac	H	H, $\Delta^{24,25}$	COOH	-	CH_3	β-CH_3
**210**	=O	=O	H	H	H	α-CH_3	H	β-*O*-Ac	H	H, $\Delta^{24,25}$	COOH	-	CH_3	β-CH_3
**211**	β-*O*-Ac	=O	H	H	H	α-CH_3	H	β-*O*-Ac	H	H, $\Delta^{24,25}$	COOH	-	CH_3	β-CH_3
**212**	β-OH	=O	H	H	H	α-COOH	H	=O	H	H, $\Delta^{24,25}$	CH_3	-	CH_3	β-CH_3
**213**	=O	α-*O*-Ac	H	H	H	α-OH	H	H	H	H, $\Delta^{24,25}$	COOH	-	CH_3	β-CH_3
**214**	β-OH	H	H	H	H	α-CH_3	H	OH	H	H, $\Delta^{24,25}$	CH_2OH	-	CH_3	β-CH_3
**215**	β-OH	H	H	H	H	α-CH_3	H	β-*O*-Ac	H	H, $\Delta^{24,25}$	COOH	-	CH_3	β-CH_3
**216**	α-OH	=O	=O	H	H	α-CH_3	H	β-*O*-Ac	H	H, $\Delta^{24,25}$	COOH	-	CH_3	β-CH_3
**217**	β-OH	=O	α-OH	H	H	α-CH_3	H	H	H	H, $\Delta^{24,25}$	CH_2OH	-	CH_3	β-CH_3
**218**	=O	α-OH	H	H	H	α-CH_3	H	H	H	H, $\Delta^{24,25}$	COOH	-	CH_3	β-CH_3
**219**	=O	α-*O*-CH_3	H	H	H	α-CH_3	H	H	H	H, $\Delta^{24,25}$	COOH	-	CH_3	β-CH_3
**220**	β-*O*-Ac	α-*O*-CH_3	H	H	H	α-CH_3	H	H	β-*O*-Ac	H, $\Delta^{24,25}$	COOH	-	CH_3	β-CH_3
**221**	β-*O*-Ac	α-*O*-CH_3	H	H	α-*O*-Ac	α-CH_3	H	H	β-*O*-Ac	H, $\Delta^{24,25}$	COOH	-	CH_3	β-CH_3
**222**	β-*O*-Ac	α-*O*-CH_3	H	H	α-*O*-Ac	α-CH_3	H	H	H	H, $\Delta^{24,25}$	COOH	-	CH_3	β-CH_3
**223**	=O	β-OH	H	H	H	α-CH_3	H	H	H	α-OH	H_2, $\Delta^{25,26}$	-	CH_3	β-CH_3
**224**	=O	=O	H	H	H	α-CH_3	H	H	H	α-OH	H_2, $\Delta^{25,26}$	-	CH_3	β-CH_3
**225**	=O	H	H	H	H	α-CH_3	H	H	H	CH_3	H_2, $\Delta^{25,26}$	-	CH_3	β-CH_3
**226**	α-*O*-Ac	H	H	H	H	α-COOH	H	H	H	CH_3	H_2, $\Delta^{25,26}$	-	CH_3	β-CH_3
**227**	α-*O*-Ac	H	H	H	H	α-COO-β D- xylosyl	H	H	H, $\Delta^{23,24}$	CH_3	H_2, $\Delta^{25,26}$	-	CH_3	β-CH_3
**228**	α-*O*-Ac	=O	H	H	H	α-COOH	H	H	H	=CH_2	CH_3	H	CH_3	β-CH_3
**229**	=O	=O	=O	H	=O	CH_3, $\Delta^{17,20}$	-	H	=O	H	COOH	H	CH_3	β-CH_3
**230**	β-OH	β-OH	=O	H	β-OH	CH_3, $\Delta^{17,20}$	-	H	=O	H	CH_3	H	$COOCH_3$	β-CH_3
**231**	=O	=O	=O	β-*O*-Ac	=O	CH_3, $\Delta^{17,20}$	-	H	=O	H	CH_3	H	COOH	β-CH_3

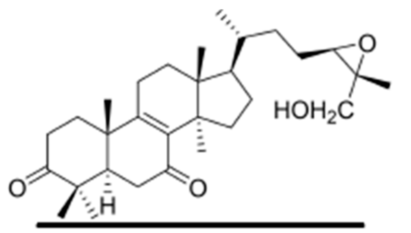

Cpd	R1	R2
**235**	*α*-OH	*β*-CH_3
**236**	=O	*α*-CH_3

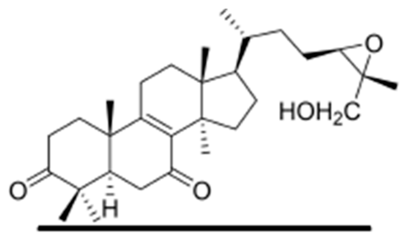

Cpd	R1	R2
**235**	*α*-OH	*β*-CH_3
**236**	=O	*α*-CH_3

Figure S15 Structures of compounds 232-234 Figure S16 Structures of compounds
235-236

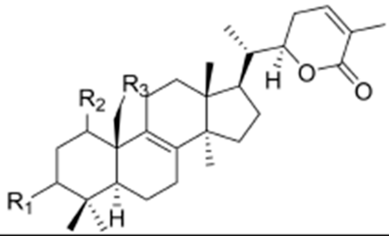

Cpd	R1	R2	R3
**237**	*β*-OH	H	OAc
**238**	=O	H	H
**239**	*β*-OH	H	CH_3
**240**	*β*-OH	*β*-OH	CH_3

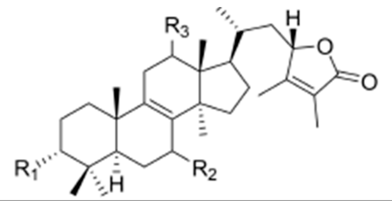

Cpd	R1	R2	R3
**241**	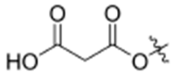	H	*β*-OH
**242**		=O	*β*-OH
**243**	H	H	*β*-OH
**244**	OH	H	H

Figure S16 Structures of compounds 237-240 Figure S17 Structures of compounds
241-244

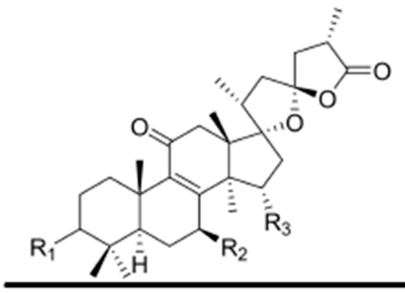

Cpd	R1	R2	R3
**245**	=O	H	H
**246**	=O	OH	OH
**247**	=O	H	OAc
**248**	=O	OAc	OH
**249**	*β*-OH	*β*-OH	*α*-OH

Figure S18 Structures of compounds 245-249

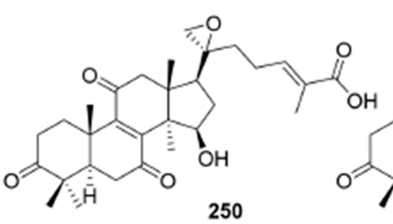
250
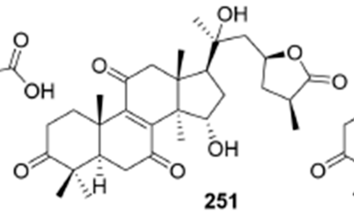
251
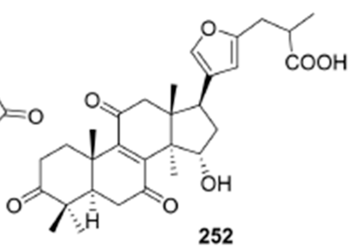
252

Figure S19 Structures of compounds 250-252

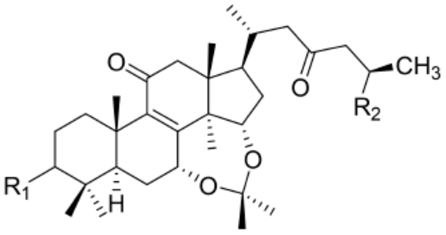

Cpd	R1	R2
**253**	*β*-OH	COOH
**254**	=O	$COOCH_3$

Figure S20 Structures of compounds 253-254

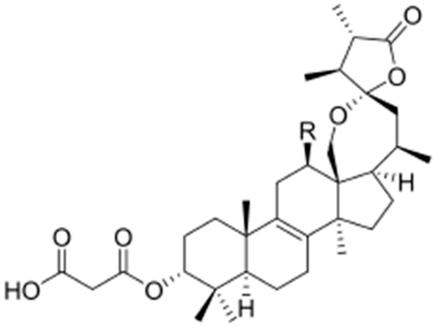

Cpd	R
**255**	OH
**256**	H

Figure S21 Structures of compounds 255-256

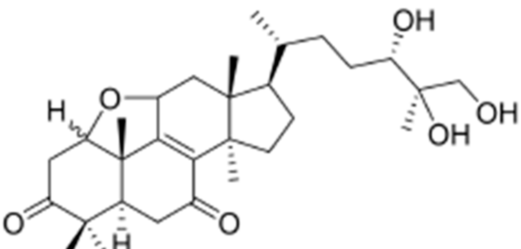

257

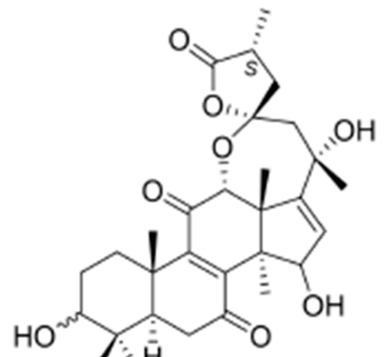

258

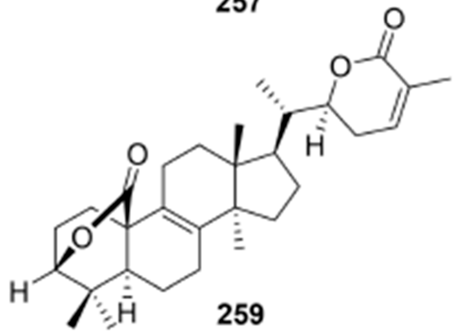

259

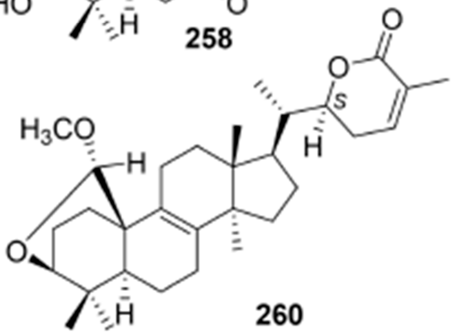

260

Figure S22 Structures of compounds 257-260

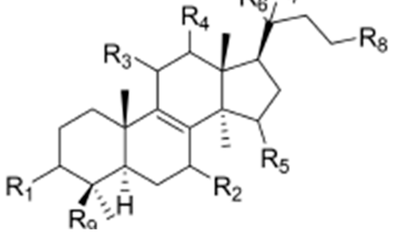

Figure S23 Chemical structure of compound 261-301

Table S15 Chemical Structures of compounds 261-301

Cpd	R1	R2	R3	R4	R5	R6	R7	R8	R9
**261**	β-OH	β-OH	=O	H	=O	α-CH_3	H	COOBu	CH_3
**262**	=O	β-OH	=O	H	=O	α-CH_3	H	COOBu	CH_3
**263**	β-OH	β-OH	=O	H	=O	=CH_2	$\Delta^{20,21}$	COOH	CH_3
**264**	=O	β-OH	=O	H	=O	β-CH_3	ξ-OH	COOH	CH_3
**265**	=O	=O	=O	β-*O*-Ac	=O	β-CH_3	H	$COOCH_3$	CH_3
**266**	=O	β-OH	=O	H	=O	=CH_2	$\Delta^{20,21}$	COOH	CH_3
**267**	=O	β-OH	=O	H	=O	=CH_2	$\Delta^{20,21}$	$COOCH_3$	CH_3
**268**	β-OH	β-OH	=O	H	=O	α-CH_3	H	COOH	CH_3
**269**	=O	=O	=O	β-*O*-Ac	=O	β-CH_3	H	COOH	CH_3
**270**	β-OH	=O	=O	β-*O*-Ac	=O	β-CH_3	H	$COOCH_3$	CH_3
**271**	=O	=O	=O	H	=O	β-CH_3	H	$COOCH_3$	CH_3
**272**	=O	OH	=O	H	=O	α-CH_3	H	COOEt	CH_3
**273**	β-O-CHO	β-OH	=O	OH	=O	β-CH_3	H	COOH	CH_3
**274**	=O	β-OH	=O	H	=O	β-CH_3	H	COOH	CH_3
**275**	=O	β-OH	=O	β-OH	=O	β-CH_3	H	COOH	CH_3
**276**	β-OH	β-OH	=O	β-OH	=O	β-CH_3	H	COOH	CH_3
**277**	=O	=O	H	H	H	α-CH_3	H	COOH	CH_3
**278**	β-OH	β-OH	=O	β-*O*-Ac	=O	α-CH_3	H	COOH	CH_3
**279**	β-OH	β-OH	=O	β-*O*-Ac	=O	α-CH_3	H	$COOCH_3$	CH_3
**280**	=O	β-OH	=O	H	α-OH	α-CH_3	H	$COOCH_3$	CH_3
**281**	β-OH	=O	=O	H	=O	α-CH_3	H	COOH	CH_3
**282**	=O	=O	=O	β-*O*-Ac	=O	β-CH_3	H	$COOCH_3$	CH_3
**283**	β-OH	=O	=O	β-*O*-Ac	=O	β-CH_3	H	$COOCH_3$	CH_3
**284**	β-OH	β-OH	=O	H	=O	α-CH_3	H	$COOCH_3$	CH_3
**285**	=O	β-OH	=O	β-OH	=O	α-CH_3	H	COOBu	CH_3
**286**	=O	β-OH	=O	H	=O	α-CH_3	H	$COOCH_3$	CH_3
**287**	=O	=O	=O	β-*O*-Ac	=O	α-CH_3	H	COOH	CH_3
**288**	=O	=O	=O	β-*O*-Ac	=O	β-CH_3	ξ-OH	COOH	CH_3
**289**	=O	=O	=O	H	=O	β-CH_3	ξ-OH	COOH	CH_3
**290**	β-OH	=O	=O	β-*O*-Ac	=O	β-CH_3	ξ-OH	COOH	CH_3
**291**	β-OH	β-OH	=O	H	=O	β-CH_3	ξ-OH	COOH	CH_3
**292**	β-OH	β-OH	=O	β-*O*-Ac	=O	β-CH_3	ξ-OH	COOH	CH_3
**293**	=O	=O	=O	H	=O	α-CH_3	H	COOH	CH_3
**294**	β-OH	β-OH	=O	H	=O	α-CH_3	H	$COOCH_3$	CH_3
**295**	β-OH	=O	=O	β-*O*-Ac	=O	α-CH_3	H	COOH	CH_3
**296**	=O	β-OH	=O	H	=O	α-CH_3	H	COOH	CH_3
**297**	β-OH	β-OH	=O	H	=O	β-CH_3	H	CH_3	CH_2OH
**298**	β-OH	=O	=O	H	=O	β-CH_3	H	CH_3	CH_2OH
**299**	β-OH	=O	=O	H	=O	β-CH_3	H	CH_3	CH_2OH
**300**	β-OH	β-OH	=O	H	=O	α-CH_3	H	CH_3	CH_2OH
**301**	=O	=O	=O	H	=O	=CH_2	$\Delta^{20,21}$	$COOCH_3$	CH_3

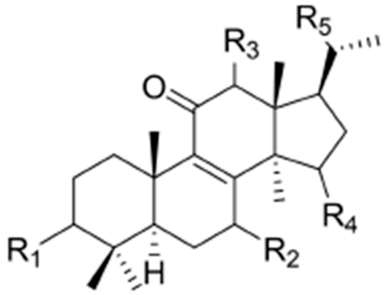

Cpd	R1	R2	R3	R4	R5
**302**	OH	OH	H	=O	=O
**303**	OH	OH	H	=O	OH
**304**	*β*-OH	*β*-OH	H	*α*-OH	CH_2OH
**305**	OH	*β*-OH	$COOCH_3$	=O	=O
**306**	OH	=O	$COOCH_3$	=O	=O
**307**	OAc	=O	H	*α*-OH	=O

Figure S24 Structures of compounds 302-307

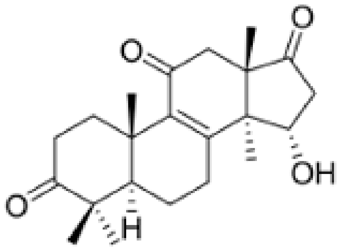

308

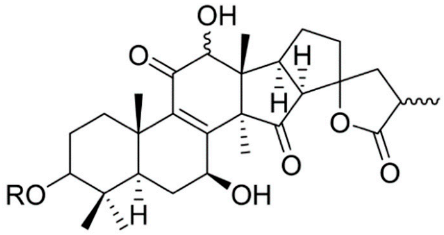

Cpd	R
**309**	=O
**310**	*β*-OH

Figure S25 Structures of compounds 309-310

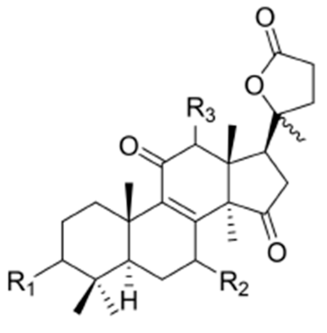

Cpd	R1	R2	R3
**311**	=O	*β*-OH	*α*-OH
**312**	=O	*β*-OH	H
**313**	=O	=O	H
**314**	=O	=O	*β*-*O*-Ac
**315**	*β*-OH	*β*-OH	*β*-OH
**316**	*β*-OH	*β*-OH	=O
**317**	*β*-OH	*β*-OH	H

Figure S26 Structures of compounds 311-317

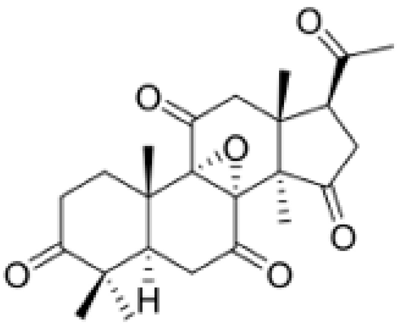

318

Figure S27 Structures of compound 318

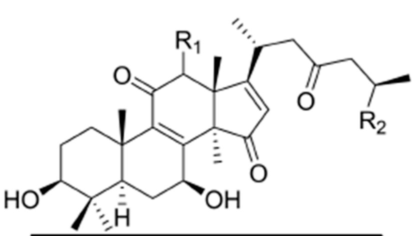

Cpd	R1	R2
**319**	H	COOH
**320**	H	$COOCH_3$
**321**	β-*O*-Ac	COOH

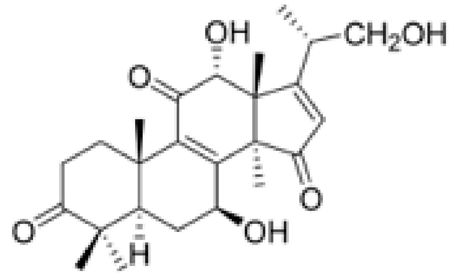

322

Figure S28 Structures of compounds 319-322

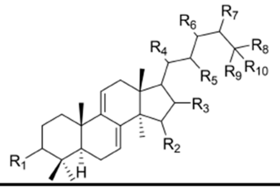

Cpd	R1	R2	R3	R4	R5	R6	R7	R8	R9	R10
**323**	=O	H	H	β-CH_3	H	H	H, $\Delta^{24,25}$	CHO	CH_3	-
**324**	=O	H	H	β-CH_3	H	H	H, $\Delta^{24,25}$	CH_2OH	CH_3	-
**325**	=O	α-OH	H	β-CH_3	H	H	H, $\Delta^{24,25}$	COOH	CH_3	-
**326**	α-OH	H	α-OH	α-COOH	H	H	H, $\Delta^{24,25}$	CH_3	CH_3	-
**327**	α-OH	H	α-OH	α-COOH	H	H	H, $\Delta^{24,25}$	CH_2OH	CH_3	-
**328**	=O	H	H	α-CH_3	H	H	H, $\Delta^{24,25}$	COOH	CH_3	-
**329**	=O	H	H	β-CH_3	H	H	H, $\Delta^{24,25}$	COOH	CH_3	-
**330**	=O	α-OH	H	α-CH_3	H	H	H, $\Delta^{24,25}$	CH_2OH	CH_3	-
**331**	α-*O*-Ac	α-*O*-Ac	H	β-CH_3	H	H	H, $\Delta^{24,25}$	COOH	CH_3	-
**332**	β-*O*-Ac	α-OH	H	β-CH_3	H	H	H, $\Delta^{24,25}$	COOH	CH_3	-
**333**	=O	H	H	α-CH_3	H	H	H, $\Delta^{24,25}$	CH_2OH	CH_3	-
**334**	β-OH	H	H	α-CH_3	H	H	H, $\Delta^{24,25}$	CH_2OH	CH_3	-
**335**	β-OH	H	H	α-CH_3	H	H	H, $\Delta^{24,25}$	CH_2OH	CH_2OH	-
**336**	β-*O*-Ac	α-*O*-Ac	H	β-CH_3	H	H	H, $\Delta^{24,25}$	COOH	CH_3	-
**337**	=O	H	=O	β-CH_3	H	H	H, $\Delta^{24,25}$	CH_2OH	CH_2OH	-
**338**	β-OH	H	H	β-CH_3	H	H	H, $\Delta^{24,25}$	CH_2OH	CH_3	-
**339**	α-*O*-Ac	α-OH	H	β-CH_3	β-*O*-Ac	H	H, $\Delta^{24,25}$	COOH	CH_3	-
**340**	=O	α-OH	H	α-CH_3	H	H	H, $\Delta^{24,25}$	CH_2OH	CH_2OH	-
**341**	α-*O*-Ac	α-*O*-Ac	H	α-CH_3	β-*O*-Ac	H	H, $\Delta^{24,25}$	COOH	CH_3	-

Cpd	R1	R2	R3	R4	R5	R6	R7	R8	R9	R10
**342**	α-OH	H	H	α-CH_3	β-*O*-Ac	H	H, $\Delta^{24,25}$	COOH	CH_3	-
**343**	α-*O*-Ac	H	H	α-CH_3	β-*O*-Ac	H	H, $\Delta^{24,25}$	COOH	CH_3	-
**344**	=O	H	H	α-CH_3	β-*O*-Ac	H	H, $\Delta^{24,25}$	COOH	CH_3	-
**345**	β-*O*-Ac	α-*O*-Ac	H	β-CH_3	β-*O*-Ac	H	H, $\Delta^{24,25}$	COOH	CH_3	-
**346**	α-OH	α-*O*-Ac	H	β-CH_3	H	=O	H, $\Delta^{24,25}$	COOH	CH_3	-
**347**	α-*O*-Ac	α-*O*-Ac	H	β-CH_3	H	=O	H, $\Delta^{24,25}$	COOH	CH_3	-
**348**	α-*O*-Ac	α-OH	H	β-CH_3	H	=O	H, $\Delta^{24,25}$	COOH	CH_3	-
**349**	α-*O*-Ac	α-OH	H	β-CH_3	H	H	H, $\Delta^{24,25}$	COOH	CH_3	-
**350**	β-OH	α-*O*-Ac	H	β-CH_3	H	H	H, $\Delta^{24,25}$	COOH	CH_3	-
**351**	β-*O*-Ac	α-OH	H	β-CH_3	H	H	H, $\Delta^{24,25}$	COOH	CH_3	-
**352**	=O	α-*O*-Ac	H	β-CH_3	H	H	H, $\Delta^{24,25}$	COOH	CH_3	-
**353**	=O	α-*O*-Ac	H	α-CH_3	β-*O*-Ac	H	H, $\Delta^{24,25}$	COOH	CH_3	-
**354**	α-OH	α-*O*-Ac	H	α-CH_3	β-*O*-Ac	H	H, $\Delta^{24,25}$	COOH	CH_3	-
**355**	α-*O*-Ac	α-OH	H	α-CH_3	β-*O*-Ac	H	H, $\Delta^{24,25}$	COOH	CH_3	-
**356**	=O	α-OH	H	α-CH_3	H	OH	H, $\Delta^{24,25}$	COOH	CH_3	-
**357**	β-OH	α-OH	H	α-CH_3	H	H	H, $\Delta^{24,25}$	CH_2OH	CH_2OH	-
**358**	β-OH	H	H	α-CH_3	H	H	H, $\Delta^{24,25}$	CHO	CH_3	-
**359**	=O	H	H	β-CH_3	H	H	H, $\Delta^{24,25}$	CH_2OH	CH_2OH	-
**360**	=O	H	H	α-CH_3	=O	H	H, $\Delta^{24,25}$	CH_2OH	CH_2OH	-
**361**	=O	H	H	α-CH_3	=O	H	H, $\Delta^{24,25}$	CH_2OH	CH_3	-
**362**	α-*O*-Ac	α-OH	H	β-CH_3	β-*O*-Ac	H	H, $\Delta^{24,25}$	COOH	CH_3	-
**363**	β-OH	α-*O*-Ac	H	β-CH_3	β-*O*-Ac	H	H, $\Delta^{24,25}$	COOH	CH_3	-
**364**	β-*O*-Ac	α-*O*-Ac	H	α-CH_3	β-*O*-Ac	H	H, $\Delta^{24,25}$	COOH	CH_3	-
**365**	=O	H	α-OH	α-COOH	H	H	H, $\Delta^{24,25}$	CH_3	CH_3	-
**366**	=O	α-OH	H	α-CH_3(β-OH)	H	H	H, $\Delta^{24,25}$	COOH	CH_3	-
**367**	β-OH	H	H	α-OH(β-CH_3)	H	H	H, $\Delta^{24,25}$	COOH	CH_3	-
**368**	α-OH	α-OH	H	CH_3	α-OH	H	H, $\Delta^{24,25}$	COOH	CH_3	-
**369**	β-OH	α-OH	H	α-CH_3(β-OH)	β-OH	H	H, $\Delta^{24,25}$	COOH	CH_3	-
**370**	α-*O*-Ac	α-*O*-Ac	H	CH_3	α-OH	H	H, $\Delta^{24,25}$	COOH	CH_3	-
**371**	β-*O*-Ac	α-*O*-Ac	H	CH_3	α-OH	H	H, $\Delta^{24,25}$	COOH	CH_3	-
**372**	α-OH	α-OH	H	CH_3	β-*O*-Ac	H	H, $\Delta^{24,25}$	COOH	CH_3	-
**373**	β-OH	α-OH	H	CH_3	β-*O*-Ac	H	H, $\Delta^{24,25}$	COOH	CH_3	-
**374**	=O	α-OH	H	α-CH_3	H	H	H, $\Delta^{24,25}$	COOH	CH_3	-
**375**	α-OH	α-OH	H	β-CH_3	H	H	H, $\Delta^{24,25}$	COOH	CH_3	-
**376**	β-OH	α-OH	H	β-CH_3	H	H	H, $\Delta^{24,25}$	COOH	CH_3	-
**377**	α-*O*-Ac	α-*O*-Ac	H	β-CH_3	H	H	H, $\Delta^{24,25}$	COOH	CH_3	-
**378**	=O	H	H	α-CH_3	β-OH	H	H, $\Delta^{24,25}$	COOH	CH_3	-
**379**	=O	α-OH	H	α-CH_3	H	H	H, $\Delta^{24,25}$	CH_2OH	CH_3	-
**380**	β-OH	H	H	α-CH_3	H	H	H, $\Delta^{24,25}$	COOH	CH_3	-
**381**	β-*O*-Ac	H	H	α-CH_3	H	H	H, $\Delta^{24,25}$	COOH	CH_3	-
**382**	β-*O*-Ac	H	H	α-CH_3	β-*O*-Ac	H	H, $\Delta^{24,25}$	COOH	CH_3	-
**383**	β-*O*-Ac	H	H	α-CH_3	β-*O*-Ac	H	H, $\Delta^{24,25}$	COOH	CH_3	-
**384**	=O	α-*O*-Ac	H	α-CH_3	H	H	α-OH	β-OH	CH_3	CH_2OH
**385**	=O	H	H	α-CH_3	H	H	α-OH	β-*O*-CH_3	CH_3	CH_2OH
**386**	β-OH	α-OH	H	α-CH_3	H, $\Delta^{22,23}$	H, $\Delta^{22,23}$	H, $\Delta^{24,25}$	COOH	CH_3	-
**387**	β-OH	α-OH	H	α-CH_3	H, $\Delta^{20,22}$	=O	H	COOH	CH_3	-
**388**	=O	H	H	α-CH_3	H	H	α-OH	CH_3	CH_2OH	OH
**389**	β-OH	H	H	α-CH_3	H	H	α-OH	CH_3	CH_2OH	β-*O*-CH_3
**390**	β-*O*-Ac	H	H	α-CH_3	H	H	OAc	CH_3	CH_2-*O*-Ac	OH
**391**	β-OH	H	H	α-CH_3	H	H	α-OH	CH_3	CH_3	OH
**392**	=O	H	H	β-CH_3	H	H	α-OH	CH_3	CH_2OH	H
**393**	β-OH	H	H	α-CH_3	H	H	OH	CH_3	CH_2OH	OH
**394**	=O	H	H	α-CH_3	H	H	OH	CH_3	CH_3	OH
**395**	β-OH	α-*O*-Ac	H	β-CH_3	H	H	H	CH_3	COOH	H

Figure S29 Structures of compound 261-301

Table S16 Structures of compounds 261-301

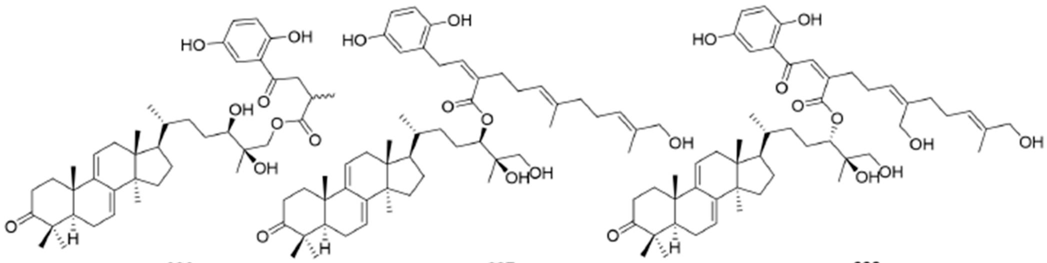

396 397 398

Figure S30 Structures of compounds 396-398

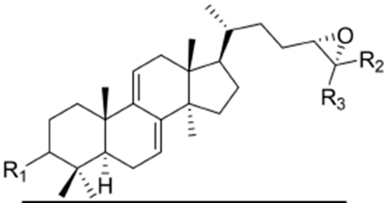

Cpd	R1	R2	R3
**399**	*β*-OH	CH_3	CH_2OH
**400**	=O	CH_3	CH_2OH
**401**	=O	CH_2OH	CH_2OH

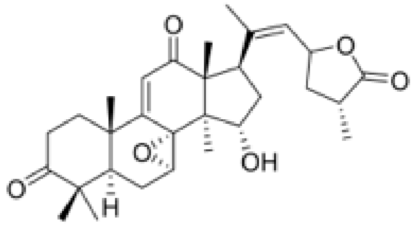

402

Figure S31 Structures of compounds 399-402

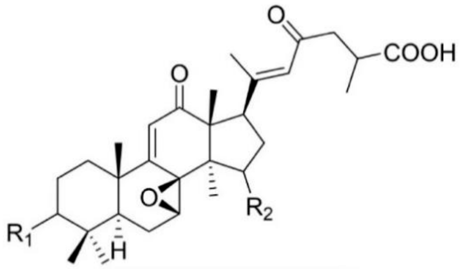

Cpd	R1	R2
**403**	=O	*α*-OH
**404**	*β*-OH	=O
**405**	=O	=O

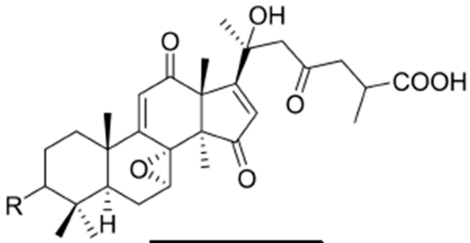

Cpd	R
**406**	=O
**407**	*β*-OH

Figure S32 Structures of compounds 403-407

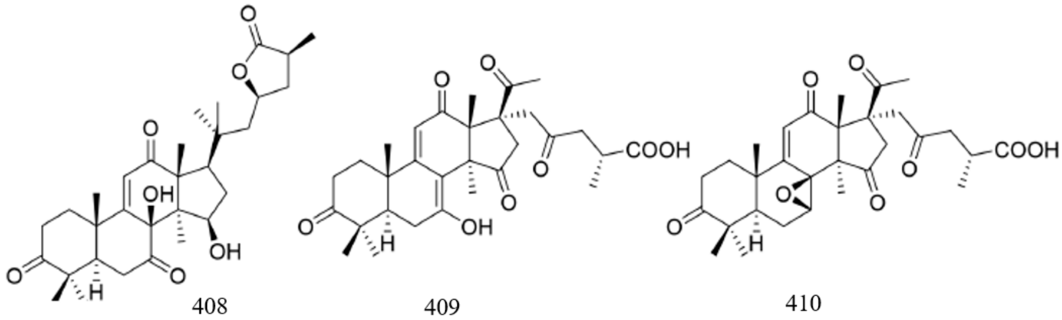

408 409 410

Figure S33 Structures of compounds 408-410

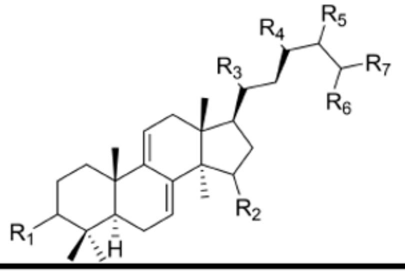

Cpd	R1	R2	R3	R4	R5	R6	R7
**411**	β-OH	H	α-CH_3	H	H, $\Delta^{24,25}$	H, $\Delta^{24,25}$	CHO
**412**	=O	H	β-CH_3	H	H, $\Delta^{24,25}$	H, $\Delta^{24,25}$	CHO
**413**	=O	H	β-CH_3	H	H, $\Delta^{24,25}$	H, $\Delta^{24,25}$	CH_2OH
**414**	=O	α-OH	α-CH_3	H	H, $\Delta^{24,25}$	H, $\Delta^{24,25}$	COOH
**415**	α-OH	α-OH	β-CH_3	H	H, $\Delta^{24,25}$	H, $\Delta^{24,25}$	COOH
**416**	β-OH	α-OH	β-CH_3	H	H, $\Delta^{24,25}$	H, $\Delta^{24,25}$	COOH
**417**	α-*O*-Ac	α-*O*-Ac	β-CH_3	H	H, $\Delta^{24,25}$	H, $\Delta^{24,25}$	COOH
**418**	=O	α-OH	α-CH_3	H	H, $\Delta^{24,25}$	H, $\Delta^{24,25}$	COOH
**419**	=O	α-OH	α-CH_3	H	H, $\Delta^{24,25}$	H, $\Delta^{24,25}$	CH_2OH
**420**	β-OH	H	α-CH_3	H	H, $\Delta^{24,25}$	H, $\Delta^{24,25}$	COOH
**421**	=O	H	α-CH_3	H	H, $\Delta^{24,25}$	CH_2OH	CH_3
**422**	β-OH	H	α-CH_3	H, $\Delta^{23,24}$	H, $\Delta^{24,25}$	=O	CH_3

Figure S34 Structures of compounds 411-422

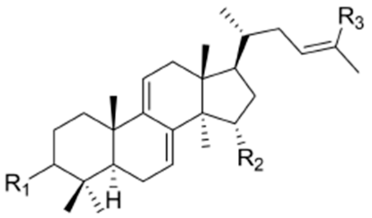

Cpd	R1	R2	R3
**423**	α-OH	H	COOH
**424**	=O	H	CH_2OH
**425**	β-OH	H	COOH

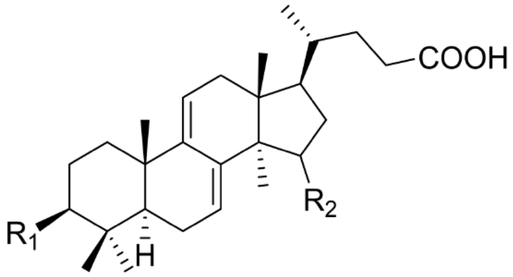

Cpd	R1	R2
**426**	=O	H
**427**	=O	α-OH

Figure S35 Structures of compounds423-427

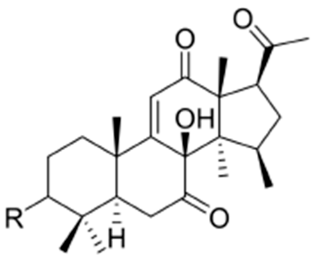

Cpd	R
**428**	β-OH
**429**	=O

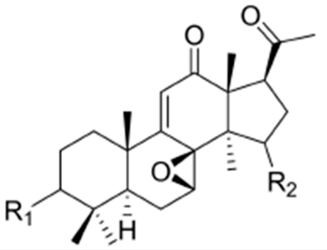

Cpd	R1	R2
**430**	=O	=O
**431**	β-OH	β-OH

Figure S36 Structures of compounds 428-431

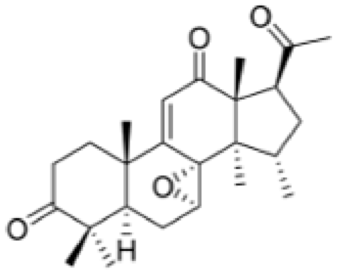

432

Figure S37 Structures of compound 432

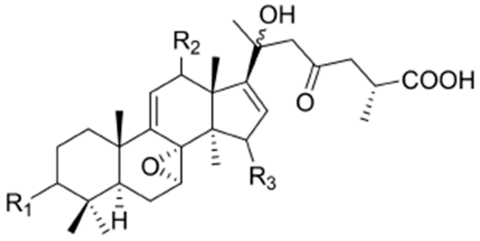

Cpd	R1	R2	R3
**434**	=O	=O	*β*-OH
**435**	*β*-OH	*α*-OH	=O

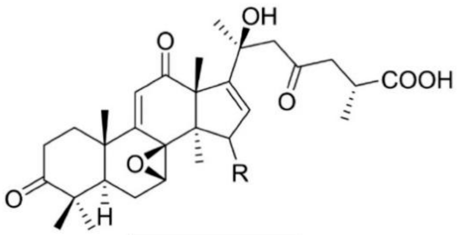

Cpd	R
**436**	OH
**437**	=O

Figure S38 Structures of compounds 434-437

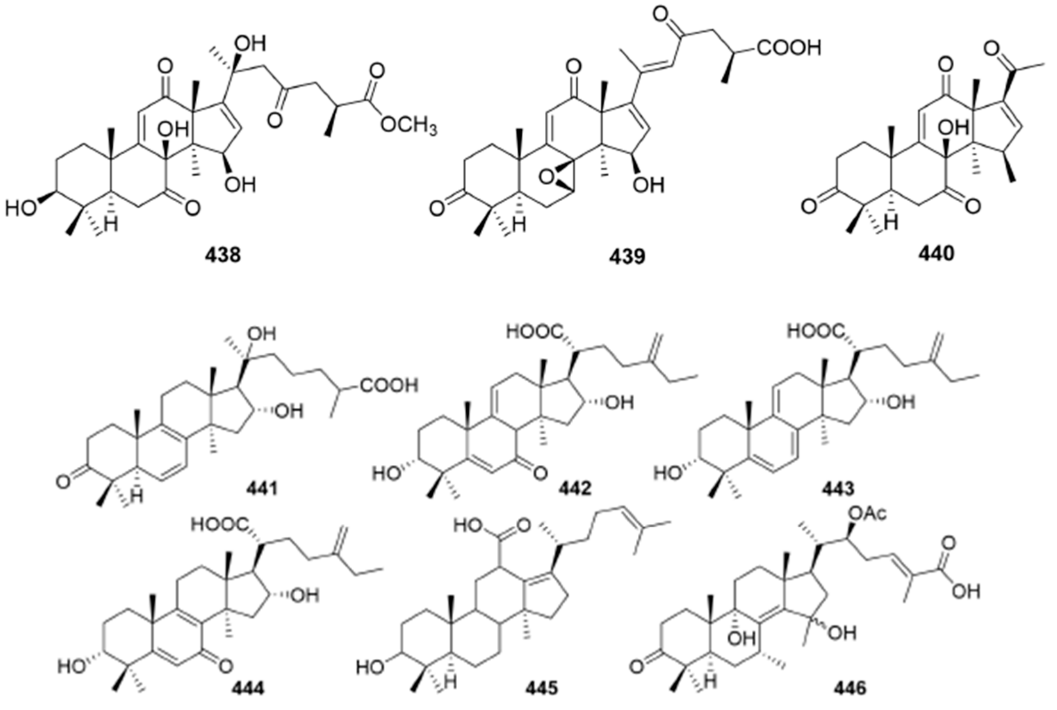

Figure S39 Structures of compounds 438-446

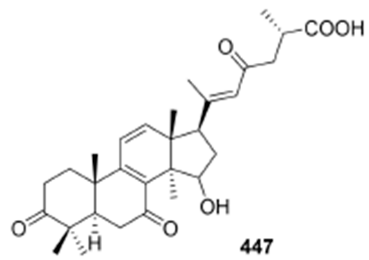

447

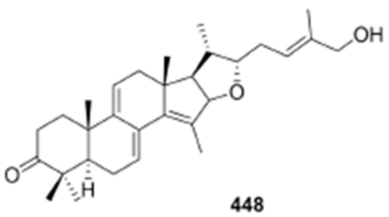

448

Figure S40 Structures of compounds 447-448

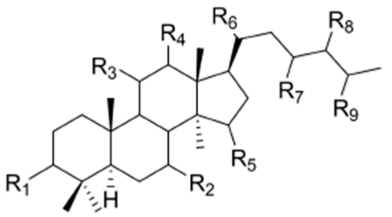

Cpd	R1	R2	R3	R4	R5	R6	R7	R8	R9
**449**	=O	=O	=O	H	*α*-OH	*β*-CH_3	=O	H	COOH
**450**	=O	=O	=O	H	*α*-OH	*β*-CH_3	=O	H	$COOCH_3$
**451**	=O	=O	=O	=O	=O	*β*-CH_3	=O	H	COOH
**452**	H	H	H	H	H	*α*-CH_3	H	ξ-CH_3	=O
**453**	=O	=O	=O	H	*β*-OH	CH_3, $\Delta^{20,22}$	=O	CH_3	$COOCH_3$

Figure S41 Structures of compounds 449-453

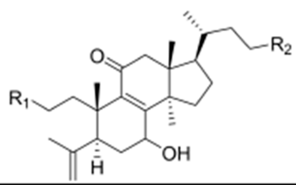

Cpd	R1	R2
**454**	$COOCH_3$	COOH
**455**	COOH	$COOCH_3$
**456**	$COOCH_3$	$COOCH_3$
**457**	COOH	COOH
**458**	$COOCH_2CH_3$	COOH

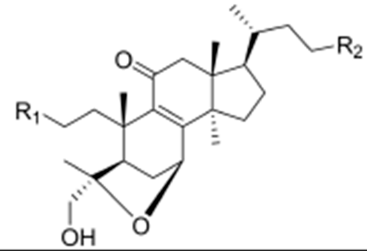

Cpd	R1	R2
**459**	COOH	COOH
**460**	$COOCH_3$	$COOCH_3$

Figure S42 Structures of compounds 454-460

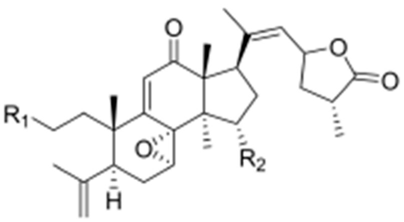

Cpd	R1	R2
**461**	COOH	*α*-*O*-Ac
**462**	COOH	*α*-OH
**463**	COOH	*β*-OH
**464**	$COOCH_3$	*α*-OH

Figure S43 Structures of compounds 461-464

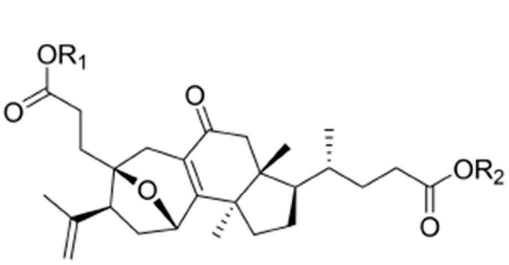

Cpd	R1	R2
**465**	H	CH_3
**466**	CH_3	H

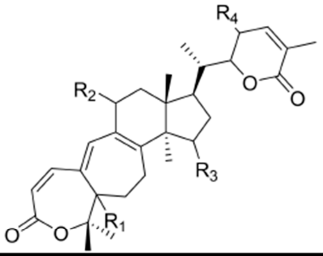

Cpd	R1	R2	R3	R4
**467**	*α*-H	*β*-H	*β*-OH	H
**468**	*α*-H	*β*-H	*β*-*O*-Ac	H
**469**	*α*-H	*β*-OH	*β*-*O*-Ac	H
**470**	ξ-OH	H	*β*-*O*-Ac	H
**471**	H	H	*β*-*O*-Ac	OH

Figure S44 Structures of compounds 465-471

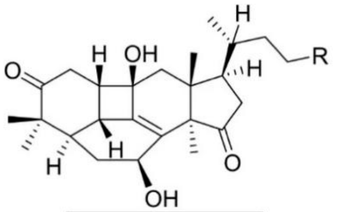

Cpd	R
**472**	COOH
**473**	$COOCH_3$

Figure S45 Structures of compounds 472-473

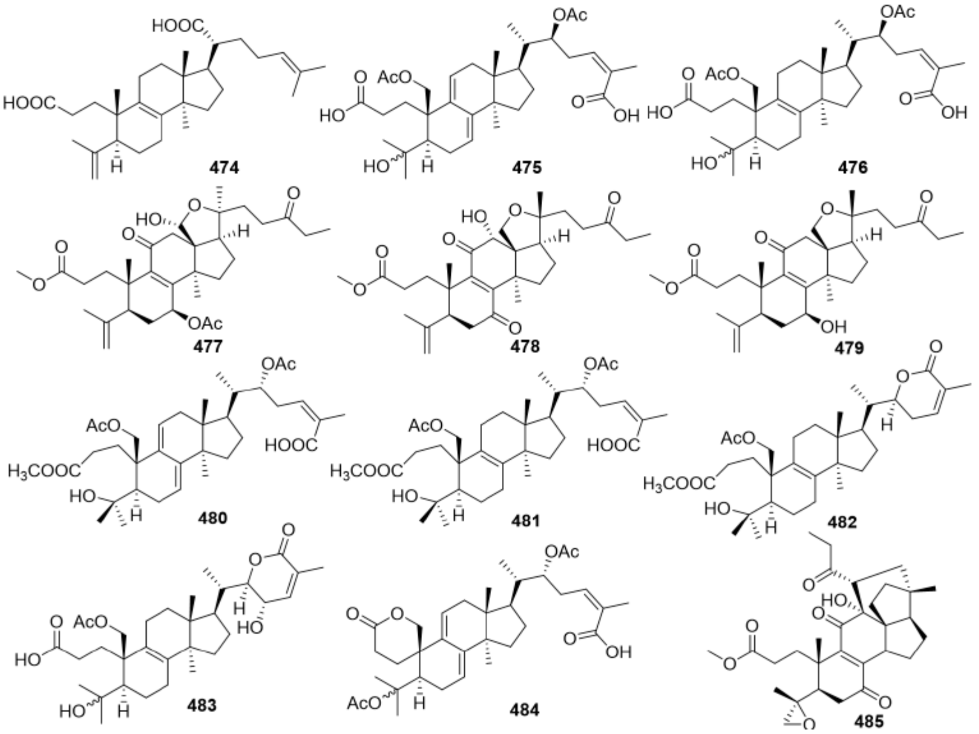

Figure S46 Structures of compounds 474-485

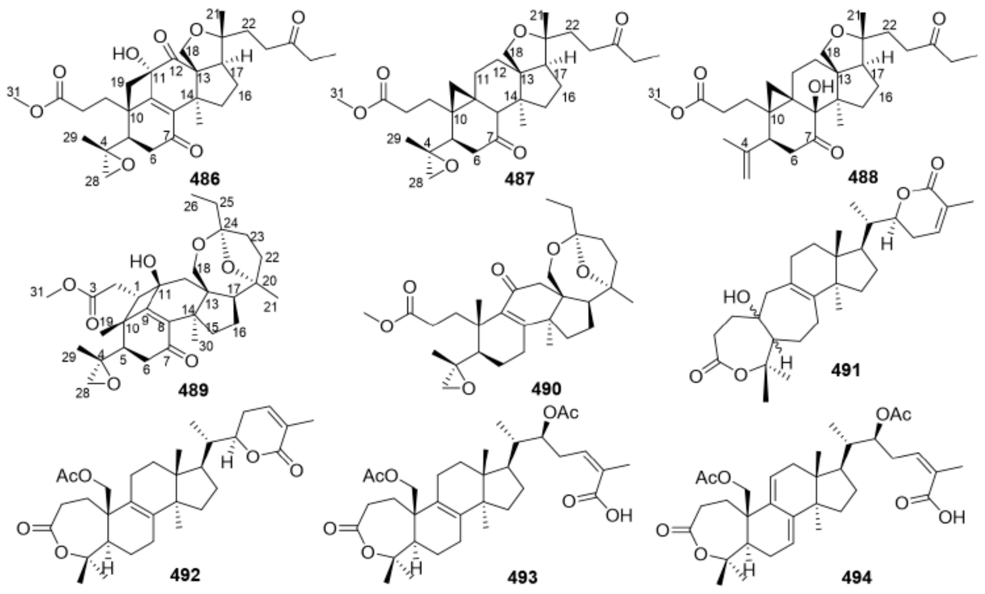

Figure S47 Structures of compounds 486-494

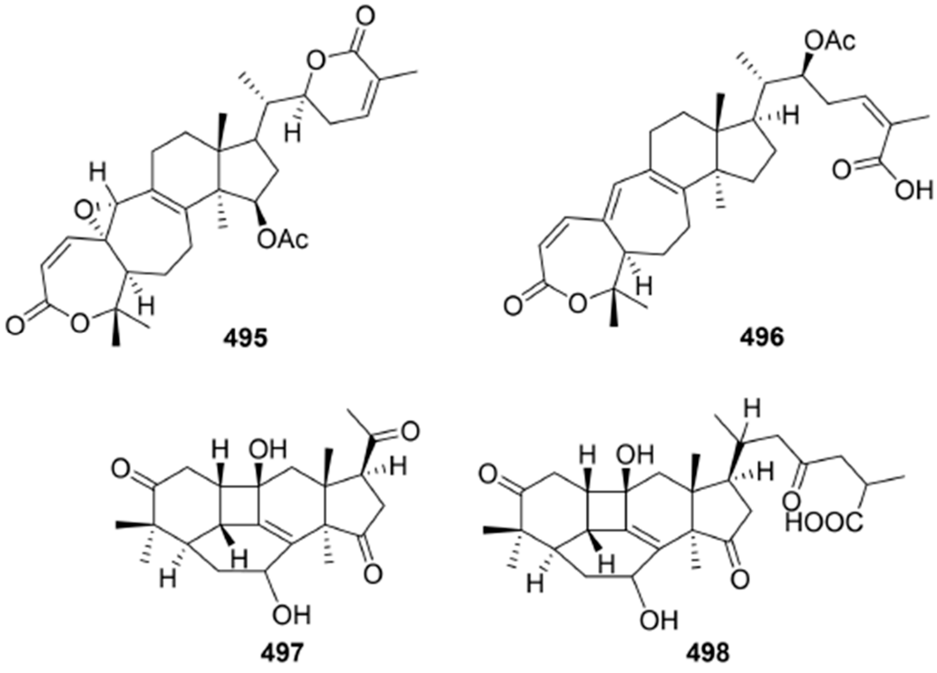

Figure S48 Structures of compounds 495-498

Table S17 Chemical Structures of compounds 1-21

cpd	name	structure
1	Isolarch alcohol	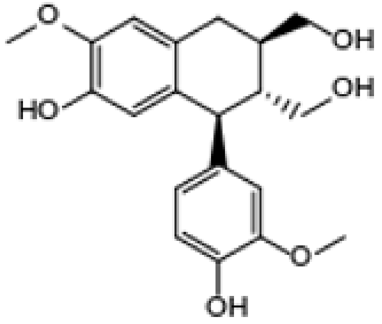
2	Dihydrodehydrodic Onifery alcohol	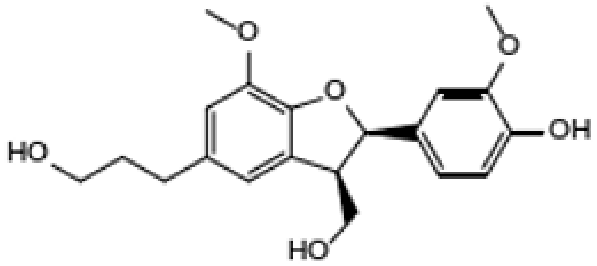
3	Bitter neool	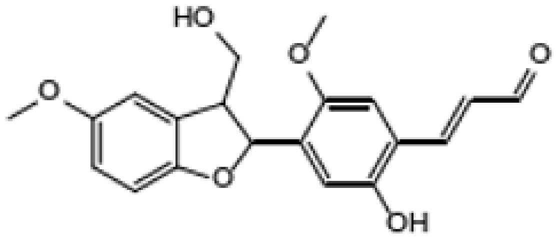
4	Vanillil	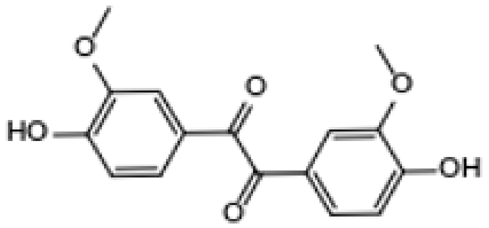
5	2,3-Dichloroquinoxaline	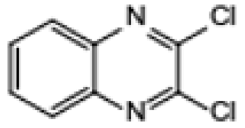

cpd	name	structure
6	1,5-Dicaffeoylquinine sour	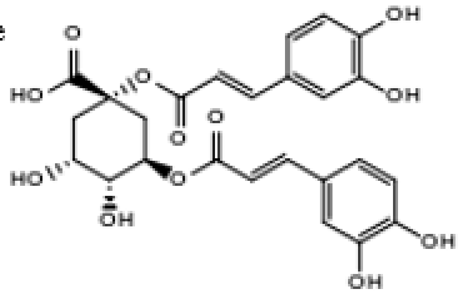
7	Protocatechin	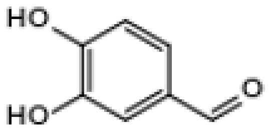
8	3-carboxy-6,7-dihydroxy-1-(3',4'-dihydroxyphenyl)-naphthalene	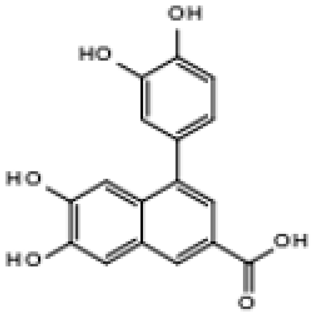
9	Ethyl caffeate	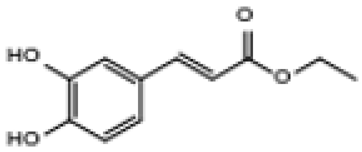
10	5,6,7-Trimethoxyincense Legumin	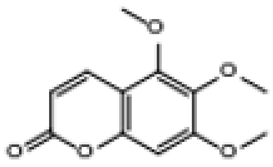
11	Emodin-1-O-grapes glycoside	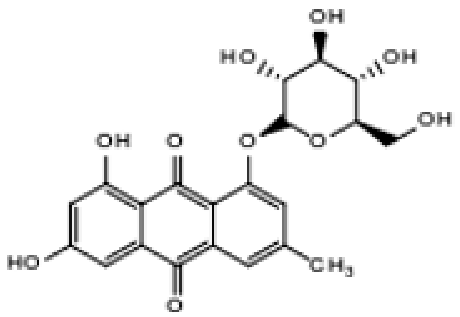

cpd	name	structure
12	p-Coumaric acid	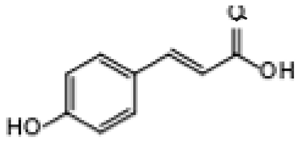
13*	Dipterin	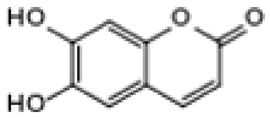
14*	methyl 3,5-dihydroxy-4-methoxybenzoate	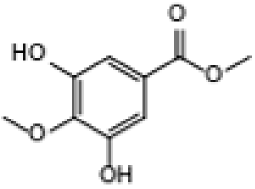
15	Isazinepidine	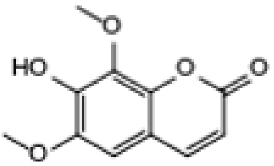
16	(-)-Syringol resin	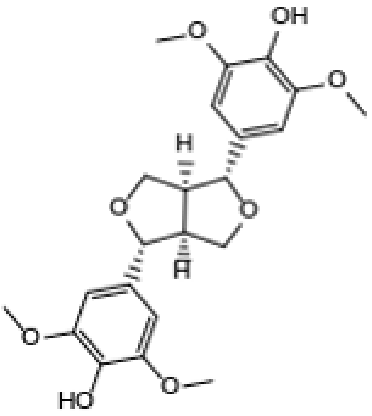
17	(+)-Syringin	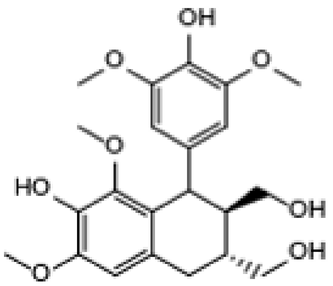

cpd	name	structure
18	Trans-caffeic acid	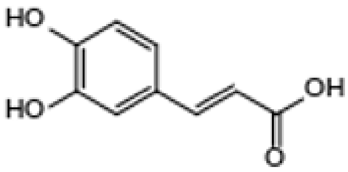
19	Emodin	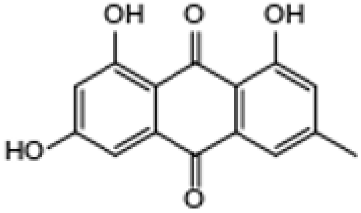
20	vanillic acid	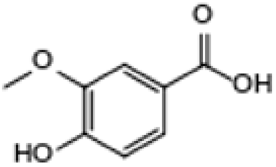
21	Methyl caffeate	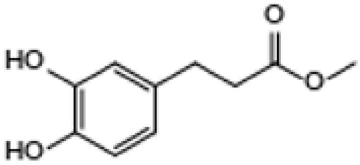

Albizia julibrissin durazz bark

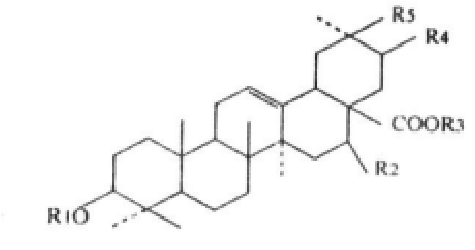

I

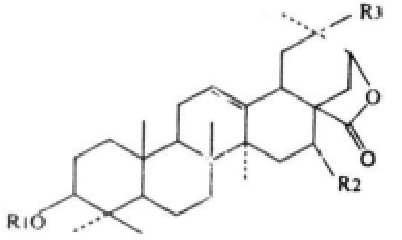

II

Figure S49 Chemical structure of compounds 1-41

Table S18 Chemical Structures of compounds 1-41

cpd	Name	Mother nucleus	Substituents
1	Julibroside J_1	I	R_1 :xyl $\overset{2}{—}$ara $\overset{6}{—}$glc :R_2 : OH :R_3 :glc $\overset{3}{—}$(araf $\overset{4}{—}$)-rha $\overset{2}{—}$glc :R_4 :qui $\overset{6}{—}$B(6S)$\overset{4}{—}$ qui $\overset{6}{—}$A (6S) :R_5 :CH_3
2	Julibroside J_2	I	R_1 , R_2 , R_3 , R_5 同 J_1 :R_4 :A(6S)$\overset{4}{—}$ qui $\overset{6}{—}$A (6S)
3	Julibroside J_3	I	R_1 :xyl $\overset{2}{—}$ara $\overset{6}{—}$2-acetamido-2-deoxy-glc :R_2 , R_3 , R_4 , R_5 同 J_1
4	Julibroside J_4	I	R_1 :xyl $\overset{2}{—}$fuc $\overset{6}{—}$glc :R_2 , R_3 , R_5 同 J_1 :R_4 :qui $\overset{6}{—}$B(6S)$\overset{4}{—}$ qui $\overset{6}{—}$B (6S)
5	Julibroside J_5	I	R_1 , R_2 , R_3 , R_5 同 J_4 :R_4 :qui $\overset{6}{—}$B(6R)$\overset{4}{—}$ qui $\overset{6}{—}$A (6S)
6	Julibroside J_6	I	R_1 , R_2 , R_3 , R_5 同 J_3 :R_4 同 J_2
7	Julibroside J_7	I	R_1 , R_2 , R_3 , R_5 同 J_1 :R_4 :A(6S)$\overset{3}{—}$ quiA $\overset{6}{—}$ (6S)
8	Julibroside J_8	I	R_1 , R_2 , R_3 , R_5 同 J_4 :R_4 :qui $\overset{6}{—}$B(6S)$\overset{4}{—}$ qui $\overset{6}{—}$A (6S)
9	Julibroside J_9	I	R_1 , R_2 , R_3 , R_5 同 J_1 :R_4 :qui $\overset{6}{—}$B(6S)$\overset{4}{—}$ qui $\overset{6}{—}$A (6R)
10	Julibroside J_{10}	I	R_1 , R_2 , R_3 , R_5 同 J_1 :R_4 :qui $\overset{6}{—}$B(6R)$\overset{2}{—}$ qui $\overset{6}{—}$A (6S)
11	Julibroside J_{11}	I	R_1 , R_2 , R_3 , R_5 同 J_1 :R_4 :qui $\overset{6}{—}$B(6S)$\overset{2}{—}$ qui $\overset{6}{—}$A (6S)
12	Julibroside J_{12}	I	R_1 :xyl $\overset{2}{—}$fuc $\overset{6}{—}$2-acetamido-2-deoxy-glc :R_2 , R_3 , R_5 同 J_1 :R_4 :qui $\overset{6}{—}$B(6R)$\overset{4}{—}$ qui $\overset{6}{—}$A (6S)
13	Julibroside J_{13}	I	R_1 , R_2 , R_3 , R_5 同 J_{12} :R_4 :qui $\overset{6}{—}$B(6S)$\overset{4}{—}$ qui $\overset{6}{—}$A (6S)
14	Julibroside J_{14}	I	R_1 , R_2 , R_3 , R_5 同 J_1 :R_4 :qui $\overset{6}{—}$B(6R)$\overset{4}{—}$ qui $\overset{6}{—}$B(6S)
15	Julibroside J_{15}	I	R_1 , R_2 , R_3 , R_5 同 J_1 :R_4 :qui $\overset{6}{—}$B(6S)$\overset{4}{—}$ qui $\overset{6}{—}$B(6S)
16	Julibroside J_{20}	I	R_1 , R_2 , R_3 , R_5 同 J_1 :R_4 :xyl $\overset{6}{—}$A(6S)
17	Julibroside J_{22}	I	R_1 , R_2 , R_3 , R_5 同 J_1 :R_4 :qui $\overset{6}{—}$A(6S)
18	Julibroside J_{23}	I	R_1 , R_2 , R_3 , R_5 同 J_4 :R_4 :xyl $\overset{6}{—}$B(6R)$\overset{4}{—}$ qui $\overset{6}{—}$A(6S)
19	Julibroside J_{24}	I	R_1 , R_2 , R_3 , R_5 同 J_1 :R_4 :A(6S)$\overset{3}{—}$ xyl $\overset{6}{—}$A(6S)
20	Julibroside J_{25}	I	R_1 , R_2 , R_3 R_5 同 J_3 :R_4 :qui $\overset{6}{—}$A(6S)
21	Julibroside J_{26}	I	R_1 , R_2 , R_3 , R_5 同 J_1 :R_4 :qui $\overset{6}{—}$A(6S)
22	Julibroside J_{27}	I	R_1 , R_2 , R_3 , R_5 同 J_4 :R_4 : 同 J_2
23	Julibroside J_{28}	I	R_1 , R_2 , R_3 , R_5 同 J_{12} :R_4 : 同 J_{14}

cpd	Name	Mother nucleus	Substituents
24	Julibroside JⅠ	Ⅰ	R_1:xyl $\overset{2}{-}$fuc $\overset{6}{-}$(glc $\overset{2}{-}$)-glc;R_2, R_3, R_5 同 J_1;R_4:qui $\overset{6}{-}$B $\overset{4}{-}$qui $\overset{6}{-}$B
25	Julibroside JⅡ	Ⅰ	R_1, R_2, R_3, R_5 同 J_4;R_4:同 JⅠ
26	Julibroside JⅢ	Ⅰ	R_1, R_2, R_3, R_5 同 J_{12};R_4:同 JⅠ
27	Prosapogenin1	Ⅰ	R_1, R_2, R_5 同 J_4;R_3:H;R_4:OH
28	Prosapogenin2	Ⅰ	R_1, R_2, R_5 同 J_3;R_3:H;R_4:OH
29	Prosapogenin3	Ⅱ	R_1:xyl $\overset{2}{-}$ara $\overset{6}{-}$glc;R_2:OH;R_3:CH_3
30	Prosapogenin4	Ⅱ	R_1:xyl $\overset{2}{-}$ara $\overset{6}{-}$2-acetamido-2-deoxy-glc;R_2:OH;R_3:CH_3
31	Prosapogenin5(Julibroside A_1)	Ⅱ	R_1:xyl $\overset{2}{-}$fuc $\overset{6}{-}$(glc $\overset{2}{-}$)-glc;R_2:OH;R_3:CH_3
32	Prosapogenin6(Julibroside A_2)	Ⅱ	R_1:xyl $\overset{6}{-}$fuc $\overset{6}{-}$glc;R_2:OH;R_3:CH_3
33	Prosapogenin7(Julibroside A_3)	Ⅱ	R_1:xyl $\overset{2}{-}$fuc $\overset{6}{-}$2-acetamido-2-deoxy-glc;R_2:OH;R_3:CH_3
34	Prosapogenin8	Ⅰ	R_1, R_2, R_3, R_5 同 JⅠ;R_4:qui $\overset{6}{-}$B
35	Prosapogenin9	Ⅰ	R_1, R_2, R_3, R_5 同 J_4;R_4:qui $\overset{6}{-}$B
36	Prosapogenin10	Ⅰ	R_1, R_2, R_3, R_5 同 J_4;R_4:qui $\overset{6}{-}$A
37	Prosapogenin11	Ⅰ	R_1, R_3, R_5 同 J_4;R_2:H;R_4:qui $\overset{6}{-}$B
38	Prosapogenin12	Ⅰ	R_1, R_3, R_5 同 J_1;R_2:H;R_4:qui $\overset{6}{-}$B
39	Julibroside A_4	Ⅱ	R_1:glc $\overset{4}{-}$glc;R_2:-O-glc;R_3:CH_3
40	Julibroside B_1	Ⅱ	R_1:glc $\overset{4}{-}$glc;R_2:-O-glc;R_3:CH_2OH
41	Julibroside C_1	Ⅱ	R_1:xyl $\overset{2}{-}$fuc $\overset{6}{-}$(glc $\overset{2}{-}$)-glc;R_2:OH;R_3:COOH

Polygala tenuifolia willd

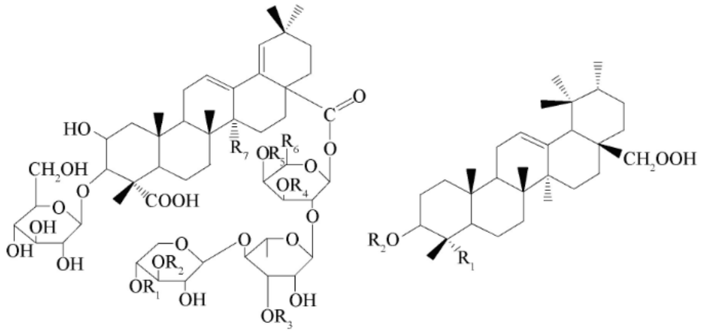

母核A　　母核B

Figure S50 Chemical structure of compounds 1-38

Table S19 Chemical Structures of compounds 1-38

cpd	name	Substituents							
		Mother nucleus	R1	R2	R3	R4	R5	R6	R7
1	Onjisaponin A	A	Gal	H	Api	Rha	a	CH_3	CH_2OH
2	Onjisaponin B	A	Gal	H	H	Rha	a	CH_3	CH_2OH
3	Onjisaponin E	A	Gal	H	H	H	b	CH_3	CH_2OH
4	Onjisaponin F	A	H	Ara	Api	H	b	CH_3	CH_2OH
5	Onjisaponin G	A	H	H	Api	H	b	CH_3	CH_2OH
6	Onjisaponin J	A	H	Ara	e	Rha	a	COOH	CH_3
7	Onjisaponin L	A	Gal	H	e	Rha	a	COOH	CH_3
8	Onjisaponin O	A	Gal	H	H	Rha	b	COOH	CH_3
9	Onjisaponin R	A	Gal	H	Api	H	b	COOH	CH_3
10	Onjisaponin S	A	H	Ara	Api	Rha	b	COOH	CH_3
11	Onjisaponin T	A	H	Ara	Api	Glc6Od	b	COOH	CH_3
12	Onjisaponin V	A	Gal	H	e	b	H	COOH	CH_3
13	Onjisaponin W	A	H	Ara	e	b	H	COOH	CH_3
14	Onjisaponin X	A	H	Ara	e	Gal	b	COOH	CH_3
15	Onjisaponin Y	A	H	H	H	Rha	a	COOH	CH_3
16	Onjisaponin Z	A	H	H	H	Rha	a	COOH	CH_3
17	Onjisaponin Vg	A	Gal	H	e	H	b	COOH	CH_3
18	Onjisaponin Pg	A	Gal	H	e	H	H	COOH	CH_3
19	Onjisaponin Gg	A	H	H	e	H	b	COOH	CH_3
20	Onjisaponin Fg	A	H	Ara	e	H	b	COOH	CH_3
21	Onjisaponin Qg	A	Gal	H	e	Rha	H	COOH	CH_3
22	Onjisaponin Ng	A	H	H	e	Rha	a	COOH	CH_3
23	Onjisaponin Sg	A	H	Ara	e	Rha	b	COOH	CH_3
24	Onjisaponin Ug	A	H	Ara	e	Glc	b	COOH	CH_3
25	Onjisaponin Tg	A	H	Ara	e	Glc6Od	b	COOH	CH_3
26	Onjisaponin Wg	A	Gal	Ara	Api	b	H	COOH	CH_3
27	*E*-onjisaponin H	A	H	H	Api	Rha	a	CH_3	CH_2OH
28	*Z*-onjisaponin H	A	H	H	Api	Rha	k	CH_3	CH_2OH
29	Sibricasaponins A	B	COOH	g	-	-	-	-	-
30	Sibricasaponins B	B	H	m	-	-	-	-	-
31	Sibricasaponins C	B	H	n	-	-	-	-	-
32	Sibricasaponins E	A	Gal	H	H	x	$COCH_3$	CH_3	-
33	Tenuioflisaponin A	A	Gal6Oy	H	Api	Api	MC	CH_3	CH_2OH
34	Tenuioflisaponin B	A	Gal6Oh	H	Api	Api	MC	CH_3	CH_2OH
35	Polygalasaponin ⅩⅩⅧ	A	H	H	H	H	H	CH_3	CH_2OH
36	Polygalasaponin ⅩⅩⅫ	A	H	H	H	H	H	CH_3	CH_2OH
37	Senegin Ⅲ	A	Gal	H	H	Rha	MC	CH_3	CH_2OH
38	Senegin IV	A	Gal	H	Api	Rha	MC	CH_3	CH_2OH

注：a-(E)-4-methoxy cinanamoyl; b-(E)-3,4,5-trimethoxy cinnamoyl; e-3-hydroxy-3-methyl-5-pentanoic acid ester-5-β-D-apiofur-anosyl; g-D-glucopyranuronic acid; k-(Z)-4-methoxy cinnamoyl; m-α-D-xyl4SO3H; n-β-D-xyl-5SO3H; x-D-api ose; y-4-oxide-2-hydroxy-pentanoic acid ester-6-β-D-gal; h-2-hydroxy-4-methyl-pentanoic acid ester-6-β-D-gal; MC-p-methoxycinnamoyl; Api-β-D-apiofuranosy

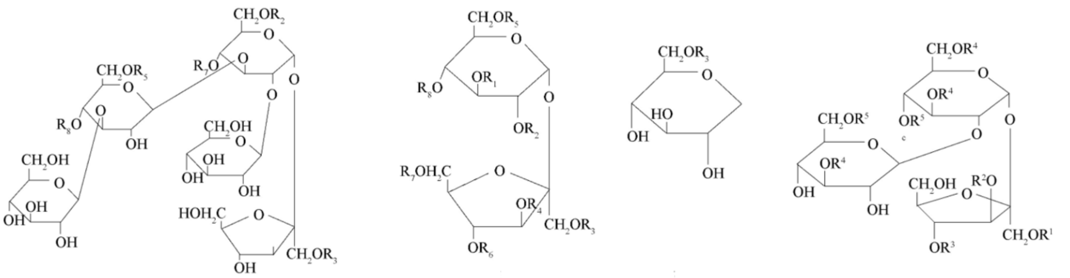

Mother nucleus A Mother nucleus B Mother nucleus C Mother nucleus D

Figure S51 Chemical structure of compounds 39-69

Table S20 Chemical Structures of compounds 39-69

cpd	name	Mother nucleus	Substituents							
			R1	R2	R	R4	R5	R6	R7	R8
39	Tenuifolioses A	A	-	a	e	b	a	-	i	a
40	Tenuifolioses B	A	-	H	e	b	a	-	i	a
41	Tenuifolioses C	A	-	H	e	b	a	-	i	H
42	Tenuifolioses D	A	-	a	e	b	a	-	i	H
43	Tenuifolioses E	A	-	a	e	b	a	-	i	H
44	Tenuifolioses F	A	-	a	e	b	a	-	m	a
45	Tenuifolioses G	A	-	a	e	b	a	-	m	H
46	Tenuifolioses H	A	-	a	e	b	a	-	e	a
47	Tenuifolioses I	A	-	a	e	b	a	-	e	H
48	Tenuifolioses J	A	-	H	e	b	a	-	e	a
49	Tenuifolioses K	A	-	H	e	b	a	-	e	H
50	Tenuifolioses L	A	-	a	e	b	a	-	n	a
51	Tenuifolioses M	A	-	a	e	b	a	-	n	H
52	Tenuifolioses N	A	-	a	i	b	a	-	i	a
53	Tenuifolioses O	A	-	H	i	b	a	-	i	a
54	Tenuifolioses P	A	-	H	i	b	a	-	i	H
55	Tenuifolioses Q	A	-	H	e	b	a	-	n	a
56	Tenuifoliside A	B	H	H	H	l	g	H	H	H
57	Tenuifoliside B	B	H	H	H	j	g	H	H	H
58	Tenuifoliside C	B	H	H	H	j	j	H	H	H
59	Tenuifoliside D	C	-	-	l	-	-	-	-	-
60	Tenuifoliside E	B	H	a	H	h	a	H	H	a
61	Sibiricose A1	B	H	H	H	H	j	H	H	H
62	Sibiricose A2	B	H	H	H	H	l	H	H	H
63	Sibiricose A3	B	H	H	H	g	H	H	H	H
64	Sibiricose A4	B	H	H	H	j	H	H	H	j
65	Sibiricose A5	B	H	H	H	i	H	H	H	H
66	Sibiricose A6	B	H	H	H	j	H	H	H	H
67	Sibiricose A7	C	-	-	x	-	-	-	-	-
68	3,6′-Disinapoylsucrose	B	H	H	H	j	j	H	H	H
69	Polygalatenosides B	D	b	-	-	-	-	-	-	-

注：a-acetyl; b-benzoyl; d-p-methylbenzoyl; e-(E)-p-coumaroyl; g-p-hydroxylbenzoyl; j-(E)-sinapoy; i-(E)-feruloyl; f-benzoyl; k-3,4-dimethoxylcinnamoyl; l-(E)-3,4,5-trimethoxycinnamoyl; m-4-O-α-L-rhamnopyranosyl-(E)-feruloyl; n-4-O-α-L-rhamnopyranosyl-(E)-p-coumaroyl; p-3,4-dimethoxylcinnamoyl; q-cinnamoyl; x-4-hydroxy-3,5-dimethoxycinnamoyl

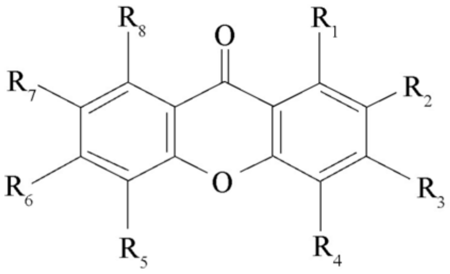

Mother nucleus A

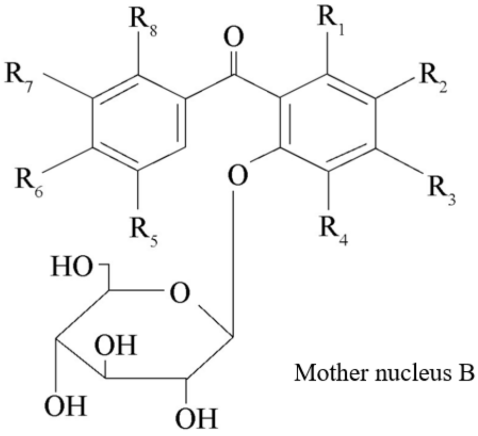

Mother nucleus B

Figure S52 Chemical structure of compounds 70-107

Table S21 Chemical Structures of compounds 70-107

cpd	name	Mother nucleus	Substituents							
			R1	R2	R	R4	R5	R6	R7	R8
70	lancerin	A	H	H	OH	H	H	OH	Glc	H
71	onjixanthone Ⅰ	A	OMe	OMe	OMe	H	H	H	OH	H
72	onjixanthone Ⅱ	A	OH	OMe	OH	H	H	OH	OMe	H
73	polygalaxanthone III	A	OH	Glc6-1Api	OH	H	H	OH	OMe	H
74	polygalaxanthone IV	A	OH	H	OMe	-	-	OGlc2-1Rha	OMe	H
75	polygalaxanthone V	A	OH	H	OH	-	-	OGlc2-1Rha	OMe	H
76	Polygalaxanthone VI	A	OMe	OMe	OMe	-	-	OGlc	OMe	H
77	polygalaxanthone VII	A	OH	OMe	Glc2-1	OH	OMe	H	H	H
78	sibiriphenone A	B	OH	H	OH	H	H	OH	OMe	H
79	sibiricaxanthone A	A	OH	Glc6-1Api	OH	H	H	H	OH	H
80	sibiricaxanthone B	A	OH	Glc2-1Rha	OH	H	H	H	OH	H
81	1,2,3-trimethoxy-7-hydr	A	OMe	OMe	OMe	H	H	H	OH	H
82	1,3,6-trihydroxy-2,7-din	A	OH	OMe	OH	H	H	OH	OMe	H
83	1,2,7-trimethoxy-3-hydr	A	OMe	OMe	OH	H	H	H	OMe	H
84	1,2,3,7-tetramethoxyxan	A	OMe	OMe	OMe	H	H	H	OMe	H
85	1,7-dihydroxy-3-metho	A	OH	H	OMe	H	H	H	OH	H
86	1,7-dihydroxy-2,3-dime	A	OH	OMe	OMe	H	H	H	OH	H
87	1,7-dihydroxy-2,3-meth	A	OMe	OCH_2O	OCH_2C	H	H	H	H	OMe
88	1,7-dihydroxy-2,3-di-m	A	OH	OCH_2O	OCH_2C	H	H	H	OH	H
89	6-hydroxy-2,3,6,7-tetrar	A	H	OMe	OMe	H	H	OMe	OMe	H
90	6-hydroxy-1,2,3,7-tetrar	A	OMe	OMe	OMe	H	H	OGlc	OMe	H
91	1,7-dihydroxyxanthone	A	OH	H	H	H	H	H	OH	H
92	1,7-dimethoxyxanthone	A	OMe	H	H	H	H	H	OMe	H
93	1-hydroxy-3,7-dimetho	A	OH	H	OMe	H	H	H	OMe	H
94	1-hydroxy-3,6,7-trimeth	A	OH	H	OMe	H	H	OMe	OMe	H
95	6,8-dihydroxy-1,2,4-trin	A	OMe	OMe	H	OH	H	H	H	OH
96	6,8-dihydroxy-1,2,3-trin	A	OMe	OMe	OMe	H	H	H	H	OH
97	1,2,3,6,7-pentamethoxy	A	OMe	OMe	OMe	H	H	OMe	OMe	H
98	1,3,7-trihydroxyxanthon	A	OH	H	OH	H	H	H	OH	H
99	1,6,7-trihydroxy-2,3-din	A	OH	OMe	OMe	H	H	OH	OH	H
100	3-hydroxy-2,8-dimetho	A	H	OMe	OH	H	H	H	H	OMe
101	7-hydroxy-1,2,3-trimeth	A	OMe	OMe	OMe	H	H	H	OH	H
102	3-hydroxy-1,2,7-trimeth	A	OMe	OMe	OH	H	H	H	OMe	H
103	2,3,8-trimethoxyxanthor	A	H	OMe	OMe	H	H	H	H	OMe
104	1,3,6,7-tetramethoxyxan	A	OMe	H	OMe	H	H	OMe	OMe	H
105	1,3,7-trimethoxyxanthor	A	OMe	H	OMe	H	H	H	OMe	H
106	7-*O*-methylmangiferin	A	OH	Glc	OH	H	H	OH	OMe	H
107	euxanthone	A	OH	H	H	H	H	H	OH	H

Albizia julibrissin durazz bark

Table S22 Chemical Structures of compounds 1-10

Cpd	Name
1	Quercitrin
2	Kaempferol
3	Quercetin
4	Kaempferol-3-O-α-L-rhamnoside
5	Luteolin
6	Isorhamnetin
7	Isoquercetin
8	Rutin
9	Myricetin
10	adianthifolioside A

Figure S53 Chemical structure of compounds 1-10

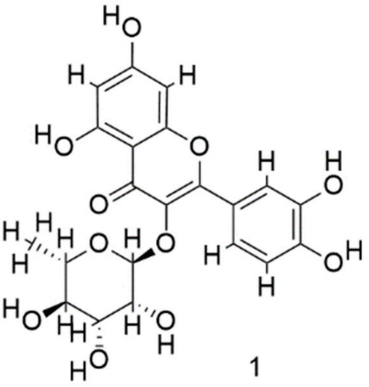

1

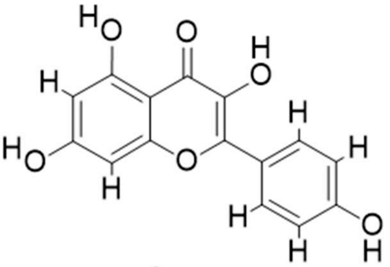

2

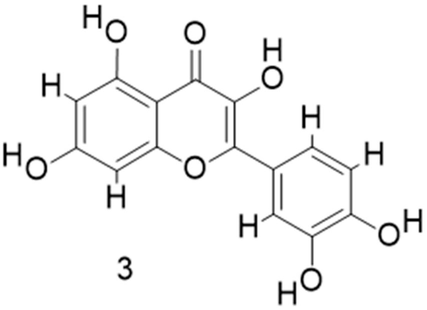

3

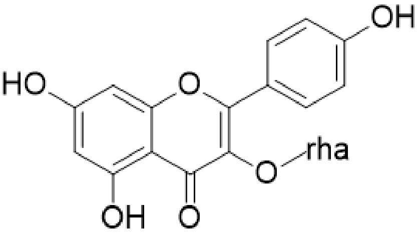

4

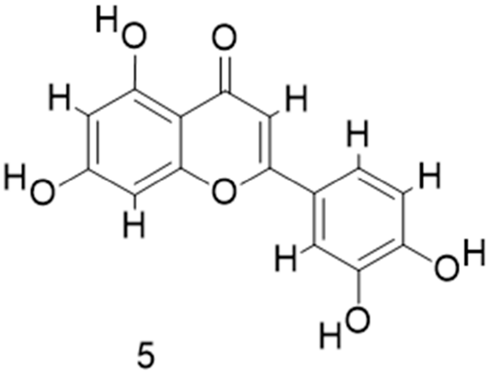

5

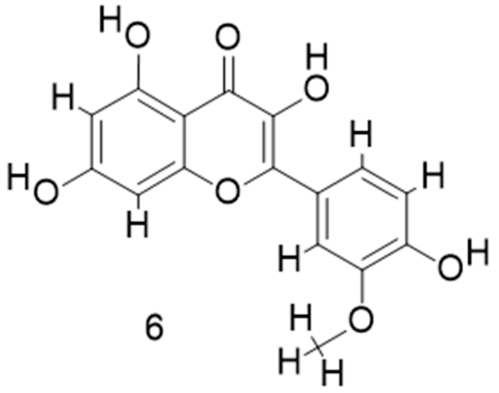

6

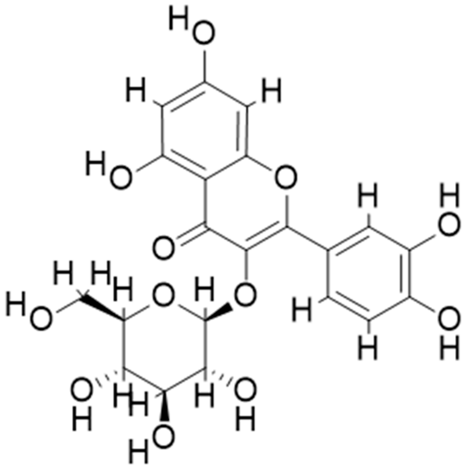

7

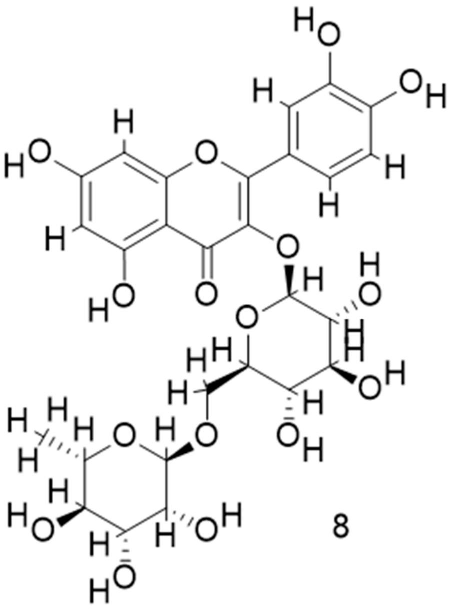

8

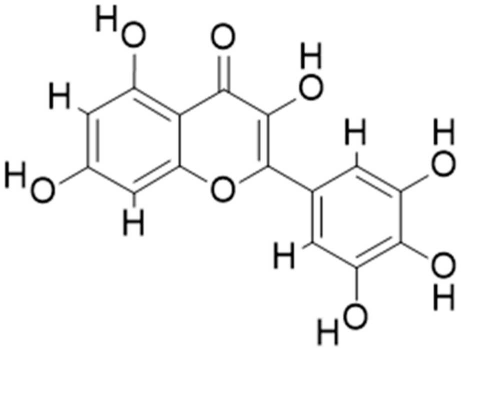

9

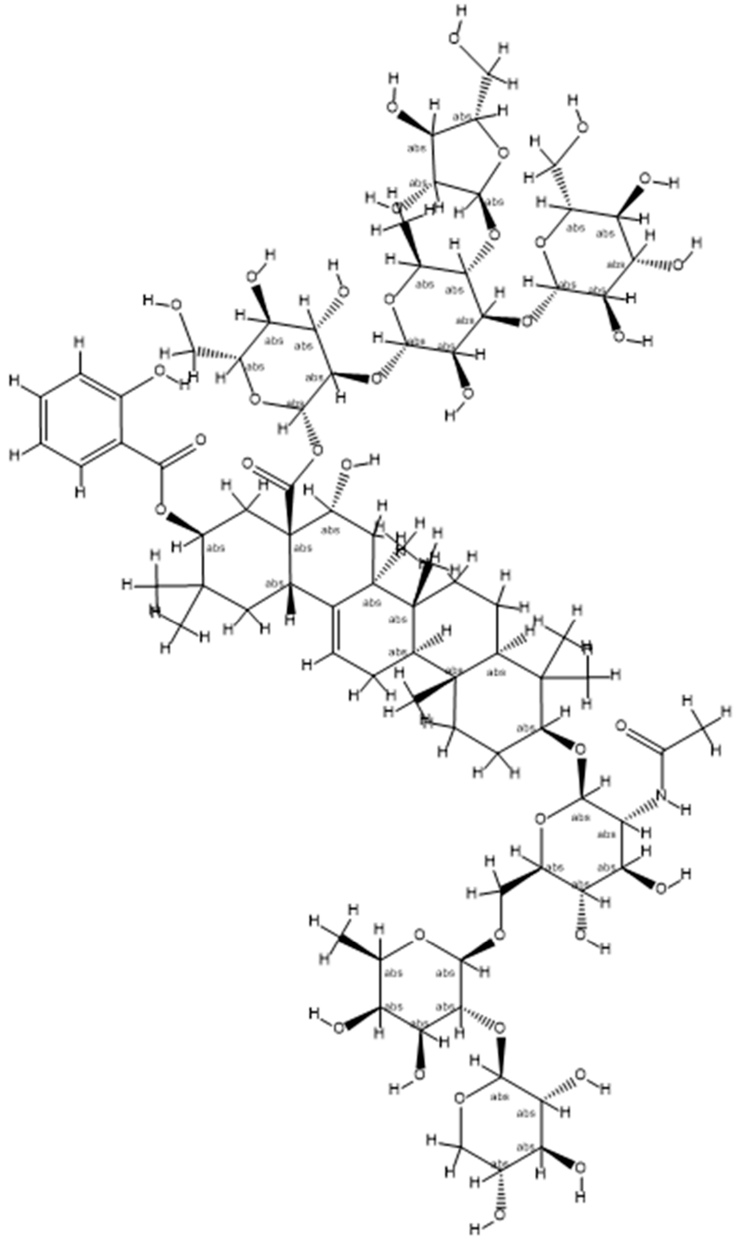

10

Apocynum Venetum

Table S23 Chemical composition of *Apocynum* Venetum

NO.	compound	NO.	compound
**1**	Quercetin	**27**	Acetylated hypericin
**2**	Kaempferol	**28**	Neoisorutin
**3**	Isorhamnetin	**29**	4’-o-methylquercetin
**4**	Quercetin 3-O-(6"-acetyl-glucoside)	**30**	Quercetin-3-O-β-D-glucoside
**5**	Rutin	**31**	kaempferol-3-O-β-D-glucoside

**6**	Isoquercetin	**32**	Quercetin-3-O-β-D-glucopyranyl(2→1)-O-β-D-grapea pyranoside
**7**	Quercetin 3-O-malonylglucoside	**33**	(+)-Catechin
**8**	Quercetin-3-O-Sophoroside	**34**	(-)-Epicatechin
**9**	Trifolin	**35**	(-)-Epigallocatechin
**10**	kaempferol-3-O-(6’’-O-acetyl)-β-D-glucopyranoside	**36**	apocyninA
**11**	quercetin-3-O-（6’’-O acetyl）-β-D-glucopyranoside	**37**	apocynin B
**12**	quercetin-3-O-（6″-O-acetyl）-β-D-galactopyranosid	**38**	apocynin D
**13**	kaempferol-7-O-α-L-rhamnopyranoside	**39**	Trans-2-Penten-1-Ol
**14**	2"-Acetylhyperin	**40**	4,4-Dimethyl-2-cyclopenten-1-one
**15**	Quercetin-3-O-beta-D-glucosylgalactoside	**41**	Methylcyclohexane
**16**	Amentoflavone	**42**	2-Methyldihydro-3(2H)-furanone
**17**	Quercetin 3-glucoside	**43**	1,2,5,5-Tetramethyl-1,3-cyclopentadiene
**18**	Quercetin-3-O-glucuronic acid	**44**	n-3-Hexenol
**19**	Quercetin-3-O-(6"-O-malonyl)-galactoside	**45**	p-Xylene
**20**	Quercetin 3-O-(6"-O-malonyl)-β-D-glucoside	**46**	1,3,5-Octatriene
**21**	kaempferol-3-O-(6"-O-malonyl)-glucoside	**47**	2-Furanmethanol
**22**	kaempferol-3-O-(6"-O-acetyl)-glucoside	**48**	1,3,5,7-Cyclotetraoctene
**23**	kaempferol-3-rutinoside	**49**	2-Acetylfuran
**24**	Quercetin-3-β-D-glucopyranoside	**50**	4-Ethylphenol
**25**	Baimaside	**51**	3,5-Dimethylphenol
**26**	Astragalin		

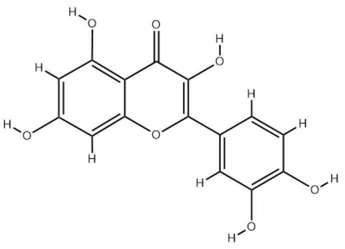

1

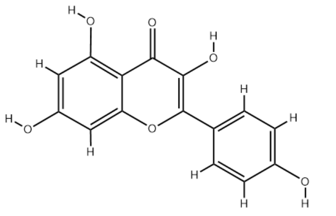

2

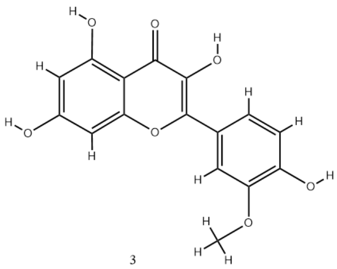

3

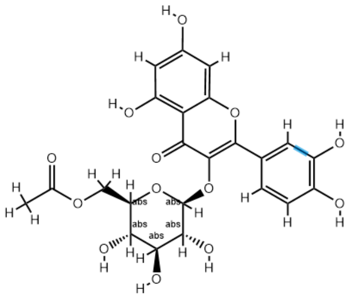

4

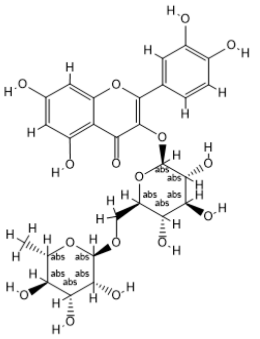

5

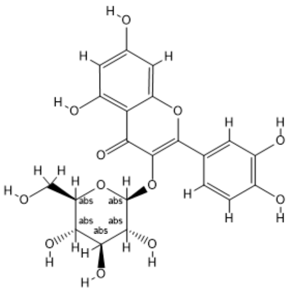

6

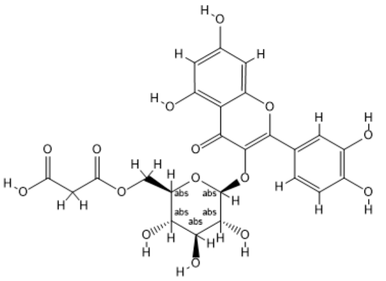

7

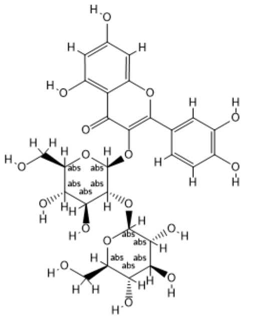

8

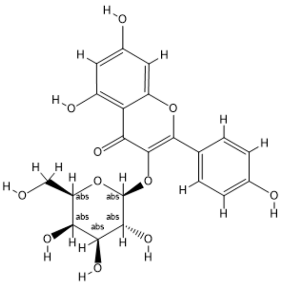

9

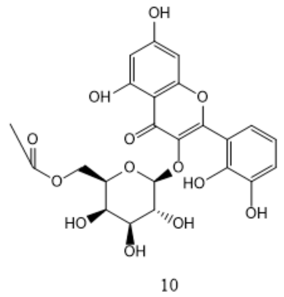

10

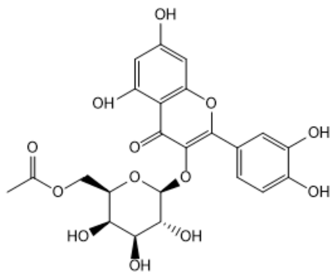

11

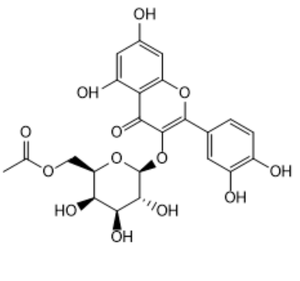

12

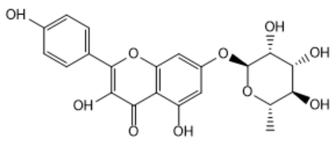

13

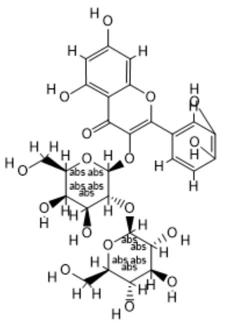

14

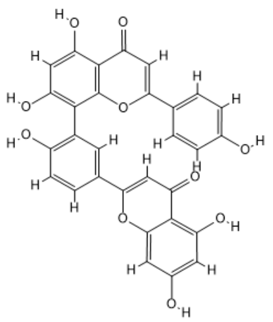

15

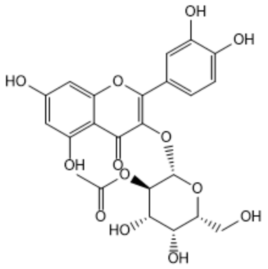

16

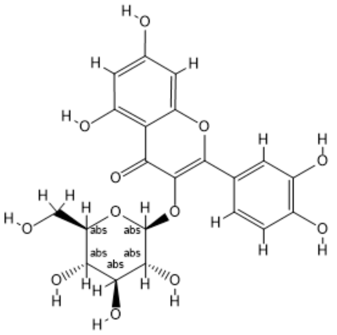

17

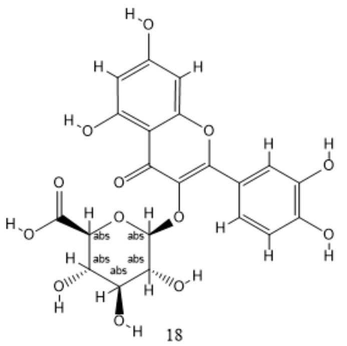

18

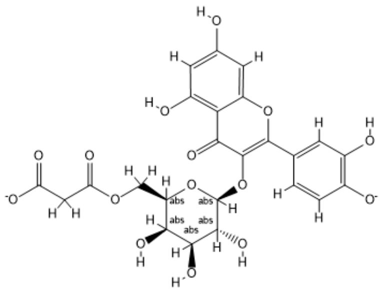

19

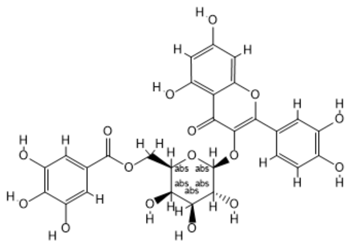

20

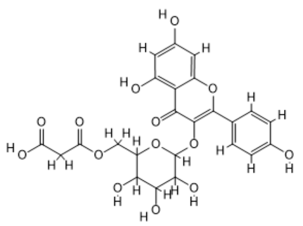

21

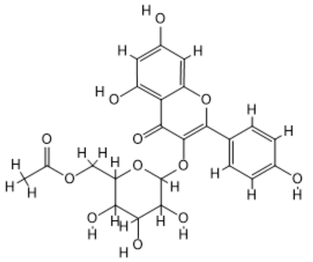

22

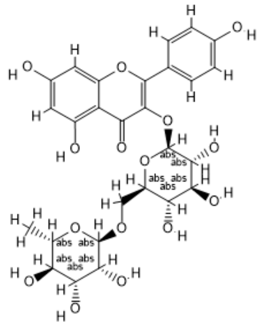

23

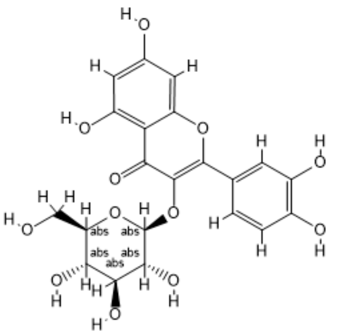

24

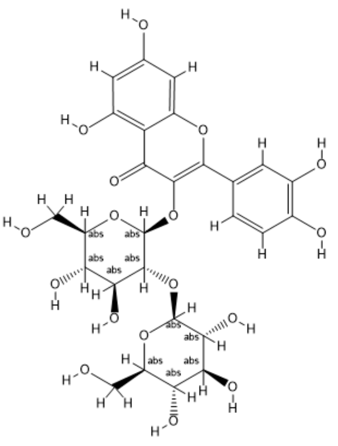

25

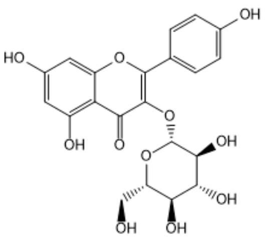

26

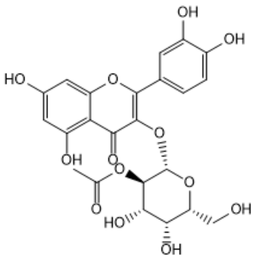

27

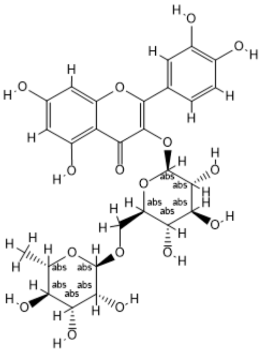

28

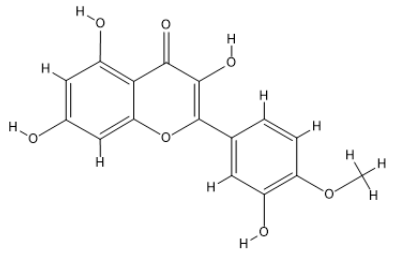

29

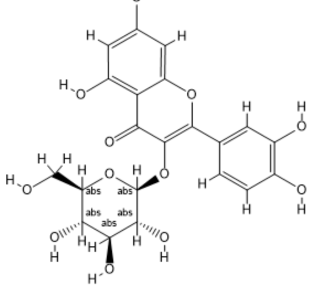

30

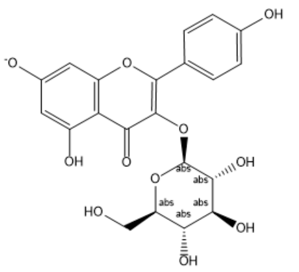

31

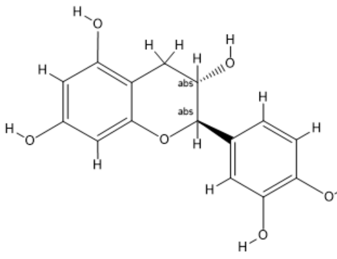

32

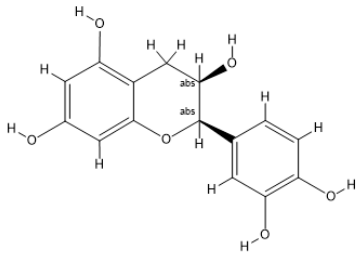

33

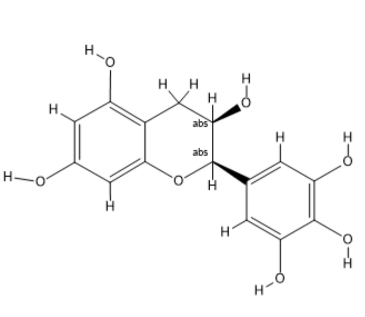

34

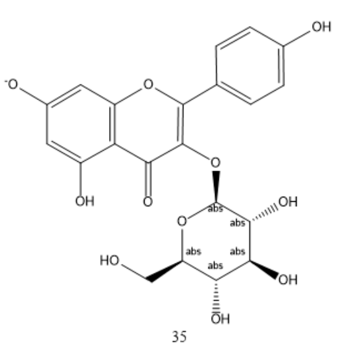

35

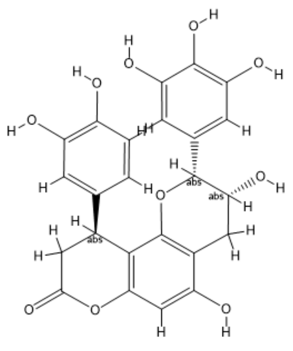

36

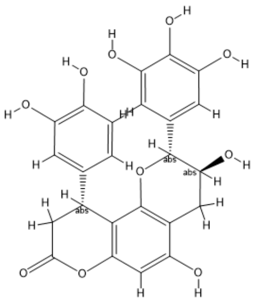

37

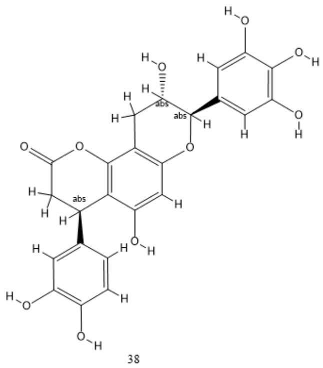

38

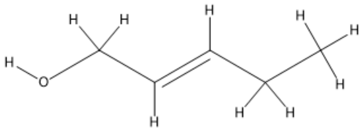

39

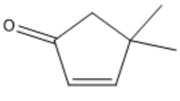

40

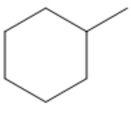

41

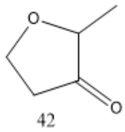

42

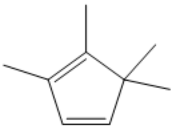

43

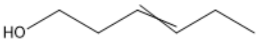

44

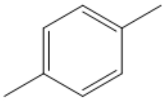

45

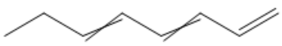

46

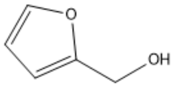

47

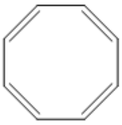

48

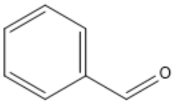

49

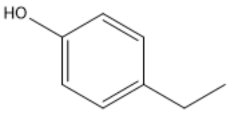

50

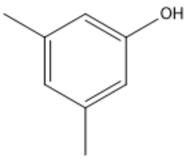

51

Figure S54.The structures of *Apocynum* Venetum

Platycladus orientalis franco

Table S24 Chemical composition of *Platycladus orientalis* franco

NO.	compound	NO.	compound
**1**	pimisolide	**11**	Methyl 10-undecylenate
**2**	a-cedrol	**12**	Methyl 9-oxonanoate
**3**	platydiol	**13**	Methyl 10-oxodecanoate
**4**	(+)cateehin	**14**	Methyl 11-oxoundecanoate
**5**	(-)-Epicatechin	**15**	Methyl tetradecanoate
**6**	Methyl heptanoate	**16**	Methyl 2-eicosenoate
**7**	Methyl 4-octenoate	**17**	Methyl eicosanate
**8**	Methyl octanoate	**18**	linoleic acid
**9**	Methyl nonanoate	**19**	linolenic acid
**10**	Methyl 8-oxooctanoate	**20**	arachidonic acid

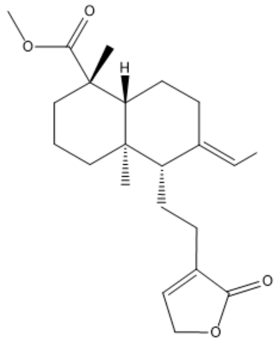

1

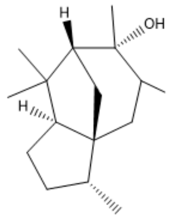

2

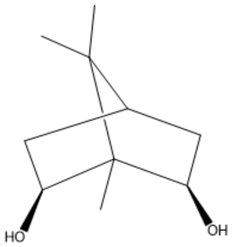

3

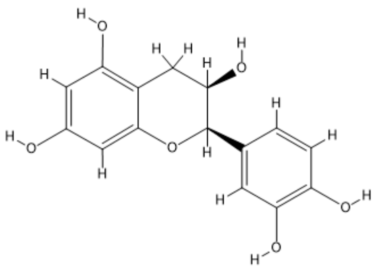

4

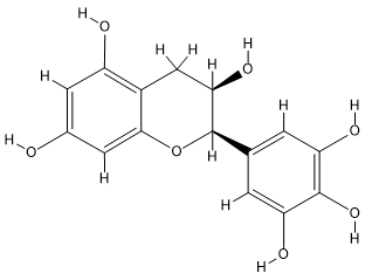

5

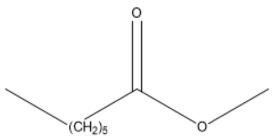

6

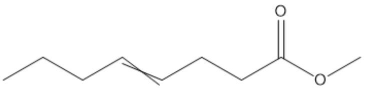

7

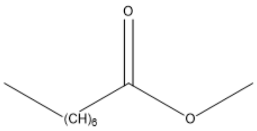

8

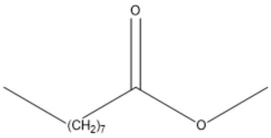

9

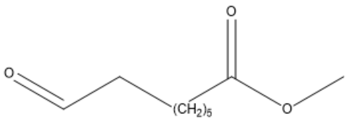

10

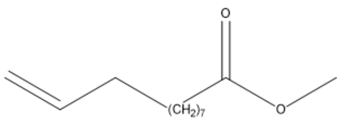

11

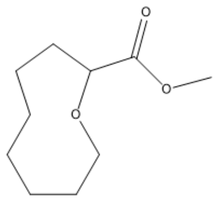

12

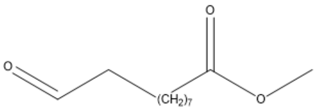

13

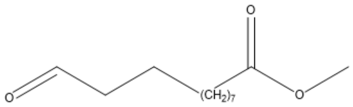

14

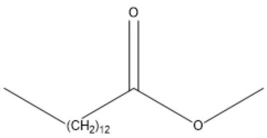

15

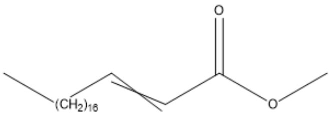

16

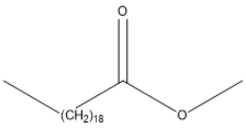

17

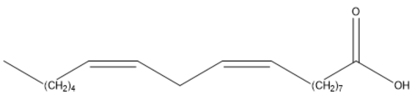

18

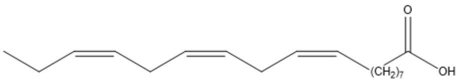

19

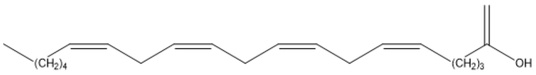

20

Figure S55.The structures of *Platycladus orientalis* franco

Polygonum multiflorum thumb

Table S25 Chemical composition of *Polygonum multiflorum* thumb

NO.	compound	NO.	compound
**1**	2,5-dimethyl-7-hydroxychromone	**30**	2,5-dimethyl-7-hydroxychromone-7-O-β-D-glucopyranoside
**2**	emodin	**31**	noreugenin

**3**	catechin	**32**	protocatechuicacid
**4**	physcion-8-O-β-D-glucopyranosideglucopyranoside	**33**	methylgallate
**5**	torachrysone-8-O-β-D-glucopyranoside	**34**	quercetin-3-O-β-D-glucopyranoisde
**6**	emodin-8-O-β-D-glucopyranoside	**35**	noreugenin-7-O-β-D-glucopyranoside
**7**	2,3,5,4-tetrahydroxystilbene-2-O-β-D-glucopyranoside	**36**	2,5-dimethyl-7-hydroxychromone
**8**	W-Hydroxyemodin	**37**	kaempferol
**9**	Aloe emodin	**38**	genistin
**10**	Emodin-5-O-xyloside	**39**	hyperin
**11**	Emodin-1-O-β-D-glucose	**40**	caffeicacid
**12**	Emodin-8-O- (6'-O-malonyl)-β-D-glucoside	**41**	dodecane
**13**	Emodin methyl ether-8-O-β-D-gentiandiglycoside	**42**	tridecane
**14**	Palmarula anthrone A	**43**	tetradecane
**15**	3,6-Dimethoxy-2,5,7,8-tetrahydroxynaphthoquinone	**44**	daucosterol
**16**	Quercetin	**45**	β-sitosterol
**17**	Luteolin-3-O-xyloside	**46**	Stilbene glycosides
**18**	Apigenin	**47**	Stilbene-O-dihexanoside
**19**	Luteolin	**48**	Stilbene glucoside
**20**	Vitex	**49**	coumaric acids
**21**	Apigenin-7-glucoside	**50**	Galic acid-3-O-glucoside
**22**	Rutin	**51**	Protocornflower B5
**23**	Viosterin	**52**	Diisobutyl phthalate
**24**	Piperin	**53**	Dibutyl phthalate
**25**	Epicatechins	**54**	hexanal
**26**	Epicatechin-3-O-gallate	**55**	6,10,14-Trimethyl-2-pentadecanone
**27**	Catechin-5-O-β-D-glucoside	**56**	Butyl butyrate
**28**	Kaempferol-3-β-D-glucoside	**57**	Nonanaldehyde
**29**	2-(2'-hydroxypropyl)-5-carboxymethyl-7-hydroxychromone		

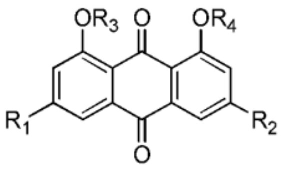

**1**	R_1=CH_3	R_2=OH	R_3=H	R_4=H
**2**	R1=CH_3	R_2=OCH_3	R_3=H	R_4=H
**3**	R_1=CH_2OH	R_2=OH	R_3=H	R_4=H
**4**	R_1=CH_3	R_2=H	R_3=H	R_4=H
**5**	R_1=CH_2OH	R_2=H	R_3=H	R_4=H
**6**	R_1=COOH	R_2=H	R_3=H	R_4=H
**7**	R_1=CH_3	R_2=H	R_3=H	R_4=Glc
**8**	R_1=CH_3	R_2=OH	R_3=Glc	R_4=H
**11**	R_1=CH_3	R_2=OH	R_3=H	R_4=CH_3

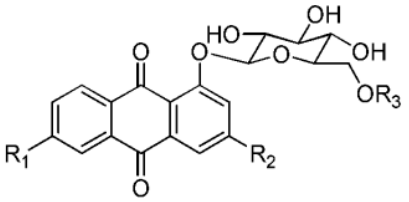

**9**	R_1=H	R_2=OCH_3	R_3=H
**12**	R_1=CH_3	R_2=OH	R_3=malonyl
**13**	R_1=CH_3	R_2=OH	R_3=Glc

Glc 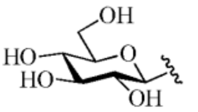

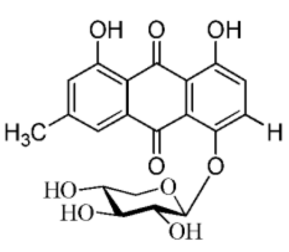

10

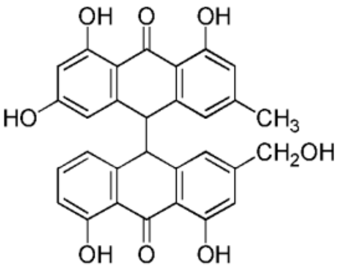

14

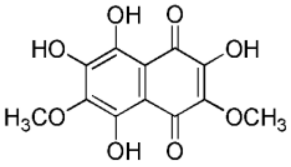

15

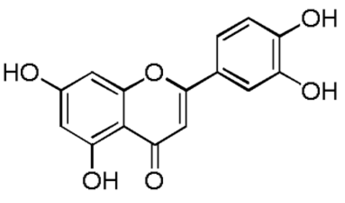

16

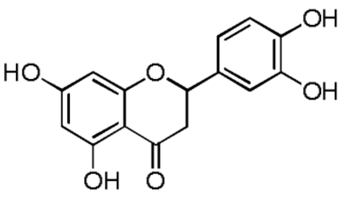

17

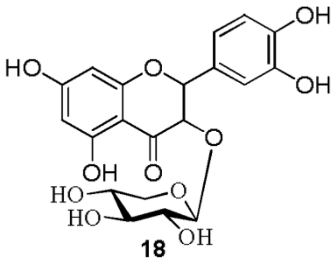

18

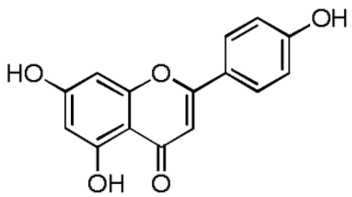

19

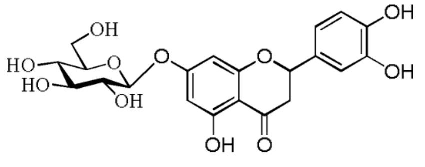

20

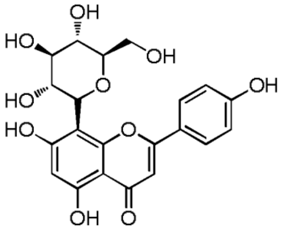

21

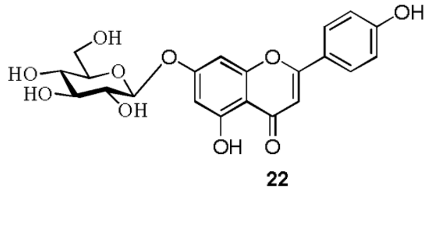

22

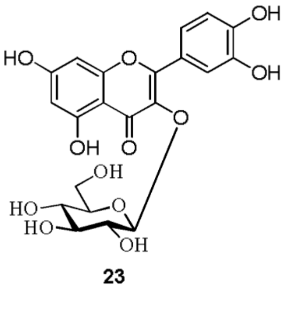

23

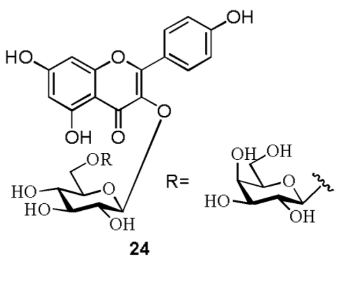

24

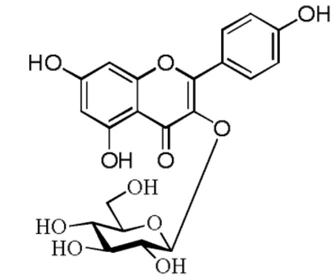
25

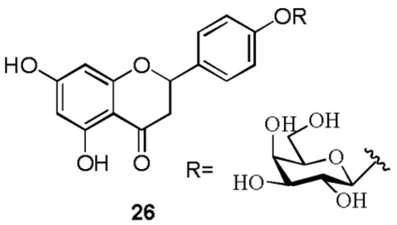
26

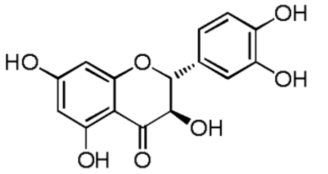
27

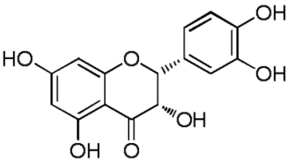
28

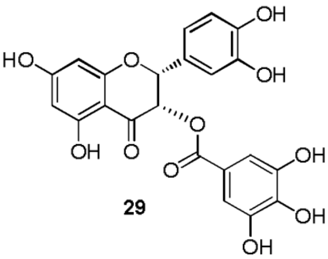
29

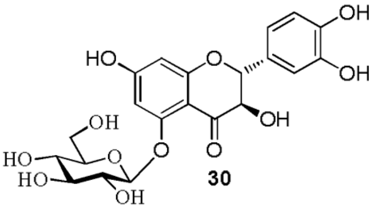
30

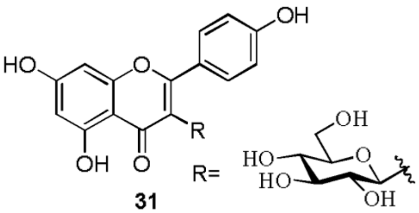
31

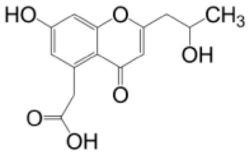
32

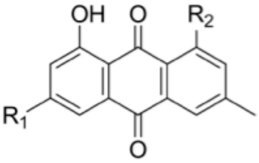

compound	R_1	R_2
33	OH	OH
34	OCH_3	H
35	OCH_3	OGlc
36	OH	OGlc

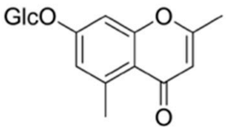
37

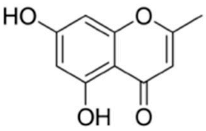
38

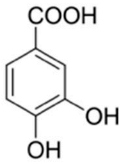
39

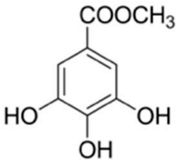
40

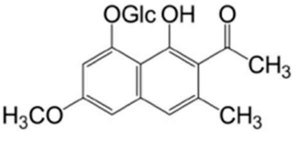
41

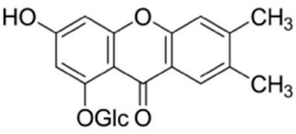
42

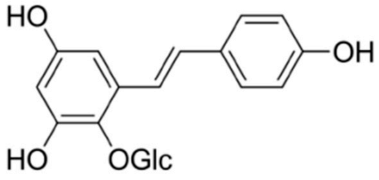

43

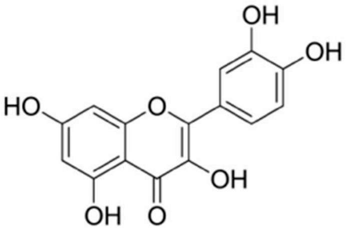

44

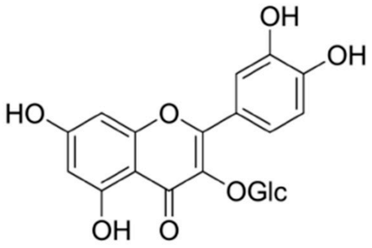

45

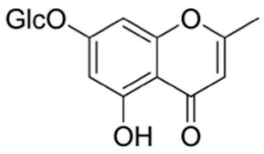

46

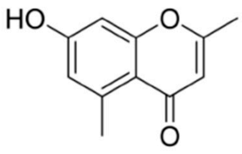

47

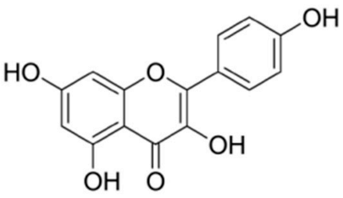

48

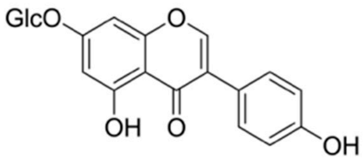

49

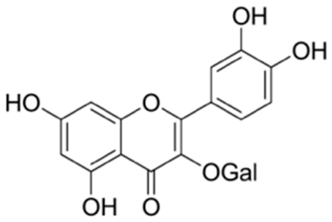

50

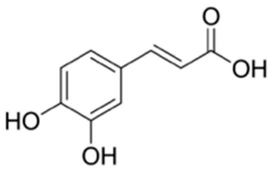

51

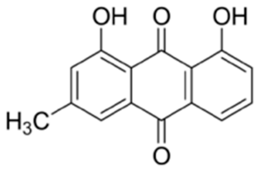

52

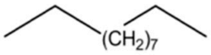

53

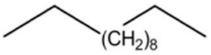

54

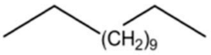

55

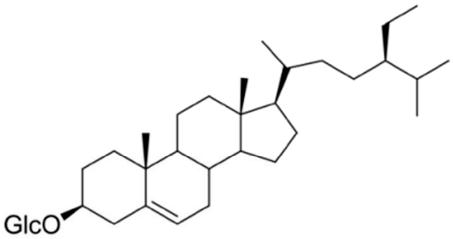

56

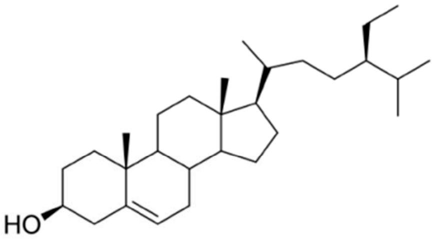

57

Figure S56.The structures of *Polygonum multiflorum* thumb

Nelumbo nucifera gaertn

Table S26 Chemical composition of *Nelumbo nucifera* gaertn

NO.	compound	NO.	compound
**1**	Luteolin 6-C-glucoside-8-C-glucoside	**63**	O-Nornuciferine
**2**	Apigenin 6-C-glucoside-8-C-glucoside	**64**	Roemerine
**3**	Luteolin 6-C-pentoside-8-C-glucoside	**65**	Nuciferine
**4**	Chrysoeriol (Diosmetin) 6-C-pentoside (hexoside)-8-C-hexoside (pentoside)	**66**	Anonaine
**5**	Luteolin 6-C-pentoside-8-C-glucoside	**67**	Coclaurine-5'-O-pentoside
**6**	Apigenin 6-C-glucoside-8-C-polyxylosidase	**68**	Oxidation-Nuciferine
**7**	Luteolin 6-C-glucoside-8-C-pentoside	**69**	Lotusine 4' -O-glucoside
**8**	Schaftoside	**70**	Norcocalurine 4' -O-glucoside
**9**	Isoorientin	**71**	N-methylnorcoclaurine 7-O-glucoside
**10**	Apigenin 6-C-polyxylosidase-8-C-glucoside	**72**	N-methylnorcoclaurine 4' -O-glucoside
**11**	Luteolin 6-C- rhamnoside-8-C-pentoside	**73**	Isolotusine 4'-O-glucoside
**12**	Orientin	**74**	Norcocalurine 6-O-glucoside

**13**	Isoschaftoside	**75**	N-methylcoclaurine 7-O-glucoside or N-methylcoclaurine 4' -O-glucoside
**14**	Luteolin 6-C-pentoside-8-C-rhamnoside	**76**	Norcoclaurine
**15**	Chrysoeriol (Diosmetin) 6-C-pentoside (hexoside)-8-C-hexoside (pentoside)	**77**	N-methylnorcoclaurine
**16**	Quercetin 3-O-rhamnoside-glucoside	**78**	Lotusine
**17**	Chrysoeriol (Diosmetin) 6-C-pentoside (hexoside)-8-C-hexoside (pentoside)	**79**	Isolotusine 4'-O-glucoside
**18**	Quercetin 3-O-rhamnopyranosyl-(1→6)-glucopyranoside (rutin)	**80**	Nornuciferidine
**19**	Isovitexin	**81**	Norisoliensinine
**20**	Quercetin 3-O-galactoside	**82**	N-methylisococlaurine
**21**	Quercetin 3-O-glucuronide	**83**	Coclaurine
**22**	Quercetin 3-O-glucoside	**84**	6-hydroxylnorisoliensinine
**23**	Kaempferol 7-O-galactoside	**85**	N-methylcoclaurine
**24**	Diosmetin 6-C-glucoside	**86**	N-norisoliensinine
**25**	Luteolin 7-O-rutinoside	**87**	Liensinine
**26**	Kaempferol 3-O-robinobiosideC27H30O15	**88**	4-methyl-N-methylcoclaurine
**27**	Isorhamnetin 3-O-neohesperidoside	**89**	Isoliensinine
**28**	Isorhamnetin 3-O-rutinoside	**90**	3-hydroxylisoliensine
**29**	Isorhamnetin 3-O-galatoside	**91**	6-demethyl-4' -methyl-N-methylcoclaurine
**30**	Isorhamnetin 3-O-glucoside	**92**	Neferine
**31**	Kaempferol 7-O-glucoside	**93**	Armepavine
**32**	Diosmetin (Chrysoeriol) 6,8-di-C-glucoside	**94**	3-hydroxylneferine
**33**	Diosmetin 8-C-glucoside	**95**	Nuciferine
**34**	Diosmetin 7-O-rutinoside	**96**	Quercetin 3-O-glucosyl -(1 → 2) -glucoside
**35**	Chrysoeriol 7-O-glucoside	**97**	Luteolin 6, 8-di-C-glucosides
**36**	Kaempferol	**98**	Luteolin 6-C-pentoside-8-C-glucoside
**37**	Quercetin	**99**	Apigenin 6, 8-di-C-glucoside (Vicenin-2)
**38**	Diosmetin	**100**	Kaempferol 3-O-neohesperidosides 7-O glucoside
**39**	Norcoclaurine-4'-O-glucoside	**101**	Luteolin 6-C-glucoside (Isoorientin)
**40**	N-methylhigenamine	**102**	Luteolin 8-C-glucoside (Orientin)
**41**	Norcoclaurine-6-O-glucoside	**103**	Apigenin 6-C-glucoside-8-C-rhamnoside (Violanthin)
**42**	Norcoclaurine	**104**	Quercetin 3-O-galactoside (Hyperoside)

**43**	Argemexirine	**105**	Hesperitin 7-O-rutinoside (Hesperidine)
**44**	Lotusine	**106**	Apigenin-6-C-glucoside (Isovitexin)
**45**	Nornuciferidine	**107**	Apigenin 6-C-rhamnoside-8-C-glucoside
**46**	Norisoliensinine	**108**	5,7,3′,4′,5′-pentahydroxyflavone (Tricetin
**47**	N-methylisococlaurine	**109**	Quercetin 3-O-glucoside (Isoquercitrin)
**48**	Isococlaurine	**110**	Apigenin-8-C-glucoside (Vitexin)
**49**	N-methylcoclaurine	**111**	Luteolin
**50**	6-Hydroxynorisoliensinine	**112**	Quercetin 3-O-glucuronide (Querciturone)
**51**	Pronuciferine	**113**	Kaempferol-3-O-rutinoside (Nicotiflorin)
**52**	Thalifoline	**114**	Quercetin
**53**	Armepavine	**115**	Kaempferol
**54**	Liensinine	**116**	Kaempferol 7-O-neohesperidoside
**55**	6-demethy-4'-methyl-N-methylcoclaurine	**117**	Isorhamnetin
**56**	Coclaurine	**118**	Isorhamnetin 3-O-neohesperidoside
**57**	Isoliensinine	**119**	Apigenin-7-O-neohesperidoside (Rhoifolin)
**58**	N-Nornuciferine	**120**	Naringenin
**59**	N-nor-O-methylarmepavine	**121**	Diosmetin
**60**	Neferine	**122**	Diosmetin 7-O-neohesperidoside (Neodiosmin)
**61**	Nornuciferidine	**123**	Kaempferol 3-O-galactoside (Trifolin)
**62**	Isococlaurine-5'-O-pentoside	**124**	Kaempferol 3-O-glucoside (Astragalin)

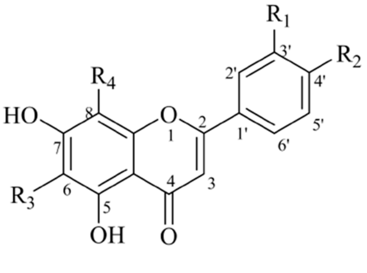

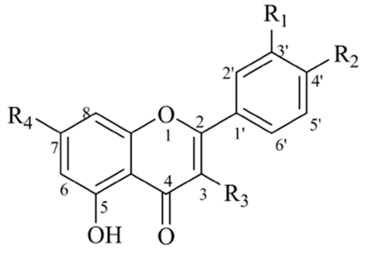

1 R_1=R_2=OH, R_3=R_4=Glc

2 R_1=H, R_2=OH, R_3=R_4=Glc

3 R_1=R_2=OH, R_3=Glc, R_4=Pen

4 R_1=O-CH_3, R_2=OH, R_3=Pen, R_4=Hex

5 R_1=R_2=OH, R_3=Glc, R_4=Pen

6 R_1=H, R_2=OH, R_3=Glc, R_4=Xyl

7 R_1=R_2=OH, R_3=Pen, R_4=Glc

8 R_1=H, R_2=OH, R_3=Glc, R_4=Ara

9 R_1=R_2=OH, R_3=Glc, R_4=H

10 R_1=R_2=OH, R_3=Xyl, R_4=Glc

11 R_1=R_2=OH, R_3=Rhm, R_4=Pen

12 R_1=R_2=OH, R_3=H, R_4=Glc

13 R_1=H, R_2=OH, R_3=Ara, R_4=Ara

14 R_1=R_2=OH, R_3=Pen, R_4=Rhm

15 R_1=O-CH_3, R_2=OH, R_3=Hex, R_4=Pen

17 R_1=O-CH_3, R_2=OH, R_3=Hex, R_4=Pen

19 R_1=H, R_2=OH, R_3=Glc, R_4=H

24 R_1=OH, R_2=OCH_3, R_3=*O*-Glc, R_4=H

32 R_1/R_2=O-CH_3, R_1/R_2=OH, R_3=R_4=Glc

33 R_1=OH, R_2=OCH_3, R_3=H, R_4=Glc

16 R_1=R_2=OH, R_3=*O*-Rhm-Glc, R_4=OH

18 R_1=R_2=OH, R_3=*O*-Rhm-(1→6)-Glc, R_4=OH

20 R_1=R_2=OH, R_3=*O*-Gal, R_4=OH

21 R_1=R_2=OH, R_3=*O*-Gln, R_4=OH

22 R_1=R_2=OH, R_3=*O*-Glc, R_4=OH

23 R_1=H, R_2=OH, R_3=OH, R_4=*O*-Gal

25 R_1=R_2=OH, R_3=H, R_4=*O*-Rut

26 R_1=H, R_2=OH, R_3=*O*-Rob, R_4=OH

27 R_1=OCH_3, R_2=OH, R_3=*O*-Neo, R_4=OH

28 R_1=OCH_3, R_2=OH, R_3=*O*-Rut, R_4=OH

29 R_1=OCH_3, R_2=OH, R_3=*O*-Gal, R_4=OH

30 R_1=OCH_3, R_2=OH, R_3=*O*-Glc, R_4=OH

31 R_1=H, R_2=OH, R_3=OH, R_4=*O*-Glc

34 R_1=OH, R_2=OCH_3, R_3=H, R_4=*O*-Rut

35 R_1=OCH_3, R_2=OH, R_3=H, R_4=*O*-Glc

36 R_1=H, R_2=OH, R_3=OH, R_4=OH

37 R_1=OH, R_2=OH, R_3=OH, R_4=OH

38 R_1=OH, R_2=O-CH_3, R_3=H, R_4=OH

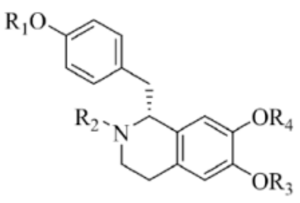

S1

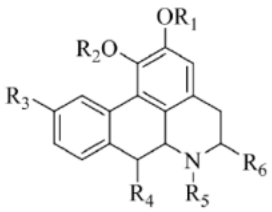

S2

S1	R_1	R_2	R_3	R_4
**39**	Glc	H	H	H
**40**	H	CH_3	H	H
**41**	H	H	Glc	H
**42**	H	H	H	H
**43**	CH_3	H	H	H
**47**	H	CH_3	CH_3	H
**48**	H	H	H	CH_3
**49**	H	CH_3	H	CH_3
**53**	H	CH_3	CH_3	CH_3
**55**	CH_3	CH_3	H	H
**56**	H	H	CH_3	H
**59**	CH_3	H	CH_3	CH_3

S2	R_1	R_2	R_3	R_4	R_5	R_6
**45**	CH_3	CH_3	H	OH	H	H
**58**	CH_3	CH_3	H	H	H	H
**61**	H	CH_3	O-CH_3	H	H	H
**63**	H	CH_3	H	H	CH_3	H
**64**	$R_1+R_2=CH_2$		H	H	CH_3	H
**65**	CH_3	CH_3	H	H	CH_3	H
**66**	$R_1+R_2=CH_2$		H	H	H	H
**68**	CH_3	CH_3	H	H	CH_3	OH

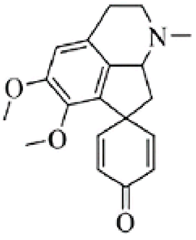

51

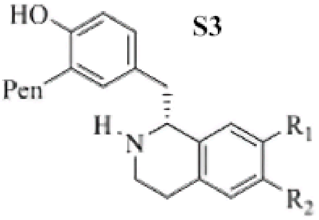

S3	R_1	R_2
**62**	OH	OCH_3
**67**	OCH_3	OH

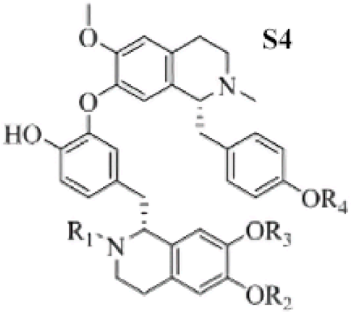

S4	R_1	R_2	R_3	R_4
**46**	CH_3	CH_3	H	H
**50**	H	H	CH_3	CH_3
**54**	CH_3	CH_3	CH_3	H
**57**	CH_3	CH_3	H	CH_3
**60**	CH_3	CH_3	CH_3	CH_3

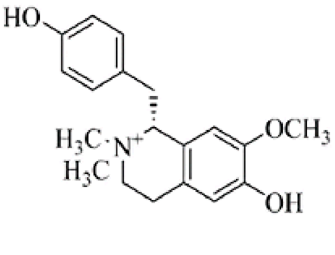

44

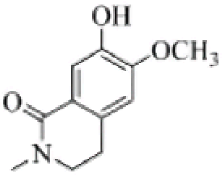

52

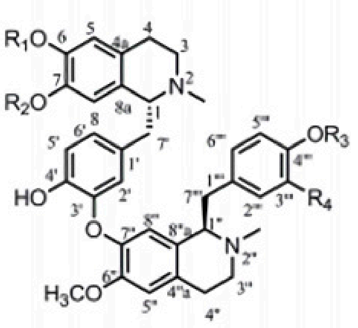

81 R_1=R_5=CH_3, R_2=R_3=R_4=H
84 R_1=R_3=R_4=H, R_2=R_5=CH_3
86 R_1=R_2=CH_3, R_3=R_4=R_5=H
87 R_1=R_2=R_5=CH_3, R_3=R_4=H
89 R_1=R_3=R_5=CH_3, R_2=R_4=H
90 R_1=R_3=R_5=CH_3, R_2=H, R_4=OH
92 R_1=R_2=R_3=R_5=CH_3, R_4=H
94 R_1=R_2=R_3=R_5=CH_3, R_4=OH

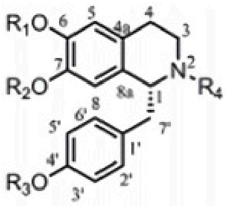

70 R_1=R_2=R_4=H, R_3=glucosyl
71 R_1=R_3=H, R_2=glucosyl, R_4=CH_3
72 R_1=R_2=H, R_3=glucosyl, R_4=CH_3
74 R_1=glucosyl, R_2=R_3=R_4=H
75 R_1=R_4=CH_3, R_3=H, R_2=glucosyl
or R_1=R_4=CH_3, R_2=H, R_3=glucosyl
76 R_1=R_2=R_3=R_4=H
77 R_1=R_2=R_3=H, R_4=CH_3
82 R_1=R_3=R_4=H, R_2=CH_3
83 R_1=CH_3, R_2=R_3=R_4=H
85 R_1=R_4=CH_3, R_2=R_3=H
88 R_1=R_3=R_4=CH_3, R_2=H
91 R_1=R_2=H, R_3=R_4=CH_3
93 R_1=R_2=R_4=CH_3, R_3=H

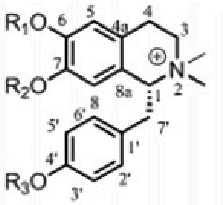

78 R_1=R_3=H, R_2=CH_3
79 R_1=CH_3, R_2=R_3=H
69 R_1=H, R_2=CH_3, R_3=glucosyl
73 R_1=CH_3, R_2=H, R_3=glucosyl

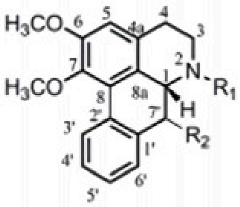

80 R_1=H, R_2=OH
95 R_1=CH3, R_2=H

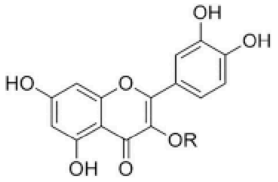

96 R=glucosyl-(1→2) -glucoside
104 R=galactoside
114 R=H
109 R=glucoside
112 R=glucuronic acid

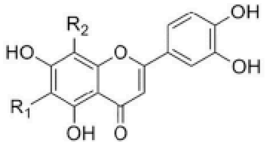

97 R_1=R_2=glucoside
98 R_1=pentoside, R_2=glucoside
101 R_1=glucoside, R_2=H
102 R_1=H, R_2=glucoside
111 R_1=R_2=H

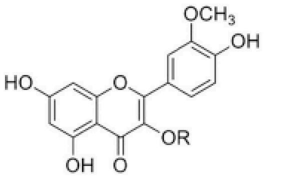

117 R=H
118 R=neohesperidoside

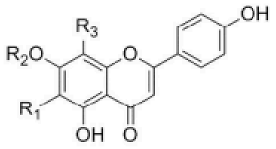

99 R_1=R_3=glucoside, R_2=H
103 R_1=glucoside, R_2=H, R_3=rhamnoside
106 R_1=glucoside, R_2=R_3=H
107 R_1=rhamnoside, R_2=H, R_3=glucoside
110 R_1=R_2=H, R_3=glucoside
119 R_1=R_3=H, R_2=neohesperidoside

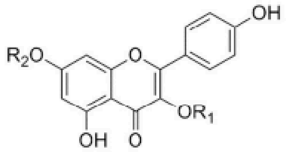

100 R_1=neohesperidosides, R_2=glucoside
113 R_1=rutinoside, R_2=H
115 R_1=R_2=H
116 R_1=neohesperidoside, R_2=H
123 R_1=galactoside, R_2=H
124 R_1=glucoside, R_2=H

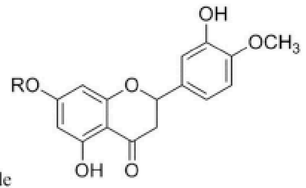

105 R=rutinoside

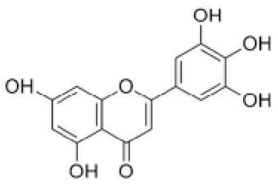

108 Trihydroxyflavone (Tricetin)

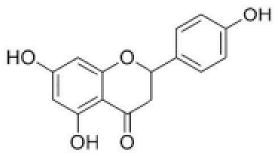

120 Naringenin

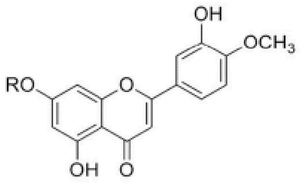

121 R=H
122 R=neohesperidoside

Figure S57.The chemical structures of *Nelumbo nucifera* gaertn
